# Development and Application of Cationic Nile Blue Probes in Live-Cell Super-Resolution Imaging and Specific Targeting to Mitochondria

**DOI:** 10.1021/acscentsci.4c00073

**Published:** 2024-05-17

**Authors:** Yunsheng Li, Xiaoyu Bai, Dan Yang

**Affiliations:** † School of Life Sciences, Westlake University, Hangzhou 310024, China; ‡ Morningside Laboratory for Chemical Biology, Department of Chemistry, The University of Hong Kong, Hong Kong 999077, China; § Westlake Laboratory of Life Sciences and Biomedicine, Hangzhou 310024, China

## Abstract

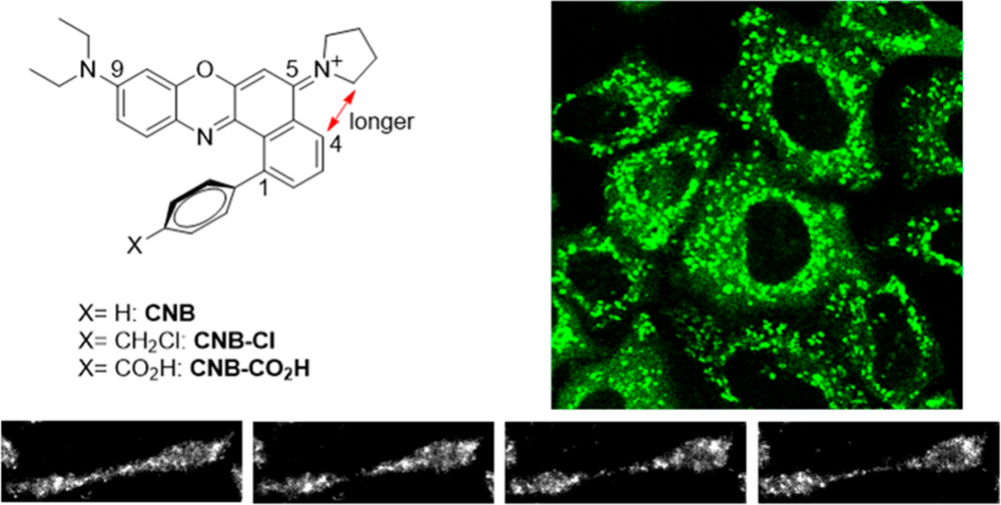

Mitochondria are essential organelles involved in various metabolic processes in eukaryotes. The imaging, targeting, and investigation of cell death mechanisms related to mitochondria have garnered significant interest. Small-molecule fluorescent probes have proven to be robust tools for utilizing light to advance the study of mitochondrial biology. In this study, we present the rational design of cationic Nile blue probes carrying a permanent positive charge for these purposes. The cationic Nile blue probes exhibit excellent mitochondrial permeability, unique solvatochromism, and resistance to oxidation. We observed weaker fluorescence in aqueous solutions compared to lipophilic solvents, thereby minimizing background fluorescence in the cytoplasm. Additionally, we achieved photoredox switching of the cationic Nile blue probes under mild conditions. This enabled us to demonstrate their application for the first time in single-molecule localization microscopy of mitochondria, allowing us to observe mitochondrial fission and fusion behaviors. Compared to conventional cyanine fluorophores, this class of dyes demonstrated prolonged resistance to photobleaching, likely due to their antioxidation properties. Furthermore, we extended the application of cationic Nile blue probes to the mitochondria-specific delivery of taxanes, facilitating the study of direct interactions between the drug and organelles. Our approach to triggering cell death without reliance on microtubule binding provides valuable insights into anticancer drug research and drug-resistance mechanisms.

## Abstract 2

Fluorescent cationic Nile blue probes were developed for SMLM imaging of mitochondria in living cells and the specific targeting of the antitumor drug paclitaxel into mitochondria.

## Introduction

Mitochondria play critical roles in various cellular processes, including energy supply, apoptosis regulation, and reactive oxygen species (ROS) generation, making the study of their dynamics, fine structures, and complex biological functions of great interest.^1,2^ To visualize mitochondria, researchers have utilized delocalized lipophilic cations^3^ such as triphenylphosphonium (TPP), rhodamine or cyanine dyes, and mitochondria-penetrating peptides (MPP),^4^ taking advantage of the negative inner membrane potential (ΔΨ_m_) of this organelle. However, the lack of fluorescence exhibited by these dyes makes tracing their subcellular localization challenging. In fluorescence microscopy, the near-infrared (NIR) wavelength range (650–900 nm) offers minimal background interference and low photodamage effects on live cells,^5^ making it particularly advantageous. Despite the availability of various cationic fluorophores for imaging mitochondria, those in the NIR region have predominantly been limited to cyanine dyes. The small Stokes shift (10–15 nm) of cyanine dyes often leads to self-quenched emission in multiply labeled molecules due to homotransfer,^6^ and their polymethine chain structure renders them prone to oxidation. Recently, a water-soluble NIR perylene fluorophore was reported to selectively stain mitochondria through conjugation with TPP.^7^ However, its relatively high dye dosage requirement and observed high background noise suggest a possible trade-off between membrane permeability, labeling efficiency, and water solubility.

Single-molecule localization microscopy (SMLM) encompasses a collection of super-resolution fluorescence microscopy techniques that enable the visualization of cellular structures with resolutions down to a few nanometers. These techniques involve precise yet stochastic localization of dye molecules in their fluorescent “on” state while keeping the majority of dyes in the “off” state.^8^ Over the past 17 years, several small-molecule fluorophores have been demonstrated for SMLM imaging of cellular structures.^8−11^ However, fluorophores with overall negative charges, such as fluorescein, ATTO 488, Cy5, and Alexa 647, exhibit good water solubility but lack cell permeability, restricting their use to cell membrane labeling. Inside-cell labeling requires electroporation or essential transfection techniques. While cyanine fluorophores have been commonly used in SMLM, photobleaching remains a significant limitation, affecting spatial and temporal resolutions. Additionally, hypsochromic phototruncation, which involves oxidative cleavage of one ethylene by singlet oxygen to form “two-carbon truncated” cyanine, introduces concerns about photoblueing artifacts in multicolor imaging.^12,13^ Efforts to improve the photostability of cyanine dyes include the removal of molecular oxygen using oxygen scavenger systems and the use of reducing additives (thiols, ascorbic acid, Trolox, etc.) in the imaging buffer. However, these approaches may have potentially toxic side effects in live cells. Despite the tremendous interest in developing new fluorophores for SMLM of mitochondria in live cells, to date, only a few fluorophores have been demonstrated for this purpose without the need for transfection, essential bioconjugation, or specific imaging buffers.^7,14−18^

Unlike cyanine dyes, Nile blue belongs to the oxazine dye family and possesses an additional phenyl ring fused to the oxazine structure, resulting in a compact, aromatic structure that contributes to its high stability and intense fluorescence. In this study, we present the development of cationic Nile blue probes as novel imaging and targeting tools for mitochondria. For the first time, we demonstrate their application in single-molecule localization microscopy of mitochondria, allowing us to observe the fission and fusion behaviors of mitochondria under physiological conditions without the need for reducing additives or oxygen scavenger systems. The excellent mitochondria-targeting properties of cationic Nile blue probes also enable their use in the organelle-specific delivery of anticancer drugs, such as taxanes, for studying drug–organelle interactions.

## Results and Discussion

### Development of Cationic Nile Blue Dyes As Novel Mitochondrial Targeting Probes

**Scheme 1:**
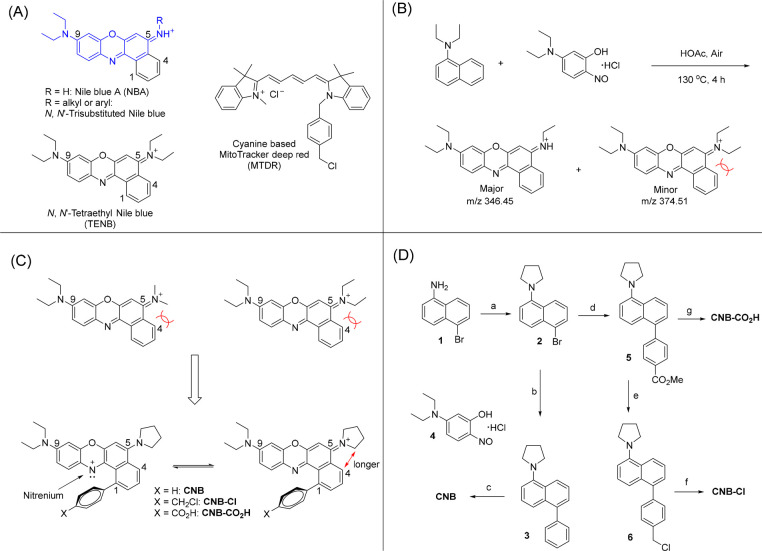
Design and Synthesis of Cationic Nile Blue Type of Fluorophores: (A) Derivatization of Nile Blue in the Literature and the Structures of Tetraethyl Nile Blue and MitoTracker Deep Red (MTDR); (B) Trial in Synthesis of Tetraethyl Nile Blue; (C) Design of Cationic Nile Blue Type of Fluorophores; (D) Synthesis of Cationic Nile Blue Conditions: a, 1,4-dibromobutane, K_2_CO_3_, KI, DMF, 100 °C, 12 h, 81%; b, phenylboronic acid, Pd(dppf)Cl_2_, NaHCO_3_, H_2_O, dioxane, 75 °C, 3 h, 98%; c, **4**, acetic acid, 115 °C, 8 h, 50%; d, 4-methoxycarbonylphenylboronic acid, Pd(dppf)Cl_2_, NaHCO_3_, H_2_O, dioxane, 75 °C, 6 h, 92%; e, (1) LiAlH_4_, Et_2_O, rt, 1 h; (2) PPh_3_, CCl_4_, reflux, 28 h, 65% from **5**; f, **4**, acetic acid, 115 °C, 6 h, 31%; g, (1) 3 N NaOH, THF, reflux; (2) **4**, acetic acid, 115 °C, 8 h, 41% from **5**.

In the development of cationic Nile blue dyes as mitochondrial targeting probes, previous research has mainly focused on modifications of the three substituents on the 5/9-amino groups of Nile blue (referred to as *N*,*N*′-trisubstituted Nile blue in Scheme 1A).^19,20^ However, these modifications often lead to deprotonation and subsequent blueshift of absorption,^21^ limiting their applicability. Additionally, these dyes passively diffuse into lysosomes and other cellular components,^22,23^ further complicating their use. In contrast, *N*,*N*′-tetrasubstituted Nile blue fluorophores, which function as delocalized cations suitable for mitochondrial imaging, have received little attention in the literature.^24^ Characterization of this dye type has been restricted to melting point and mass measurements, with no further evaluation in biological systems. Our attempts to prepare *N*,*N′*-tetrasubstituted Nile blue dyes revealed their susceptibility to nucleophilic attack, which not only complicates their chemical synthesis but also renders them unstable in live cells (Scheme 1B, Schemes S1 and S2, Figures S1–S3).

To obtain stable cationic Nile blue fluorophores, we made several modifications (Scheme 1C). First, we replaced the 5-amino group of Nile blue A with a pyrrolidine ring. This modification was chosen because the pyrrolidine ring is less polar and more lipophilic than NH_2_. In contrast to tetraethyl Nile blue (TENB), the modified cationic Nile blue with a pyrrolidine ring does not experience severe steric repulsion between the CH group at position C4 and the CH_2_ group of the pyrrolidine ring. The conformational constraints of the pyrrolidine ring alleviate the steric interactions and allow for a more favorable arrangement of atoms in the molecule. Unlike trisubstituted Nile blue, which has an extra proton, the modified structure is less sensitive to environmental pH. Additionally, the installation of a phenyl ring at position C1 shields the unique nitrenium, making it less susceptible to nucleophilic attack. Moreover, the phenyl ring can serve as a platform for attaching protein binding agents or drugs for mitochondrial delivery.

We developed a unified synthesis procedure for cationic Nile blue (**CNB**) and its derivatives (Scheme 1D). This concise and high-yielding synthesis (3–5 steps) allows rapid access to these dyes. The key transformation involves the acetic acid-catalyzed condensation between amino naphthalene and aromatic nitroso compounds, both of which can be readily prepared from commercially available compounds. However, attempts to further contract the pyrrolidine ring on **CNB** to azetidine or aziridine were unsuccessful, likely due to a dramatic increase in ring strain and instability.

**Figure 1:**
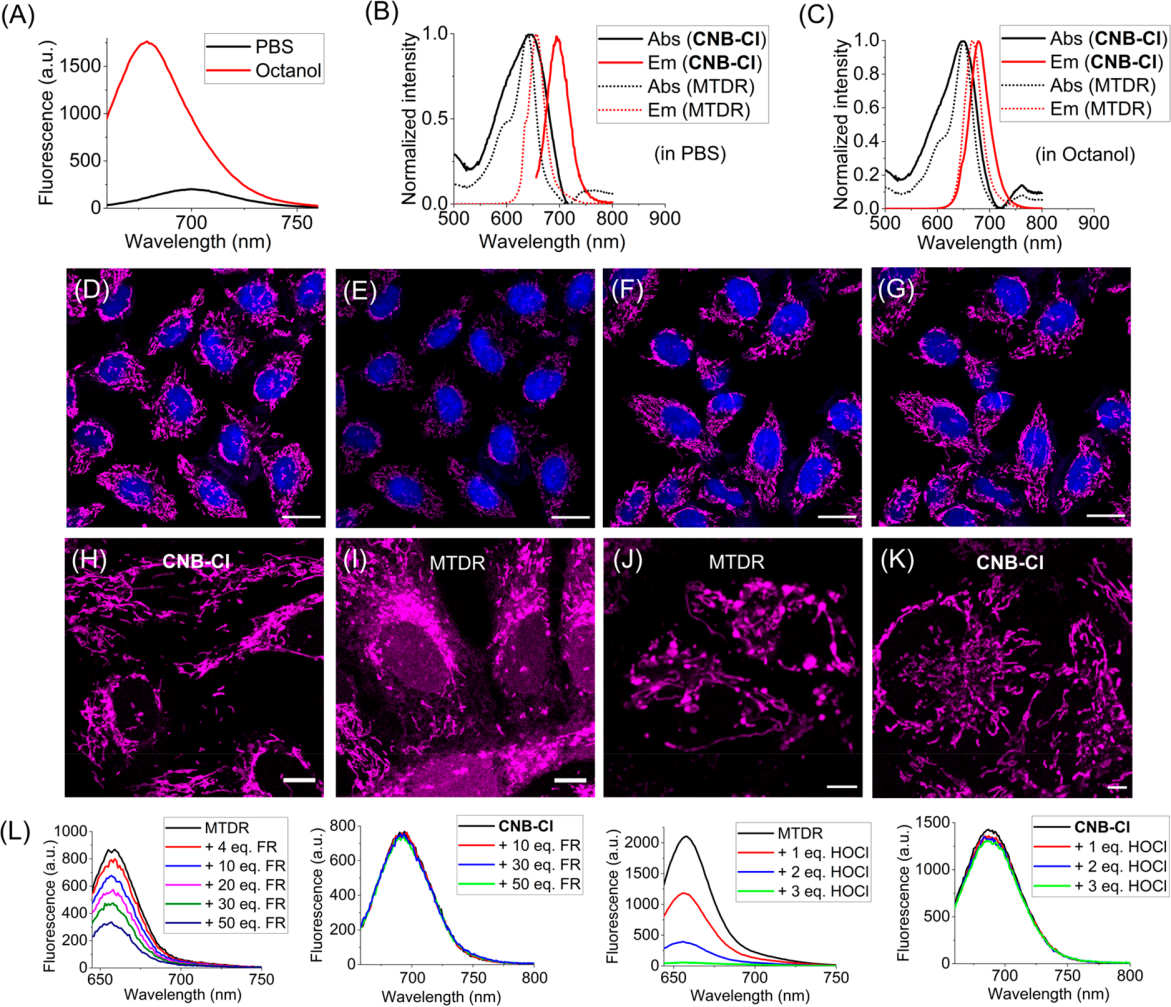
Evaluation of fluorescent properties of cationic Nile blue fluorophores and their applications in live-cell imaging. The fluorescent dyes for mitochondria imaging were excited at 633 nm wavelength, and the fluorescence emission was collected from 640 to 750 nm. (A) Approximately 8-fold fluorescence enhancement of **CNB-Cl** in octanol compared to PBS. (B, C) Absorption and emission spectra of **CNB-Cl** and MTDR in PBS and octanol. (D–G) Evaluation of lipophilicity of the surroundings of **CNB** in live cells. The fluorescence intensity collected at 650–700 nm (D) is significantly stronger than that collected at 700–750 nm (E). However, the intensity of fluorescence collected at 640–680 nm (F) is comparable to that collected at 680–720 nm (G). (H) Live-cell imaging with **CNB-Cl** (500 nM) without washing steps showed a low background signal. (I) Live-cell imaging with MTDR (500 nM) without washing steps showed a high background signal. (J) Swelling and enlargement of mitochondria were frequently observed in cells treated with MTDR (250 nM, 24 h). (K) Filamentous mitochondria largely remained in cells treated with **CNB-Cl** (250 nM, 24 h). (L) The fluorescence of MTDR but not **CNB-Cl** can be quenched by HO^•^ and HOCl. The Fenton reagent (FR), a combination of Fe^2+^ and H_2_O_2_, is used to generate HO^•^ in situ. Scale bars: 20 μm for (D)–(G), 10 μm for (H)–(J), and 5 μm for (K).

Unlike ordinary oxazine dyes, cationic Nile blue exhibits unique solvatochromism (Figure 1A). It demonstrates over 8-fold enhancement of fluorescence in lipophilic media (e.g., octanol) compared to aqueous media. In aqueous buffer, **CNB** shows a larger Stokes shift (40–50 nm) and red-shifted emission (∼700 nm), while this phenomenon is less significant in lipophilic solvents such as octanol (Figure 1B and C). In contrast, the absorption/emission profile of MitoTracker deep red (MTDR), a specific cyanine derivative, remains largely unchanged in both PBS and octanol. The stability of cationic Nile blue probes toward bioanalytes (Figure S4) and the quantum yields of those probes (Table S1) were also measured.

We conducted live-cell imaging experiments to evaluate the suitability of **CNB** for cellular imaging. Poor cell permeability is a common limitation of fluorescent probes in living systems, often requiring invasive techniques or high probe concentrations to facilitate membrane crossing. However, we were pleased to find that **CNB** exhibited excellent cell permeability and specific accumulation in mitochondria, even at concentrations as low as 25 nM. Importantly, we did not observe any nonspecific staining of lysosomes or the nucleus, which was in contrast to the behavior of NBA (a trisubstituted Nile blue derivative) (Figures S5 and S6). The accumulation of **CNB** in mitochondria was dependent on the mitochondrial membrane potential (ΔΨ_m_), as treatment with the uncoupling agent carbonyl cyanide *p*-trifluoromethoxyphenylhydrazone (FCCP) caused a decrease in fluorescence signal (Figure S6). To anchor **CNB** inside mitochondria, we attached a thiol-reactive chloromethyl group to the phenyl ring, resulting in **CNB-Cl**. **CNB-Cl** also exhibited selective accumulation in mitochondria (Figure S7) and retained fluorescence signal even after the collapse of ΔΨ_m_ (Figure S8).

The solvatochromism exhibited by cationic Nile blue dyes allowed us to assess the lipophilicity of the dye’s surroundings within live cells. Upon incubation of HeLa cells with **CNB**, we observed that the fluorescence collected from the 650–700 nm range was significantly stronger than that collected from the 700–750 nm range. Furthermore, the fluorescence collected from the 640–680 nm range was comparable to that collected from the 680–720 nm range (Figure 1D–G). This indicates that cationic Nile blue has a fluorescence maximum at around 680 nm in live cells. Consequently, its fluorescence primarily originates from an environment that is more “octanol-like” rather than “PBS-like”, as evidenced by the red-shifted emission (∼700 nm) in PBS compared to the emission (∼680 nm) in a lipophilic solvent (Figure 1A–C).

Except for staining live cells, **CNB** is also well suited for mitochondria of distinct morphology in live *Caenorhabditis elegans*.^25^ By using fluorescence microscopy and **CNB** dye, we observed donut-shaped mitochondria in old *C. elegans*.^25^ Those aged mitochondria could be transmitted to offspring and restore vitality in the offspring’s early life, characterized by gradual restoration of the elongated tubular morphology.

Small-molecule dyes with fluorogenic properties offer reduced background fluorescence and high imaging contrast, as they only become fluorescent upon binding to specific biomolecular targets. This selective fluorescence allows “off-target” probes to remain non- or weakly fluorescent.^26,27^ In a similar vein, **CNB-Cl**, with its solvatochromism and excellent membrane permeability, exhibited low background signal, as observed through Z-stack scanning (Figure S9). We observed low background signal at various concentrations of **CNB-Cl** (Figures 1H and S10) and **CNB** (Figure S11) in live-cell imaging without the need for washing steps. In contrast, the diffusion of MTDR into the cytosol and nucleus was clearly observed (at 500 nM, Figure 1I). This demonstrates the ability of **CNB-Cl** to provide high imaging contrast and minimize background fluorescence.

Mitochondria, being highly sensitive to external stimuli, were of primary interest in our examination of potential damage caused by **CNB-Cl** and MTDR. HeLa cells were treated with 250 nM of each probe for a period of 24 h. Notably, we frequently observed swelling and enlargement of mitochondria within MTDR-treated cells. Conversely, the mitochondria within **CNB-Cl**-treated cells largely preserved their filamentous morphology (Figure 1J and K). When the concentration of the probes was reduced to 100 nM, the observed phenomenon of mitochondrial swelling was less pronounced (Figure S12). Mitochondrial swelling can result from an osmotic imbalance between the cytosol and the matrix, leading to increased water influx.^28^ Our results indicate that **CNB-Cl** exhibits minimal perturbation to the native environment of mitochondria, making it superior to MTDR in terms of preserving mitochondrial morphology.

Mitochondrial respiratory metabolism generates ROS, including hypochlorous acid (HOCl) and hydroxyl radical (HO^•^), which are highly reactive.^29,30^ The interaction between ROS generated in situ and dyes at different excited states is a common chemical process that can lead to irreversible photobleaching. Therefore, the antioxidant properties of probes are crucial for mitochondrial imaging. In our study, we observed that HO^•^ could oxidatively quench the fluorescence of MTDR in a concentration-dependent manner, but it had minimal effect on the fluorescence of **CNB-Cl**. Furthermore, three equivalents of HOCl completely bleached MTDR, while causing only minimal fluorescence loss to **CNB-Cl** (Figure 1L). The remarkable stability of **CNB-Cl** toward oxidation can likely be attributed to the nitrenium group in its resonance structure (Scheme 1C). The higher electronegativity of this nitrogen atom lowers the electron density of the conjugated system, providing increased resistance to oxidation.

### Application of Cationic Nile Blue in SMLM of Mitochondria

The compatibility of small-molecule fluorophores with super-resolution SMLM can greatly expand their applications. As a new class of small-molecule NIR fluorophores, cationic Nile blue offers the opportunity for transfection-free, super-resolution imaging of mitochondria. In SMLM, background noise can arise from nonspecific accumulation, cellular autofluorescence, or photobleaching, where detected photons may not necessarily originate from dyes bound to their targets. However, we anticipate that the solvatochromism, NIR absorption/emission properties, and resistance to oxidation exhibited by **CNB-Cl** would help diminish these types of background noise in SMLM imaging.

**Figure 2:**
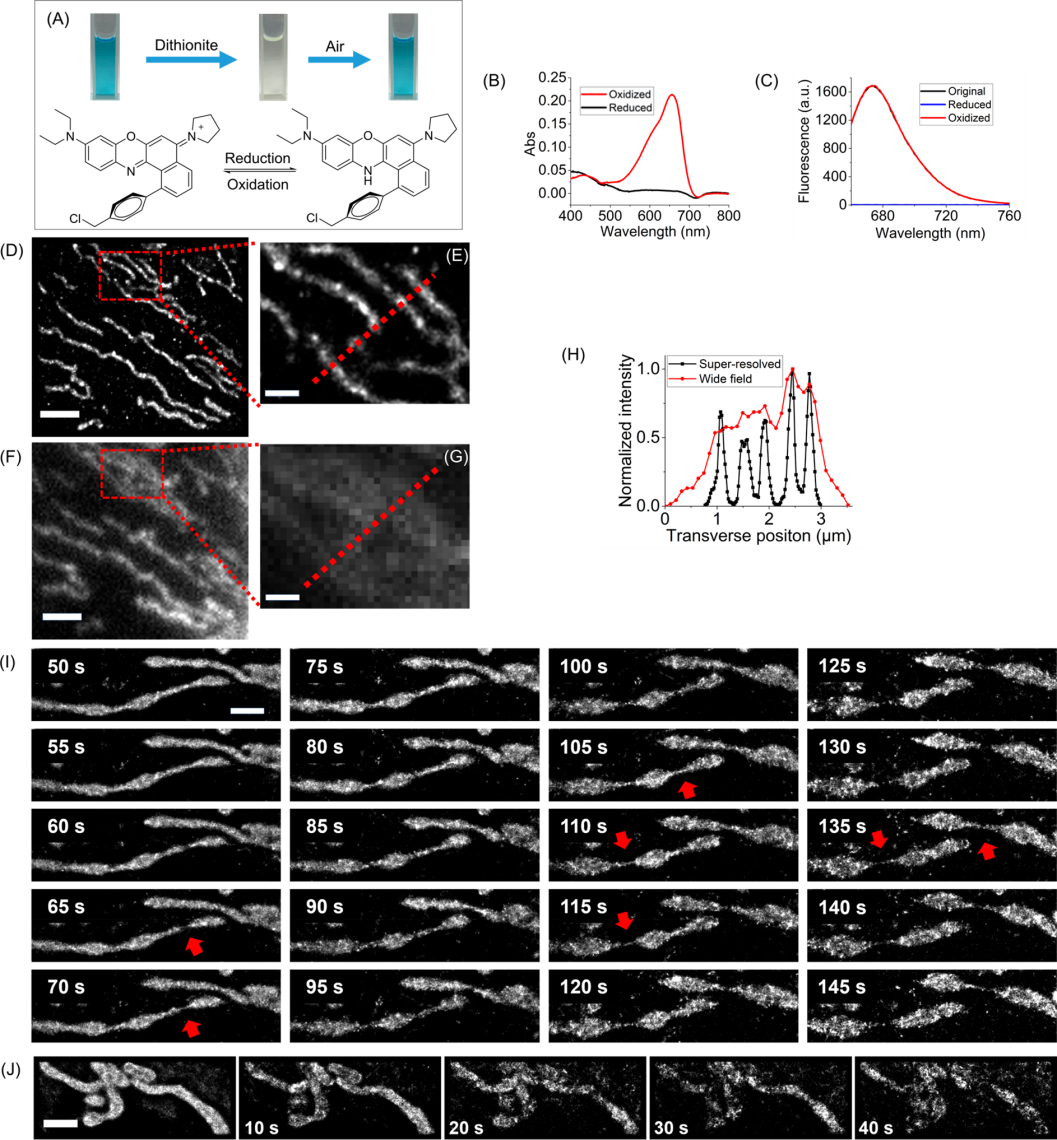
Evaluation of cationic Nile blue probe **CNB-Cl** in SMLM of mitochondria. (A) **CNB-Cl** was reduced by sodium dithionite (Na_2_S_2_O_4_) and reoxidized by exposure to air. (B, C) Absorption and emission spectra of oxidized and reduced forms of **CNB-Cl**, respectively. (D, E) Reconstructed, super-resolution images of mitochondria in fixed cells using **CNB-Cl**. (F, G) Wide-field images of mitochondria captured immediately before SMLM imaging. (H) The transverse profiles of five single mitochondria marked with a red dotted line in a reconstructed super-resolution image (E) and a wide-field image (G). (I) Mitochondria dynamics in live cells revealed by **CNB-Cl** with SMLM. Each image was reconstructed with 1000 consecutive frames at a rate of 100 frame/s. Multiple fission and fusion (red arrows) events were identified. (J) Live-cell SMLM imaging of mitochondria with MTDR showed fast photobleaching and a high background. Scale bars: 2 μm in (D)/(F)/(I)/(J) and 500 nm in (E)/(G).

We conducted a comparison of the ground-state redox properties of **CNB-Cl** with other probes used for mitochondrial imaging.^31^ This comparison is crucial, as it determines the feasibility of switching the dyes from a fluorescent “on” state to a dark state. Initially, we thoroughly mixed 10 μM **CNB-Cl** in dichloromethane with a freshly prepared sodium dithionite (Na_2_S_2_O_4_) solution. This resulted in the complete disappearance of the characteristic greenish color of **CNB-Cl**, as well as its NIR absorption/emission, indicating the formation of the reduced form of the dye (Figure 2A). Importantly, when we introduced air by bubbling it through the yellowish dichloromethane phase, we observed a gradual recovery of the greenish color and the intensity of absorption/emission without any loss (Figure 2B,C and Supplementary Video 1). This phenomenon of oxidation by air is recognized as one of the key transformations in the synthesis of Nile blue dyes (Scheme S1).

The redox switching exhibited by **CNB-Cl** was found to be reversible and could be repeated more than 10 times before significant degradation occurred. In contrast, we observed that MitoTracker probes based on rhodamine or cyanine dyes were unable to achieve such spontaneous redox switching. This is likely due to their low-lying reduction potentials,^32^ indicating their reluctance to accept electrons. Instead, these probes required stronger reducing agents (such as sodium borohydride) or high laser intensities to switch them into dark states.^14,33,34^ Other oxazine fluorophores, such as Atto 655, were also found to be redox switchable under physiological conditions.^35^ However, their limited membrane permeability and tendency for nonspecific staining hindered their effectiveness in penetrating and imaging mitochondria.

To evaluate the suitability of **CNB-Cl** for SMLM, we first assessed its performance in ROXS buffer^36^ in fixed U-2 OS cells. After illuminating the probe with a low-intensity single 647 nm laser (<3 kW/cm^2^) for several seconds, the probe started to exhibit blinking behavior (Supplementary Video 2). A super-resolved image was reconstructed using 9000 frames, resulting in enhanced resolution (Figure 2D and E). The transverse profiles of five individual mitochondria, indicated by red dotted lines in the reconstructed image, revealed an average full width at half-maximum (fwhm) of approximately 250 nm. This value was significantly smaller than that obtained from diffraction-limited total internal reflection fluorescence (TIRF) imaging (Figure 2H) and confocal laser scanning microscopy (∼500 nm, Figure S13). On average, each probe emitted 680 photons per imaging frame (Figure S14), enabling the localization of single molecules with an uncertainty of approximately 13 nm (Figure S14). This represents a more than 1 order of magnitude improvement in lateral resolution compared to conventional light microscopy techniques.

In live cells, the photoreduction efficiency of organic fluorophores is determined by the local concentration of thiols and the pH value, as thiolate (RS^–^) serves as the reductive species.^32^ Studies on intracellular glutathione (GSH) have revealed that mitochondria have the most reductive GSH/GSSG pool due to their alkaline interior with a pH of approximately 7.8.^37^ This unique feature of mitochondria makes it possible to perform live-cell SMLM imaging using **CNB-Cl**. To achieve this, U-2 OS cells were incubated with 100–250 nM **CNB-Cl**, washed, immersed in DMEM, and then subjected to SMLM imaging. There was no need for the addition of reducing thiols or an oxygen scavenger system. This allowed us to study mitochondrial dynamics under physiological conditions, and we successfully observed multiple fission and fusion events, as indicated by the red arrows (Figure 2I). Long and thin structures were formed before fusion or after fission and could persist for several seconds. These structures are believed to be constricted by the dynamin-family protein Drp1 (dynamin-related protein 1), which is essential for the division of mammalian mitochondria.^38,39^ Each dye molecule emitted an average of 800 photons per imaging frame, enabling the localization of a single **CNB-Cl** probe with a precision of approximately 18 nm (Figure S15). In live cells, around 10% more photons were collected compared to fixed samples, likely due to the well-preserved membrane integrity of mitochondria in live cells, creating a highly lipophilic environment that enhances the fluorescence brightness of **CNB-Cl**. In addition to U-2 OS cells, **CNB-Cl** has also been demonstrated to be effective for live-cell SMLM imaging of mitochondria in other cell lines. Specifically, we have successfully used **CNB-Cl** for imaging mitochondria in COS-7 cells (Supplemental Video 3) and HeLa cells (Supplemental Video 4 and Figure S16).

Although there is a wide range of cationic fluorophores available for imaging mitochondria in live cells, the options in the NIR region are mostly limited to the cyanine family. In our study, we compared the performance of **CNB-Cl** with the cyanine-based MTDR for live-cell SMLM imaging of mitochondria. Under identical imaging conditions to **CNB-Cl**, super-resolved images could be reconstructed using the initial 3000 frames for MTDR. However, we observed that MTDR underwent rapid photobleaching during the imaging process, as illustrated in Figure 2J, resulting in eventually background-dominated reconstructed images. In contrast, the blinking behavior of **CNB-Cl** persisted for a significantly longer period (Figure 2I). The mechanism behind photobleaching is complex, but evidence suggests that in the presence of molecular oxygen, especially in the case of cyanine dyes,^40^ the triplet state of the dye can be easily oxidized, leading to photobleaching. Without the addition of a large amount of reducing thiols, the subsequent reduction to repopulate the ground state is hindered. Our experiments support this notion, as we observed fast and complete ground-state oxidation of MTDR by ROS, while reduction by dithionite was unachievable. Therefore, an oxygen scavenger system must be employed for MTDR imaging or triplet-state quenchers could be covalently attached onto the molecule of cyanine.^14,40,41^ In contrast, due to its higher reduction potential, **CNB-Cl** is more readily subjected to photoreduction, and the lifetime of radical ions is shortened to prevent rapid photobleaching.

### Application of Cationic Nile Blue in Organelle-Specific Delivery of Taxanes

**Figure 3:**
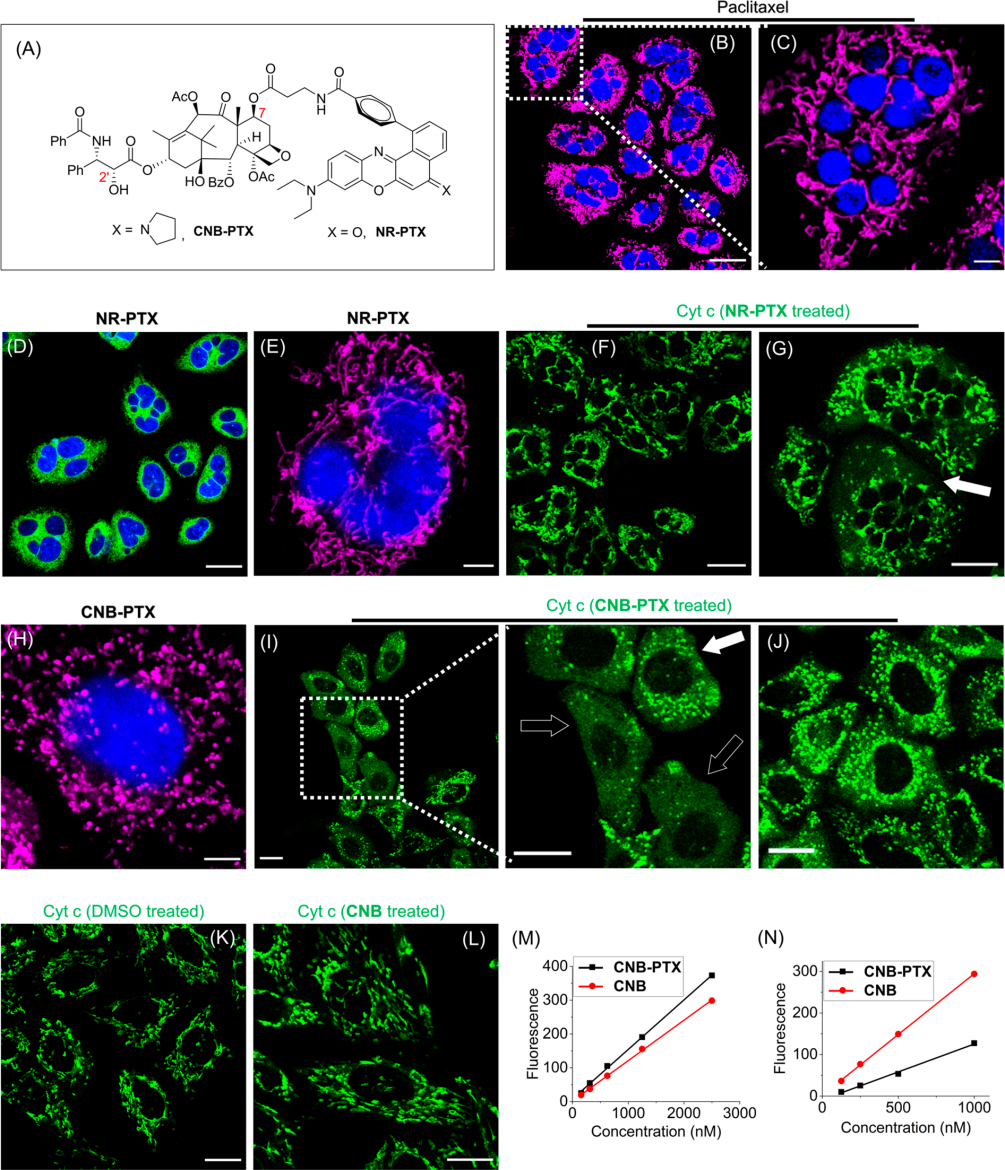
Evaluation of pro-apoptotic effects of taxane derivatives on HeLa cells. (A) Structures of taxane derivatives. (B, C) Nucleus fragmentation induced by paclitaxel (8 nM, 24 h). (D, E) Nucleus fragmentation induced by **NR-PTX** (250 nM, 24 h). Mapping the cyt c distribution of cells treated by 250 nM (F) or 500 nM of **NR-PTX** (G) for 24 h with cyt c monoclonal antibody–Alexa Fluor 488 conjugates. (H) Normal nuclear morphology but fragmented mitochondria of cells treated by **CNB-PTX** (2 μM, 24 h). Cyt c distribution of cells treated by 2 μM (I) or 500 nM (J) **CNB-PTX** for 24 h. Black arrows in these figures indicate cyt c was almost completely released into the cytosol, while white arrows indicate it was partially released. (K) Cyt c distribution in DMSO-treated cells. (L) Cyt c distribution of cells treated by 500 nM **CNB** for 24 h. (M) Fluorescence calibration curve of **CNB** and **CNB-PTX** in 95% EtOH. (N) Fluorescence analysis of cellular uptake of **CNB** and **CNB-PTX** after 6 h incubation. Excitation wavelength: 633 nm for **CNB**, **CNB-Cl**, **CNB-PTX**, and MTDR; 561 nm for MTR and **NR-PTX**; 488 nm for cyt c monoclonal antibody–Alexa Fluor 488 conjugates, LTG, and MTG; 405 nm for Hoechst. Scale bars, (C)/(E), 5 μm; (G)/(H), 10 μm; others, 20 μm.

Mitochondria play a crucial role in the activation of apoptotic effector mechanisms by regulating the translocation of pro-apoptotic proteins from the mitochondrial intermembrane space to the cytosol. Antitumor drug taxanes (such as paclitaxel, docetaxel, and derivatives) have been proposed to induce cell death by directly affecting mitochondria. This hypothesis is supported by the observation of cytochrome c (cyt c) release after incubating isolated mitochondria with high concentrations of paclitaxel.^42^ However, it has been challenging to demonstrate the relevance of this phenomenon in whole cells due to the inevitable interaction of paclitaxel with ubiquitous microtubules in the cytoskeleton. Previous attempts to deliver taxanes specifically to mitochondria using delocalized lipophilic cations decorated liposomes had limitations in terms of subcellular trafficking and releasing dynamics, hindering true molecular-level targeting.^43,44^ Therefore, it became necessary to reinvestigate the distinct interaction between taxanes and mitochondria, separate from the disruption of microtubules. To address this, cationic Nile blue was employed as a mitochondria-permeable molecule for specific delivery of taxanes. Some research groups have reported taxanes conjugated with TPP and rhodamine via esterification at the C2′ hydroxyl group.^45−47^ However, this method may significantly reduce the binding affinity of taxanes to microtubules.^48^ Therefore, an alternative approach was adopted, esterifying the hydroxyl group at C7 instead of C2′, which was expected to retain the activity of the parent drug (**CNB-PTX**, Figure 3A).^49,50^ For comparison and as a microtubule-targeting control, Nile red was used in place of **CNB** by changing pyrrolidine to oxygen (**NR-PTX**, Figure 3A). Since the modification site is far from the taxane moiety, the two derivatives are believed to retain comparable affinity for tubulin while differing only in subcellular accumulation.

The effective and precise delivery of the designed small molecules was confirmed by fluorescence microscopy (Figure S17). Cell viability assays conducted at 48 h indicated that the microtubule-targeting probe, **NR-PTX**, retained paclitaxel’s cytotoxicity reasonably well (IC_50_ = 145 nM, Figure S18), consistent with previous reports on fluorescent taxoids.^49^ The specific delivery of taxanes to mitochondria by **CNB** also exhibited good cytotoxicity (IC_50_ = 543 nM for **CNB-PTX**, Figure S18). Taxanes have been suggested to induce a specific form of apoptosis called mitotic catastrophe, characterized by nuclear fragmentation, such as multinucleation and micronucleation.^51,52^ As expected, mitotic catastrophe occurred smoothly in cells treated with paclitaxel or **NR-PTX** for 24 h at concentrations around 2-fold of the IC_50_ (8 nM for taxane and 250 nM for **NR-PTX**, Figure 3B–E). In these conditions, it was common to observe a single cell containing multiple small-sized nuclei. However, cells treated with **CNB-PTX** at a high concentration of 2 μM were predominantly mononuclear (Figure 3H), indicating that mitotic catastrophe did not occur. This can be explained by the fact that **CNB-PTX** does not disrupt the dynamics of microtubules in the cytoskeleton. Unexpectedly, we observed excessive fragmentation of mitochondria in cells treated with **CNB-PTX** (2 μM, 24 h; Figure 3H). This phenomenon was not present in cells treated with either taxane or **NR-PTX**, as shown in Figure 3C and E.

Mitochondrial fragmentation is closely associated with the release of the pro-apoptotic factor cyt c and apoptosis initiation.^38,53^ To investigate the release of cyt c from mitochondria, immunostaining with cyt c monoclonal antibody–Alexa Fluor 488 conjugates was performed. It was found that 2 μM **CNB-PTX** efficiently induced the release of cyt c into the cytosol (Figure 3I) compared to the DMSO control (Figure 3K). Decreasing the concentration of **CNB-PTX** to 500 nM was less effective but still induced intensive mitochondrial fission (Figure 3J). Cells treated with **NR-PTX** (250 nM, 24 h) exhibited extensive fragmentation of the nucleus (Figure 3F), consistent with the previously observed results, while the release of cyt c into the cytosol was not obvious. Increasing the concentration of **NR-PTX** to 500 nM gradually led to mitochondrial fission and cyt c release (Figure 3G). This indicates that the release of cyt c occurs after nuclear fragmentation in cells treated with **NR-PTX**. Additionally, **CNB** itself at a concentration of 500 nM was not efficient in inducing mitochondrial fragmentation and cyt c release (Figure 3L). Furthermore, the cellular uptake of **CNB-PTX** was quantified and compared to that of **CNB** through ethanol extraction followed by fluorescence analysis. After a 6 h incubation, it was found that the cellular uptake of **CNB-PTX** was 3-fold lower than that of **CNB** across a wide range of concentrations (Figure 3M,N). This suggests that the fragmentation of mitochondria and the release of cyt c induced by **CNB-PTX** are a result of the interaction of its taxane moiety, rather than the cationic fluorophores, with the mitochondria.

**Figure 4:**
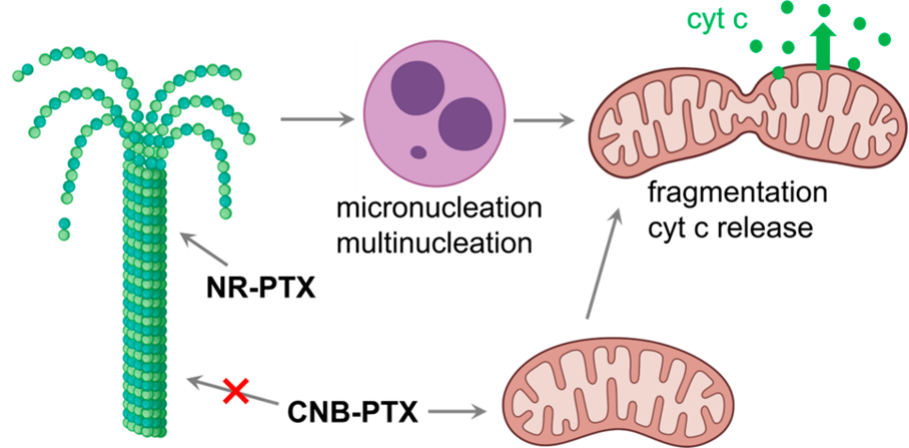
Cell death pathways triggered by taxane derivatives.

These results provide valuable insights into the different cell death mechanisms associated with taxane derivatives (Figure 4). Like paclitaxel, **NR-PTX** stabilizes microtubule dynamics, leading to prolonged mitotic block and nucleus fragmentation (mitotic catastrophe). Subsequently, mitochondrial fragmentation and cyt c release are initiated to activate the cell death program. However, **CNB-PTX** directly induces mitochondrial fragmentation and cyt c release to trigger cell death, without triggering a mitotic block or catastrophe.

Despite the clinical success of paclitaxel in treating advanced carcinoma, the efficacy of taxanes is limited by the emergence of resistance. This is primarily caused by the overexpression of multidrug resistance efflux transporters or alterations in microtubules.^54^ Certain β-tubulin isotypes (β-III and β-IV) have been implicated in resistance to taxanes in various cancer cells and have been used as improved predictive biomarkers for patients undergoing taxane-based chemotherapy.^54,55^ Our experiments on delivering paclitaxel directly into mitochondria to trigger cell death, independent of microtubule binding, could provide valuable insights for anticancer drug research. In addition, our strategy offers an appealing approach to selectively kill cancer cells, as cationic Nile blue-linked taxanes can accumulate and be retained by the mitochondria of cancer cells to a greater extent than by normal epithelial cells due to more negative ΔΨ_m_ of cancer cells.^56^

## Conclusion

In conclusion, we have successfully developed a novel class of fluorophores, cationic Nile blue, for mitochondrial imaging and targeting. These dyes possess high cell permeability, excellent mitochondrial specificity, NIR emission, solvatochromism, and good stability toward ROS. These attributes make them valuable additions to the existing repertoire of dyes. The probes can be used at low dosages for imaging mitochondria with an excellent signal-to-noise ratio in both live cells and live worms. The preparation procedure for **CNB** is concise, allowing for the rapid synthesis of analogs for future development. Moreover, the reversible redox-switching behavior of **CNB-Cl** enables SMLM imaging of mitochondria, as well as the visualization of their fusion–fission dynamics with subdiffraction-limit resolution under mild conditions. Finally, we have extended the application of cationic Nile blue to the mitochondria-specific delivery of taxanes, triggering cell death. We believe that these novel cationic Nile blue probes, with their promising properties, can enhance the toolbox of super-resolution imaging techniques and facilitate discoveries in mitochondrial biology and drug resistance mechanisms.

## Supporting Information Available

Experimental procedures, compound characterization data, and bioassay information (PDF)
Visual demonstration of the reduction and oxidation of cationic Nile blue dye (MP4)
Photoblinking dynamics of cationic Nile blue (AVI)
Application of cationic Nile blue in live-cell SMLM imaging (AVI)
Application of cationic Nile blue in live-cell SMLM imaging of mitochondria dynamics (AVI)
Transparent Peer Review report available (PDF)

## Supplementary Material

oc4c00073_si_002.mp4

oc4c00073_si_003.avi

oc4c00073_si_004.avi

oc4c00073_si_005.avi
