# Supplementary material for: Development and Application of Cationic Nile Blue Probes in Live-Cell Super-Resolution Imaging and Specific Targeting to Mitochondria
Source: ACS Cent Sci. 2024 May 17;10(6):1221–30. doi: 10.1021/acscentsci.4c00073 (PMC11212141; doi:10.1021/acscentsci.4c00073)
Supplement: Supplementary file 1 — oc4c00073_si_001.pdf [file oc4c00073_si_001.pdf]

Supporting Information for

Development and application of cationic Nile blue probes in live-cell super-resolution imaging and specific targeting to mitochondria

Yunsheng Li,[a,b] Xiaoyu Bai,[b] Dan Yang*[a,c]

[a] School of Life Sciences, Westlake University, Hangzhou 310024, China.

[b] Morningside Laboratory for Chemical Biology, Department of Chemistry, The University of Hong Kong, Hong Kong 999077, China.

[c] Westlake Laboratory of Life Sciences and Biomedicine, Hangzhou 310024, China.

* Corresponding author email: yangdan@westlake.edu.cn

Contents

1. General Methods for Synthesis 1

2. Trials on the preparation of *N, N'*-tetrasubstituted Nile blue 2

3. Synthesis of cationic Nile blue derivatives: **CNB**, **CNB-Cl** and **CNB-CO_2H** 5

4. Synthesis of fluorescent taxane derivatives: **CNB-PTX** and **NR-PTX** 9

5. Procedures for biological and spectroscopic experiments 11

6. Supplementary Figures 13

7. Spectra 26

8. References 39

1. General Methods for Synthesis

All reagents and solvents were used as received from commercial sources (Sigma-Aldrich, Acros Organics, J&K Scientific, Bide Pharm and Energy-Chemical) unless otherwise specified. Anhydrous tetrahydrofuran (THF), diethyl ether (Et_2O) and dichloromethane (DCM) were collected from a PureSolv MD Solvent Purification System made by Innovative Technology and stored in 4Å molecular sieves. Column chromatography was carried out on silica gel 60 (particle size of 0.040-0.063 mm, from various commercial sources) and eluted with solvents specified. Preparative HPLC separations were performed with Waters HPLC system equipped with photodiode array detector using XBridge Prep C18 10 µm OBD column (10 µm, 300 Å, 30 × 250 mm) at a flow rate of 15 mL/min. Mobile phases of HPLC used are as follows, Solvent A: acetonitrile; Solvent B: 0.1% TFA (v/v) in water. Nuclear magnetic resonance (NMR) spectra were recorded on Bruker Avance Fourier Transform Spectrometers at room temperature. NMR peaks were reported in δ ppm, after calibration using appropriate NMR

solvent peaks for ^1H and ^{13}C NMR, or the tetramethylsilane (TMS) peak for ^1H NMR. High-resolution mass spectra were acquired using a Thermo Scientific Electron Finnigan Gas Chromatography High Resolution MS System (for EI), and Bruker Impact II/Maxis II UHR-TOF LC-MS Systems (for ESI).

2. Trials on the preparation of *N, N'*-tetrasubstituted Nile blue

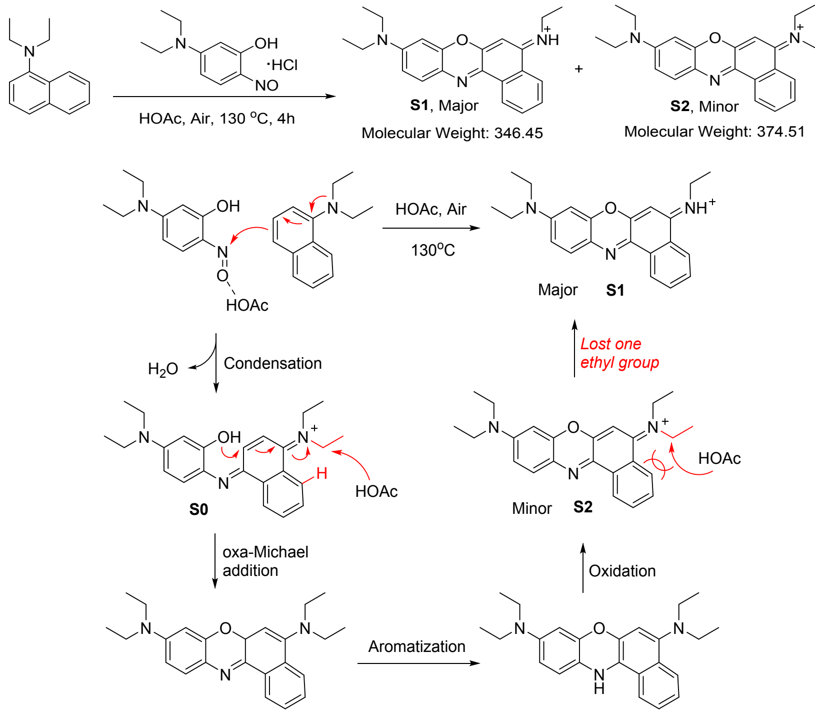

Scheme S1. Attempt to prepare tetraethyl Nile blue and proposed mechanism for the formation of of *N*, *N*'-tetraethyl Nile blue. Briefly, diethylamino naphthalene and nitroso compound were heated to 130 °C in acetic acid under air for 4 hours. Afterwards, an aliquot of reaction mixture was subjected to LC/MS analysis. Major products were isolated by column chromatography on silica gel.

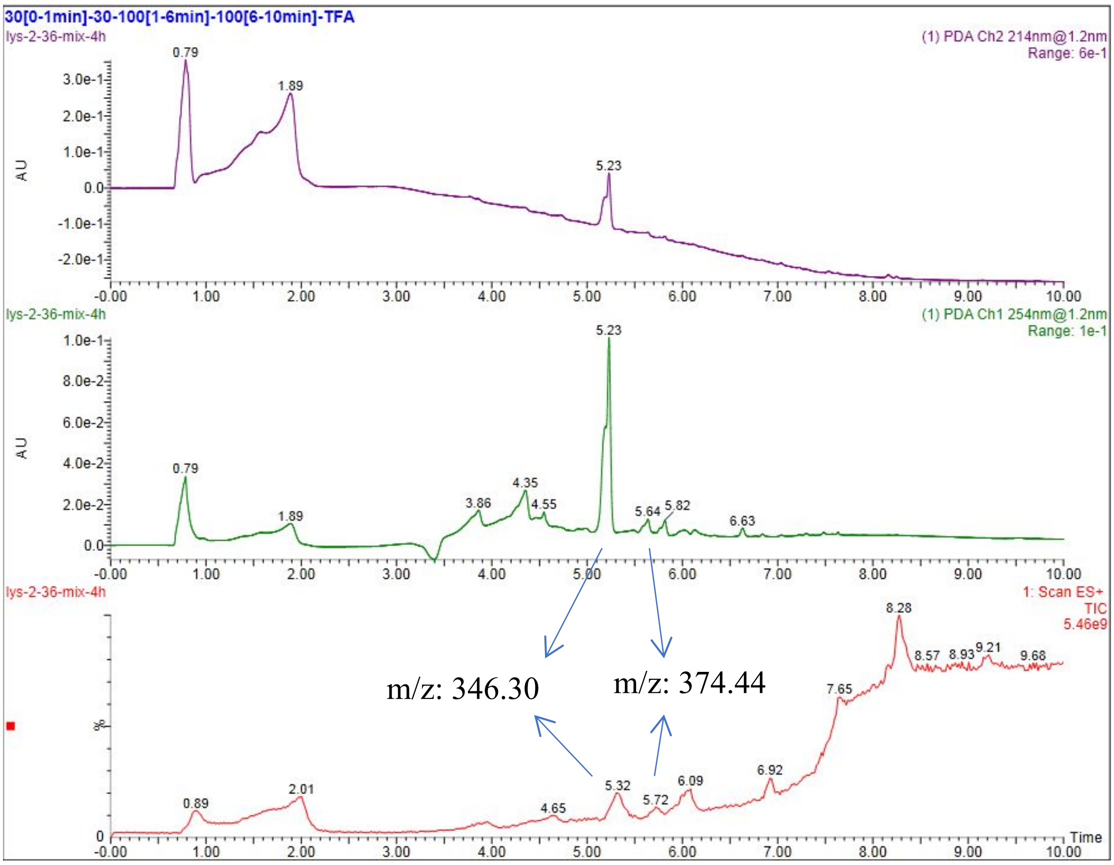

Figure S1. LC/MS analysis of the reaction components in Scheme S1. It shows that the major product (retention time of 5.23 min) has a m/z of 346.30, corresponding to triethyl Nile blue **S1**.

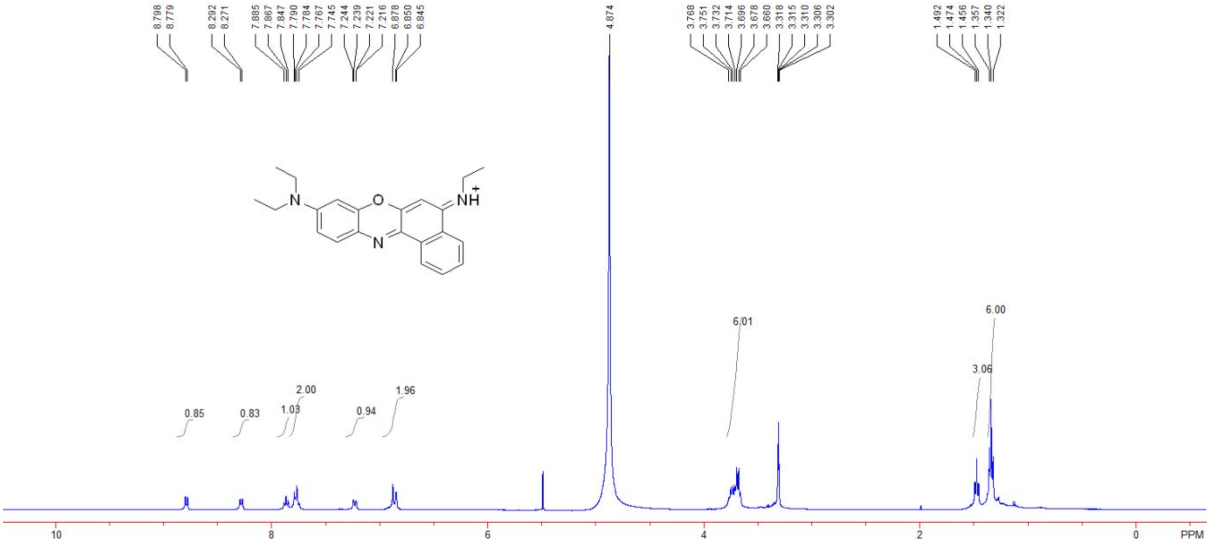

Figure S2. ^{1}H NMR spectrum of **S1**, which was isolated from the reaction shown in Scheme S1, while tetraethyl Nile blue **S2** could not be isolated due to its low yield.

In our pursuit of Nile blue derivatives as mitochondrial targeting probes, we initially focused on preparation of *N*, *N*'-tetraethyl Nile blue (compound **S2**, Scheme S1). However, only triethyl Nile blue **S1** was isolated as predominant product, whose structure was unambiguously confirmed by LC/MS and NMR spectrum (Figures S1–S2). According to the reaction outcome, a possible pathway for the condensation and formation of **S1** blue was proposed (Scheme S1). In the first step, nucleophilic addition of amino naphthalene to nitroso group occurred under the catalysis of acetic acid. Simultaneously, C−N single bond in amino naphthalene was converted to double bond (C=N) and hybridization of this nitrogen was converted from sp^3 to sp^2, by which the two ethyl groups on this nitrogen were placed on the same plane with naphthalene ring. In addition, the bond length was greatly shortened during transformation from C−N to C=N. These two factors resulted in the formation of an unstable iminium cation (showing in intermediate **S0**) and this instability mainly arose from steric hindrance experienced between one of the ethyl groups on iminium and the naphthalene ring (highlighted in red in **S0**). Therefore, it made this ethyl group easily attacked by the solvent acetic acid at elevated temperatures. A lower temperature (~100 °C) was unable to drive the reaction to happen, probably due to the instability of **S0** (high activation energy for this step).

Since steric hindrance played a deterministic role in the reaction outcome, we next replaced the two ethyl groups on the amino naphthalene with two methyl groups considering the relatively small space occupied (Scheme S2). As could be predicted from its LC/MS profile (Figure S3), the steric hindrance was greatly alleviated after carrying out such replacement. Therefore, dimethyl Nile blue **S4**, instead of monomethyl one **S3**, was generated as the major product (Scheme S2). However, we envisioned that the former product could still suffer from nucleophilic attack in live cells due to the existence of large amounts of thiol and amine-containing species, which complicates its application.

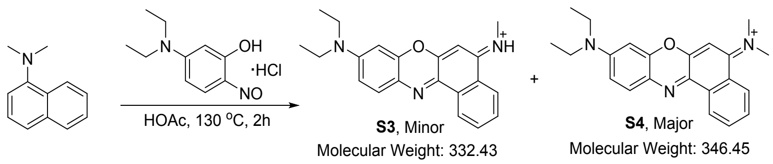

Scheme S2. Attempts to prepare dimethyl Nile blue **S4**. Briefly, dimethylamino naphthalene and nitroso compound were heated to 130 °C in acetic acid under air for 2 hours. Afterwards, an aliquot of the reaction mixture was subjected to LC/MS analysis.

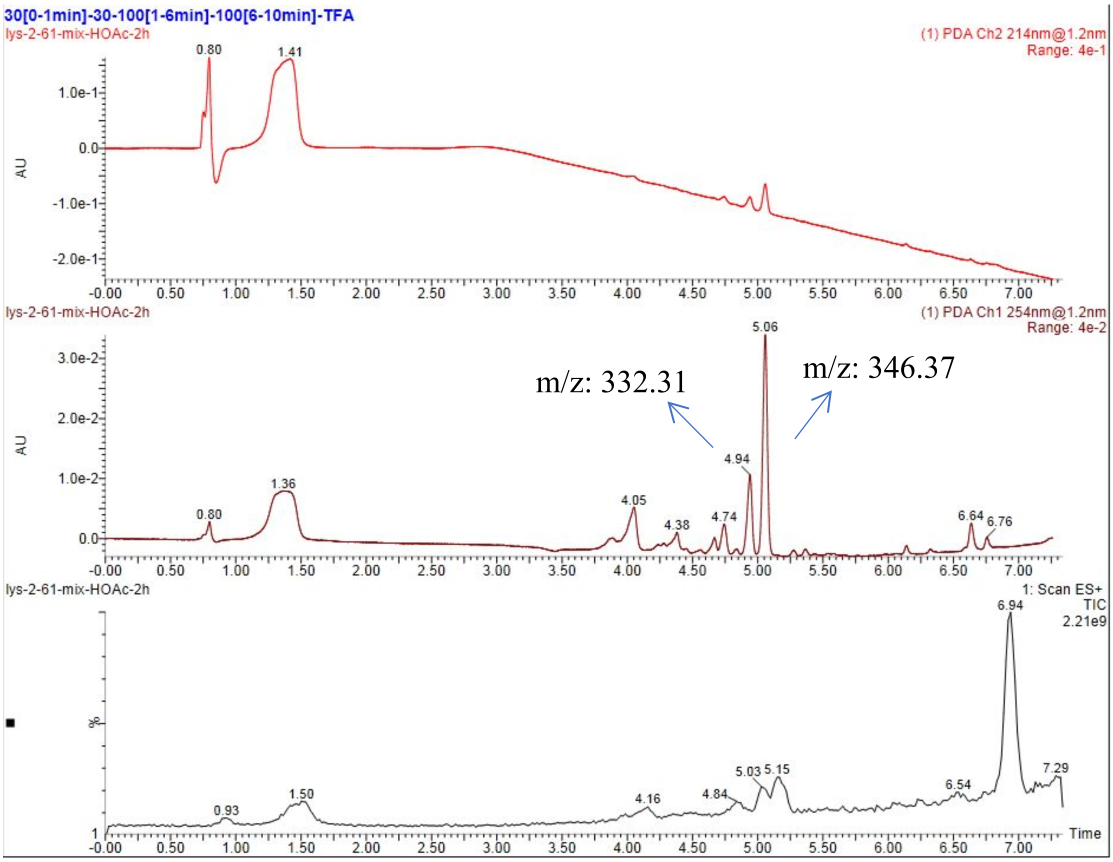

Figure S3. LC/MS analysis of reaction components in Scheme S2. It shows that the major product has a *m/z* of 346, corresponding to the desired major product **S4** in Scheme S2.

3. Synthesis of cationic Nile blue derivatives: **CNB**, **CNB-Cl** and **CNB-CO$_2$H**

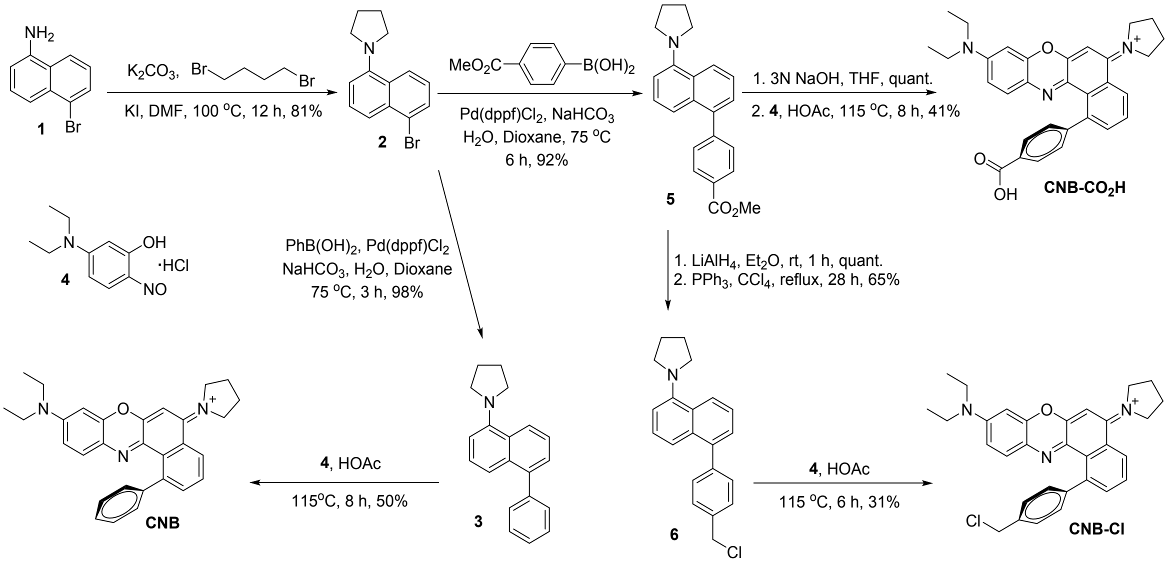

Scheme S3. Synthesis of cationic Nile blue derivatives

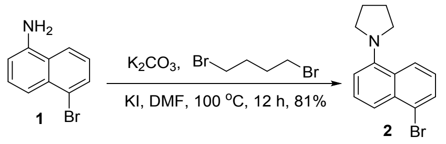

2: A mixture of 1-amino-5-bromonaphthalene (**1**, 2.22 g, 10 mmol, 1 equiv.), K_2CO_3 (5.53 g, 40 mmol, 4 equiv.) and KI (332 mg, 2 mmol, 0.2 equiv.) in anhydrous DMF (25 mL) was bubbled with argon for 5 min before the addition of 1,4-dibromobutane (2.38 mL, 20 mmol, 2 equiv.). The resulting mixture was heated to 100 °C with stirring under argon for 12 hours before cooled down to room temperature and diluted with H_2O (250 mL). The mixture was extracted with EtOAc (50 mL × 3). The combined organic phase was washed with sat. NaCl, dried over Na_2SO_4, concentrated, and purified with column chromatography on silica gel (eluent: Hexane to Hexane/EtOAc = 100/1) to give **2** as a brown oil (2.24 g, 81%).

^{1}H NMR (400 MHz, $CDCl_3$) δ 8.26 (d, *J* = 8.6 Hz, 1H), 7.94 (d, *J* = 8.4 Hz, 1H), 7.81 (d, *J* = 7.2 Hz, 1H), 7.52 (d, *J* = 8.2, 1H), 7.30 (t, *J* = 7.9 Hz, 1H), 7.07 (d, *J* = 7.6 Hz, 1H), 3.44 – 3.28 (m, 4H), 2.12 – 1.94 (m, 4H); ^{13}C NMR (101 MHz, $CDCl_3$) δ 148.2, 133.5, 129.9, 129.6, 127.4, 124.9, 124.4, 123.2, 120.3, 112.5, 53.0, 24.9; LRMS (ESI) for $C_{14}H_{15}BrN$ ([M+H]$^+$): Calcd. 276.04, 278.04, found 276.32, 278.35; HRMS (ESI) for $C_{14}H_{15}BrN$ ([M+H]$^+$): Calcd. 276.0388, 278.0367, found 276.0378, 278.0358.

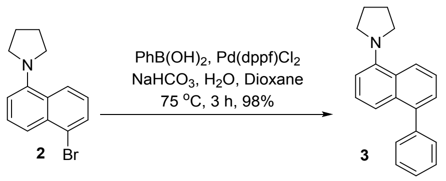

3: The mixture of **2** (192 mg, 0.695 mmol, 1 equiv.), phenylboronic acid (93 mg, 0.765 mmol, 1.1 equiv.), Pd(dppf)Cl_2 (25 mg, 0.034 mmol, 0.05 equiv.) and $NaHCO_3$ (117 mg, 1.39 mmol, 2 equiv.) in Dioxane (4 mL) and H_2O (1mL) was bubbled with argon for 5 min before heated to 75 °C with stirring under argon. Three hours later, the mixture was cooled down to room temperature and passed through a pad of silica gel, washed with EtOAc, and concentrated to dryness. The residue was purified with column chromatography on silica gel (eluent: Hexane/EtOAc = 100/1) to give **3** as a brown oil (187 mg, 98%).

^{1}H NMR (400 MHz, $CDCl_3$) δ 8.32 (d, *J* = 8.5 Hz, 1H), 7.59 – 7.50 (m, 6H), 7.49 – 7.42 (m, 2H), 7.36 (t, *J* = 8.0 Hz, 1H), 7.06 (d, *J* = 7.5 Hz, 1H), 3.48 – 3.37 (m, 4H), 2.14 – 2.04 (m, 4H); ^{13}C NMR (101 MHz, $CDCl_3$) δ 148.1, 141.6, 140.5, 133.2, 130.3, 128.9, 128.2, 127.1, 126.8, 126.0, 124.5, 123.9, 119.7, 111.7, 53.0, 24.8; LRMS (ESI) for $C_{20}H_{20}N$ ([M+H]$^+$): Calcd. 274.16, found 274.51. HRMS (ESI) for $C_{20}H_{20}N$ ([M+H]$^+$): Calcd. 274.1596, found 274.1590.

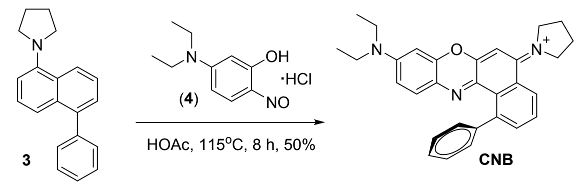

CNB: The stirred mixture of **3** (27 mg, 0.1 mmol, 1 equiv.) and **4**[1] (23 mg, 0.1 mmol, 1equiv.) in HOAc (2 mL) was heated to 115 °C under air. Four hours later, another portion of **4** (23 mg, 0.1 mmol, 1 equiv.) was added, and the reaction was heated for additional 4 hours before cooled down to room temperature. The mixture was concentrated and purified with column chromatography on silica gel (eluent: DCM/MeOH = 30/1 to 15/1) to give a dark blue solid, which may be purified by prep-HPLC on C18 column when necessary (eluent: ACN/H_2O (containing 0.1% TFA) = 70/30) to give a dark blue solid **CNB** as a TFA salt (28 mg, 50%).

^1H NMR (500 MHz, CD_3OD) δ 8.40 (d, J = 8.2 Hz, 1H), 7.75 (t, J = 7.8 Hz, 1H), 7.66 (d, J = 7.2 Hz, 1H), 7.45 – 7.37 (m, 3H), 7.28 – 7.21 (m, 2H), 6.95 (dd, J = 9.4, 2.7 Hz, 1H), 6.93 (s, 1H), 6.74 – 6.66 (m, 2H), 4.17 – 4.04 (broad s, 4H), 3.59 (q, J = 7.1 Hz, 4H), 2.23 – 2.12 (broad s, 4H), 1.26 (t, J = 7.1 Hz, 6H); ^{13}C NMR (126 MHz, CD_3OD) δ 160.9, 155.3, 151.8, 148.4, 145.6, 143.8, 136.2, 136.0, 133.8, 131.2, 129.6, 128.96, 128.94, 128.7, 128.5, 127.61, 127.58, 115.5, 98.7, 96.5, 56.6, 46.8, 26.6, 12.8; ^{19}F NMR (471 MHz, CD_3OD) δ –77.2; LRMS (ESI) for $C_{30}H_{30}N_3O$ (M^+): Calcd. 448.24, found 448.55; HRMS (ESI) for $C_{30}H_{30}N_3O$ (M^+): Calcd. 448.2383, found 448.2386.

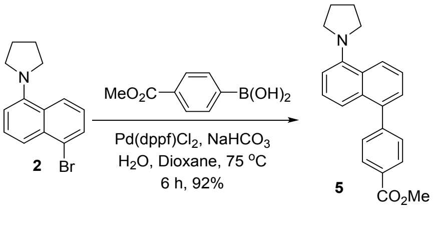

5: The mixture of **2** (1.15 g, 4.04 mmol, 1 equiv.), 4-methoxycarbonylphenylboronic acid (763 mg, 4.24 mmol, 1.05 equiv.), Pd(dppf)Cl_2 (147 mg, 0.2 mmol, 0.05 equiv.) and $NaHCO_3$ (679 mg, 8.08 mmol, 2 equiv.) in dioxane (8 mL) and H_2O (2 mL) was bubbled with argon for 5 min before heated to 75 °C with stirring under argon. Six hours later, the mixture was cooled down to room temperature and passed through a pad of silica gel, washed with EtOAc, and concentrated to dryness. The residue was purified with column chromatography on silica gel (eluent: Hexane/EtOAc = 100/1 to 40/1) to give **5** as a brown oil (1.23 g, 92%).

^1H NMR (400 MHz, $CDCl_3$) δ 8.37 (d, J = 8.6 Hz, 1H), 8.24 (d, J = 8.2 Hz, 2H), 7.63 (d, J = 8.1 Hz, 2H), 7.55 – 7.46 (m, 2H), 7.41 (d, J = 6.9 Hz, 1H), 7.35 (t, J = 8.0 Hz, 1H), 7.04 (d, J = 7.5 Hz, 1H), 4.02 (s, 3H), 3.47 – 3.31 (m, 4H), 2.13 – 1.96 (m, 4H); ^{13}C NMR (101 MHz, $CDCl_3$) δ 166.8, 148.0, 146.2, 139.1, 132.6, 130.1, 129.4, 128.7, 128.6, 126.6, 126.2, 124.9, 123.5, 118.8, 111.6, 52.7, 51.9, 24.6; LRMS (ESI) for $C_{22}H_{22}NO_2$ ($[M+H]^+$): Calcd. 332.17, found 332.52; HRMS (ESI) for $C_{22}H_{22}NO_2$ ($[M+H]^+$): Calcd. 332.1651, found 332.1649.

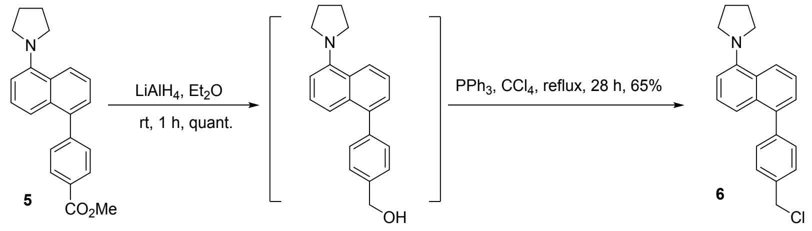

6: To a stirred solution of **5** (331 mg, 1 mmol, 1 equiv.) in anhydrous Et_2O (10 mL) was added $LiAlH_4$ (38 mg, 1 mmol, 1 equiv.) slowly at room temperature. The resulting mixture was stirred for 1 h under argon before quenched with H_2O (40 μL), 15% NaOH (80 μL) and H_2O (0.12 mL) successively. After dried over Na_2SO_4, the mixture was filtrated through a pad of celite, washed with EtOAc and concentrated to give a pale-yellow oil, which was dissolved in CCl_4 (10 mL) and followed by addition of PPh_3 (262 mg, 1 mmol). The resulting mixture was heated to reflux under argon for 28 hours before cooled down to room temperature, concentrated and purified with column chromatography on silica gel (eluent: Hexane to Hexane/EtOAc = 50/1) to give **6** as a pale-yellow oil (210 mg, 65%).

^1H NMR (400 MHz, $CDCl_3$) δ 8.29 (d, J = 8.6 Hz, 1H), 7.56 – 7.44 (m, 6H), 7.40 (dd, J = 7.0, 1.3 Hz, 1H), 7.33 (t, J = 8.0 Hz, 1H), 7.04 (d, J = 7.5 Hz, 1H), 4.71 (s, 2H), 3.46 – 3.31 (m, 4H), 2.12 – 2.00 (m, 4H); ^{13}C NMR (101 MHz, $CDCl_3$) δ 148.2, 141.8, 139.8, 136.3, 133.1, 130.6, 128.8, 128.5, 126.8, 126.1, 124.7, 123.9, 119.4, 111.8, 53.0, 46.3, 24.8; LRMS (ESI) for

$C_{21}H_{21}ClN$ ([M+H]$^+$): Calcd. 322.14, 324.13, found 322.44, 324.47; HRMS (ESI) for $C_{21}H_{21}ClN$ ([M+H]$^+$): Calcd. 322.1363, 324.1333, found 322.1357, 324.1335.

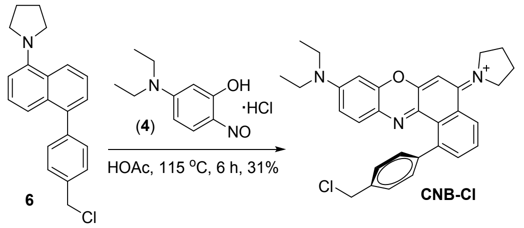

CNB-Cl: The mixture of **6** (32 mg, 0.1 mmol, 1 equiv.) and **4** (23 mg, 0.1mmol, 1 equiv.) in HOAc (3 mL) was heated to 115 °C under air. Three hours later, another portion of **4** (23 mg, 0.1 mmol, 1 equiv.) was added, and the reaction was heated for additional 3 h before cooled down to room temperature. The mixture was concentrated and purified with column chromatography on silica gel (eluent: DCM/MeOH = 30/1 to 12/) to give a dark blue solid, which was further purified by prep-HPLC on C18 column (eluent: ACN/H_2O (containing 0.1% TFA) = 70/30) to give a dark blue solid **CNB-Cl** as a TFA salt (19 mg, 31%).

^{1}H NMR (500 MHz, CD_3OD) δ 8.47 (dd, *J* = 8.4, 1.3 Hz, 1H), 7.80 (dd, *J* = 8.5, 7.5 Hz, 1H), 7.71 (dd, *J* = 7.5, 1.0 Hz, 1H), 7.51 – 7.45 (m, 2H), 7.30 – 7.25 (m, 2H), 7.03 (s, 1H), 6.97 (dd, *J* = 9.4, 2.7 Hz, 1H), 6.83 (d, *J* = 9.5 Hz, 1H), 6.78 (d, *J* = 2.5 Hz, 1H), 4.80 (s, 2H), 4.23 – 4.10 (m, 4H), 3.62 (q, *J* = 7.1 Hz, 4H), 2.26 – 2.12 (m, 4H), 1.26 (t, *J* = 7.2 Hz, 6H); ^{13}C NMR (126 MHz, CD_3OD) δ 161.1, 155.4, 152.1, 148.6, 145.8, 143.2, 138.1, 136.08, 136.06, 133.9, 131.3, 130.0, 129.5, 129.1, 128.9, 128.6, 127.7, 115.4, 98.8, 96.5, 56.5, 47.2, 46.8, 26.7, 12.8; ^{19}F NMR (376 MHz, CD_3OD) δ –77.3; LRMS (ESI) for $C_{31}H_{31}ClN_3O$ (M$^+$): Calcd. 496.22, found 496.26; HRMS (ESI) for $C_{31}H_{31}ClN_3O$ (M$^+$): Calcd. 496.2150, found 496.2165.

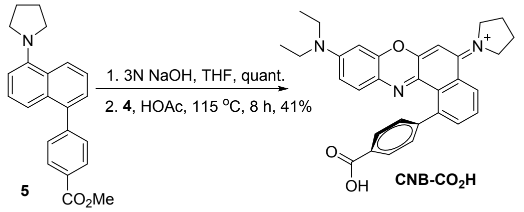

CNB-CO$_2$H: The solution of **5** (193 mg, 0.582 mmol, 1 equiv.) in THF (6 mL) and 6 N NaOH (1 mL) was heated to reflux for 18 h. The mixture was cooled down to room temperature before THF was remove by rota vapor. The residue was diluted with 3 mL of H_2O and adjusted to pH 6 with 3 N HCl. Yellow precipitate was formed and collected by filtration, washed with H_2O, and dried on high vacuum (181 mg, 98%). A mixture of this yellow solid (32 mg, 0.1 mmol, 1 equiv.) and **4** (23 mg, 0.1 mmol, 1 equiv.) in HOAc (2 mL) was heated to 115 °C under air. Four hours later, another portion of **4** (23 mg, 0.1 mmol, 1 equiv.) was added, and the reaction was heated for additional 4 hours before cooled down to room temperature. The mixture was concentrated and purified with column chromatography on silica gel (eluent: DCM/MeOH = 50/1 to 10/1, containing 1% HOAc) to give a dark blue solid. It may be further purified by prep-HPLC on C18 column when necessary (eluent: ACN/H_2O (containing 0.1% TFA) = 47/53) to give a dark blue solid **CNB-CO$_2$H** as a TFA salt (25 mg, 41%).

^{1}H NMR (400 MHz, CD_3OD) δ 8.43 (d, *J* = 8.3 Hz, 1H), 8.03 (d, *J* = 7.8 Hz, 2H), 7.77 (t, *J* = 7.9 Hz, 1H), 7.63 (d, *J* = 7.2 Hz, 1H), 7.32 (d, *J* = 8.0 Hz, 2H), 6.97 – 6.85 (m, 2H), 6.69 (s, 1H), 6.57 (d, *J* = 9.2 Hz, 1H), 4.18 – 4.04 (broad s, 4H), 3.58 (q, *J* = 7.1 Hz, 4H), 2.25 – 2.10 (broad s, 4H), 1.25 (t, *J* = 7.0 Hz, 6H); ^{13}C NMR (126 MHz, CD_3OD) δ 169.8, 160.6, 155.4, 151.7, 150.5, 148.5, 142.4, 135.8, 135.5, 133.4, 130.9, 130.4, 130.1, 129.8, 129.2, 129.1, 128.6, 127.5, 115.8, 98.8, 96.6, 56.6, 46.9, 26.7, 12.8; ^{19}F NMR (376 MHz, CD_3OD) δ –77.2; LRMS

(ESI) for $C_{31}H_{30}N_3O_3$ ($[M]^+$): Calcd. 492.23, found 492.34; HRMS (ESI) for $C_{31}H_{30}N_3O_3$ ($[M]^+$): Calcd. 492.2282, found 492.2290.

4. Synthesis of fluorescent taxane derivatives: **CNB-PTX** and **NR-PTX**

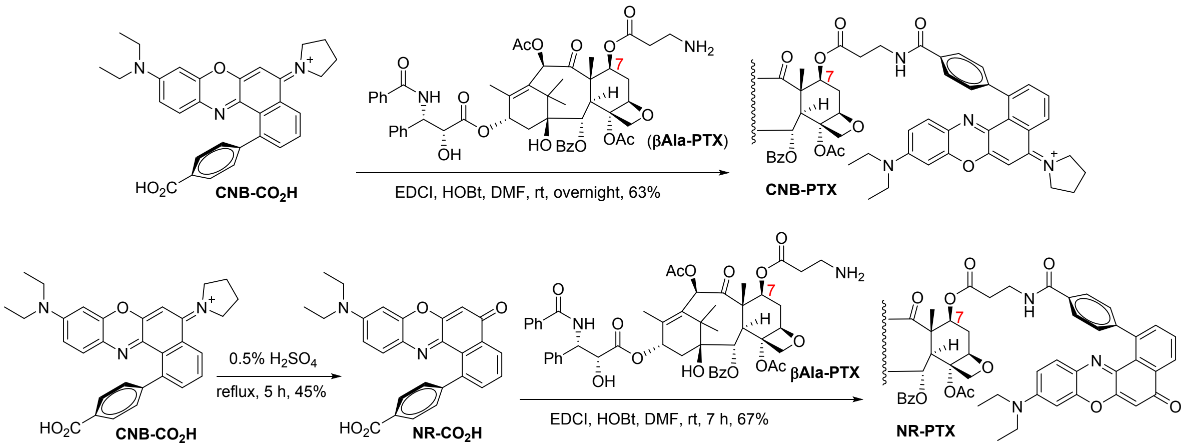

Scheme S4. Synthesis of taxane derivatives: **CNB-PTX** and **NR-PTX**.

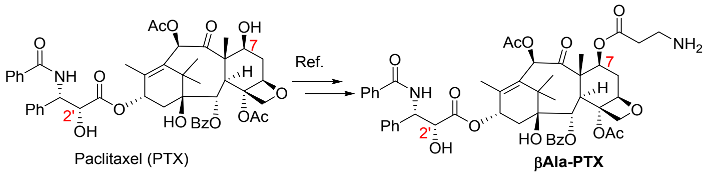

βAla-PTX: prepared from paclitaxel according to the reference[2]. Its ^1H NMR spectrum is identical with the reported one. HRMS (ESI) for $C_{50}H_{57}N_2O_{15}$ ($[M+H]^+$): Calcd. 925.3759, found 925.3768.

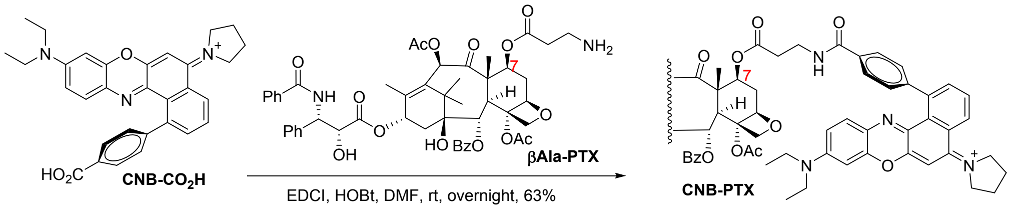

CNB-PTX: The mixture of **βAla-PTX** (19 mg, 0.02 mmol, 1 equiv.), **CNB-CO₂H** (12 mg, 0.02 mmol, 1 equiv.), EDCI (12 mg, 0.06 mmol, 3 equiv.) and HOBt (6 mg, 0.04 mmol, 2 equiv.) in anhydrous DMF (3 mL) was stirred at room temperature overnight. The mixture was then concentrated and purified with column chromatography on silica gel (eluent: DCM/MeOH = 20/1 to 15/1). It was further purified by prep-HPLC on C18 column (eluent: ACN/H_2O (containing 0.1% TFA) = 65/35) to give a dark blue solid **CNB-PTX** as a TFA salt (19 mg, 63%).

^1H NMR (500 MHz, CD_3OD) δ 8.50 (d, J = 8.3 Hz, 1H), 8.15 – 8.07 (m, 2H), 7.94 – 7.77 (m, 5H), 7.74 – 7.64 (m, 2H), 7.62 – 7.34 (m, 11H), 7.29 (t, J = 7.4 Hz, 1H), 7.05 (s, 1H), 7.00 (dd, J = 9.5, 2.7 Hz, 1H), 6.81 – 6.71 (m, 2H), 6.28 (s, 1H), 6.15 (t, J = 9.0 Hz, 1H), 5.69 – 5.60 (m, 3H), 4.98 (d, J = 9.3 Hz, 1H), 4.74 (d, J = 5.3 Hz, 1H), 4.25 – 4.11 (m, 6H), 3.92 (d, J = 7.0 Hz, 1H), 3.79 – 3.64 (m, 2H), 3.60 (q, J = 7.1 Hz, 4H), 2.74 – 2.64 (m, 2H), 2.63 – 2.53 (m, 1H), 2.38 (s, 3H), 2.30 – 2.17 (m, 5H), 2.11 (s, 3H), 2.07 – 1.96 (m, 1H), 1.87 (s, 3H), 1.86 –

1.80 (m, 1H), 1.79 (s, 3H), 1.25 (t, *J* = 7.1 Hz, 6H), 1.16 (s, 3H), 1.11 (s, 3H); ^{13}C NMR (126 MHz, CD_3OD) δ 203.8, 174.5, 172.5, 172.1, 171.2, 170.3, 170.1, 167.6, 161.0, 155.4, 152.2, 149.3, 148.7, 142.6, 142.2, 140.0, 135.94, 135.91, 135.6, 134.7, 134.3, 133.8, 133.7, 132.9, 131.3, 131.2, 129.9, 129.8, 129.6, 129.2, 129.1, 129.0, 128.6, 128.5, 128.0, 127.7, 115.8, 98.8, 96.5, 85.1, 81.9, 78.9, 77.3, 76.8, 75.8, 74.9, 73.2, 72.2, 57.7, 57.3, 56.6, 49.6, 48.0, 46.8, 44.6, 36.7, 36.5, 34.9, 34.3, 26.8, 26.7, 23.2, 22.1, 20.7, 14.8, 12.8, 11.5; ^{19}F NMR (376 MHz, CD_3OD) δ –77.2; LRMS (ESI) for $C_{81}H_{84}N_5O_{17}$ (M^+): Calcd. 1398.59, found 1399.09; HRMS (ESI) for $C_{81}H_{84}N_5O_{17}$ (M^+): Calcd. 1398.5857, found 1398.5896.

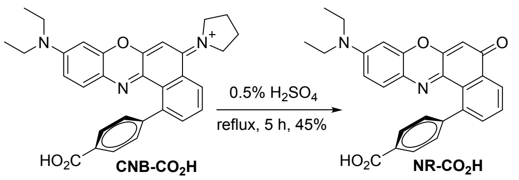

NR-CO₂H: The mixture of **CNB-CO₂H** (60 mg, 0.1 mmol) in 0.5% H_2SO_4 (2 mL) was heated to 110 °C with stirring for 5 hours before cooled down to room temperature. The mixture was then concentrated and purified with column chromatography on silica gel (eluent: DCM/MeOH = 30/1, containing 1% HOAc) to give **NR-CO₂H** as a black solid (20 mg, 45%) and the recovered starting material (30 mg, 50%).

^1H NMR (500 MHz, $CDCl_3/CD_3OD$ ~ 1/1, v/v) δ 8.37 (dd, *J* = 7.9, 1.5 Hz, 1H), 8.05 (d, *J* = 8.2 Hz, 2H), 7.66 (t, *J* = 7.7 Hz, 1H), 7.54 (dd, *J* = 7.4, 1.5 Hz, 1H), 7.34 (d, *J* = 8.2 Hz, 2H), 6.62 – 6.53 (m, 2H), 6.45 (d, *J* = 2.4 Hz, 1H), 6.39 (s, 1H), 3.45 (q, *J* = 7.1 Hz, 4H), 1.20 (t, *J* = 7.1 Hz, 6H); ^{13}C NMR (125 MHz, $CDCl_3/CD_3OD$ ~ 1/1) δ 184.6, 170.1, 153.5, 152.2, 150.1, 146.6, 141.2, 138.4, 135.4, 133.3, 132.0, 129.9, 129.7, 129.25, 129.21, 126.40, 126.37, 124.9, 111.2, 104.9, 96.2, 45.7, 12.7; LRMS (ESI) for $C_{27}H_{23}N_2O_4$ ($[M+H]^+$): Calcd. 439.17, found 439.55; HRMS (ESI) for $C_{27}H_{23}N_2O_4$ ($[M+H]^+$): Calcd. 439.1658, found 439.1655.

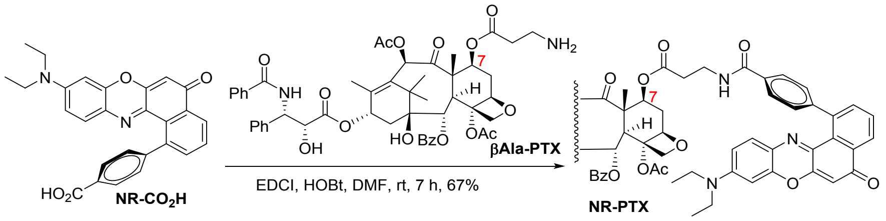

NR-PTX: The mixture of **βAla-PTX** (9 mg, 0.01 mmol, 1 equiv.), **NR-CO₂H** (4 mg, 0.01 mmol, 1 equiv.), EDCI (6 mg, 0.03 mmol, 3 equiv.) and HOBt (3 mg, 0.02 mmol, 2 equiv.) in anhydrous DMF (2 mL) was stirred at room temperature for 7 hours. The mixture was then concentrated and purified with column chromatography on silica gel (eluent: DCM/MeOH = 100/1 to 30/1) to give **NR-PTX** as a red solid (9 mg, 67%).

^1H NMR (400 MHz, $CDCl_3$) δ 8.44 (d, *J* = 7.9 Hz, 1H), 8.11 (d, *J* = 7.7 Hz, 2H), 7.87 (d, *J* = 8.0 Hz, 2H), 7.74 (d, *J* = 7.4 Hz, 2H), 7.67 – 7.58 (m, 2H), 7.55 – 7.27 (m, 14H), 7.08 (d, *J* = 8.9 Hz, 1H), 6.61 (d, *J* = 9.0 Hz, 1H), 6.48 (dd, *J* = 9.1, 2.7 Hz, 1H), 6.40 (s, 1H), 6.36 (d, *J* = 2.6 Hz, 1H), 6.29 (s, 1H), 6.18 (t, *J* = 8.8 Hz, 1H), 5.79 (dd, *J* = 8.9, 2.5 Hz, 1H), 5.73 – 5.58 (m, 2H), 4.93 (d, *J* = 9.2 Hz, 1H), 4.82 – 4.76 (m, 1H), 4.32 (d, *J* = 8.5 Hz, 1H), 4.19 (d, *J* = 8.5 Hz, 1H), 3.94 (d, *J* = 6.8 Hz, 1H), 3.88 – 3.68 (m, 3H), 3.40 (q, *J* = 7.1 Hz, 4H), 2.70 – 2.54 (m, 3H), 2.39 (s, 3H), 2.34 (d, *J* = 9.0 Hz, 2H), 2.04 (s, 3H), 1.92 – 1.79 (m, 8H), 1.23 – 1.17 (m, 9H), 1.15 (s, 3H). ^{13}C NMR (100 MHz, $CDCl_3$) δ 202.2, 183.4, 172.8, 171.8, 170.6, 169.9, 167.75, 167.68, 167.2, 167.0, 152.5, 151.1, 148.2, 146.0, 140.8, 140.6, 139.2, 138.2, 134.9, 134.0, 133.8, 133.3, 133.0, 132.39, 132.37, 132.1, 131.5, 130.3, 129.4, 129.2, 129.1, 128.9, 128.8, 128.5, 127.20, 127.18, 126.6, 126.3, 123.9, 110.1, 105.4, 95.8, 83.9, 81.1, 78.6, 77.4,

76.5, 75.6, 74.4, 73.3, 72.3, 71.7, 56.3, 55.1, 47.0, 45.3, 43.4, 35.7, 33.9, 33.7, 26.6, 22.7, 21.1, 20.9, 14.8, 12.7, 11.0.

LRMS (ESI) for $C_{77}H_{77}N_4O_{18}$ ($[M+H]^+$): Calcd. 1345.52, found 1345.65. HRMS (ESI) for $C_{77}H_{77}N_4O_{18}$ ($[M+H]^+$): Calcd. 1345.5233, found 1345.5227.

5. Procedures for biological and spectroscopic experiments

Cell culture

HeLa cells or COS-7 cells were cultured as sub-confluent monolayers on 100 mm cell culture dishes in complete growth medium, *i.e.* Dulbecco's Modified Eagle Medium (DMEM) supplemented with heat inactivated Fetal Bovine Serum (FBS, 10%, v/v) and Penicillin-Streptomycin (PS, 1%, v/v), in a humidified incubator (70 – 95 %) at 37 °C with CO_2 (5%). Cells grown to sub-confluence were enzymatically dissociated from the surface with 1 mL of a solution of trypsin (0.05%)/EDTA and washed with 2 mL of fresh medium. The cells were spun down (1000 rpm × 3 min) for counting.

U-2 OS cells were cultured under the same conditions used for HeLa culturing despite that McCoy's 5A Modified Medium supplemented with heat inactivated FBS (10%, v/v) and PS (1%, v/v) was used as complete growth medium.

Confocal imaging of live cells

The cells were plated in 35 mm glass bottom confocal dishes (Mat-Tek) at density of 1×10^5 cell/mL in 2-mL seeding volume 1 day prior to the imaging, or at density of 5×10^4 cell/mL in 2-mL seeding volume 2 days prior to the imaging. These conditions produced a monolayer at sub-confluence for the experiments. After incubation overnight, the medium was discarded and replaced with Hanks' Balanced Salt Solution (HBSS) containing probes of specified concentrations for different time. Before imaging, cells were washed with HBBS for 3 times and placed in DMEM (phenol red free). Fluorescence microscopy was performed with Zeiss LMS 780 or 880 confocal microscope with a 64× or 40×/1.3 oil-immersion objective lens. A stage-top incubator was used to maintain an imaging environment at 37 °C with 5% CO_2. Excitation wavelength: 633 nm for **CNB, CNB-Cl, CNB-PTX** and MTDR; 561 nm for MTR and **NR-PTX**; 488 nm for Cyt c monoclonal antibody-Alexa Fluor 488 conjugates, LTG and MTG; 405 nm for Hoechst.

Mapping the distribution of cytochrome c in probes treated cells

The cells were plated in 35 mm glass bottom confocal dishes (Mat-Tek) at density of 5×10^4 cell/mL in 2-mL seeding volume two days prior to the imaging. After overnight incubation, the medium was changed with fresh medium containing various concentrations of probes as specified in the figure descriptions containing 0.1% DMSO. After incubation for 24 hours at 37 °C in a 5% CO_2 incubator, the medium was discarded, and cells were washed with PBS twice. The cells were fixed with 4% PFA in PBS at room temperature for 15 min, washed with PBS twice, blocked with 3% BSA at room temperature for 30 min, washed with PBS twice successively. The cells were than incubated with cyt c monoclonal antibody (6H2.B4)-Alexa fluor 488 conjugates (Thermo Fisher) at a dilution of 5 μg/mL in blocking buffer for 1 hour. The cells were washed with PBS three times before mounted onto confocal microscope. Fluorescence imaging was performed with Zeiss LMS 780 or 880 confocal microscope with a 64× or 40×/1.3 oil-immersion objective lens.

Cell viability assay

The effects of different probes on cell viability were analysed using CellTiter-Glo® (Promega) Luminescent cell viability assay. Cells were seeded at density of 5000 cells/well into a 96-well microplate (black plate, clear flat bottom with lid) and incubated in DMEM containing 10% FBS and 1% PS at 37 °C in a 5% CO_2 incubator overnight. The medium was then replaced with fresh medium containing various concentrations of probes 1% DMF. After incubation for 24 h, cells were equilibrated at room temperature for approximately 30 min before loaded with 50 µL of Cell-Titer Glo reagent. The microplates were subjected to gentle shaking for cell lysis at room temperature (approximately 10 min). The microplate was mounted onto a DTX multimode plate reader (Molecular Devices) for luminescence detection (550 nm), by using cellular ATP contents as a measure of cell viability.

Evaluation of cellular uptake of cationic Nile blue derivatives

The HeLa cells were seeded in 5 cm of cultural dishes at density of 2×10^5 cell/mL in 4-mL seeding volume and allowed to grow at 37°C. 24 hours later, the medium (DMEM) was replaced with fresh medium containing various concentrations of taxane derivatives or cationic Nile blue containing 0.1% DMSO. After incubation for 6 hours, the medium was discarded, and the cells were washed with PBS twice and trypsinized. After centrifugation, the cell pellet was resuspended in PBS and recentrifuged. The pellet was then extracted with 1 ml of 95% ethanol. After sonication for 20 min and further centrifugation, the concentration of probes in supernatant was analyzed by reading fluorescence intensity at 680 nm with excitation at 645 nm.

Absorption and Fluorescence analysis

Fluorescent dyes were dissolved in DMF or DMSO to make a 10 mM stock solution, which was diluted to the required concentration of testing solution for measurement in a 1 cm × 1 cm quartz cuvette. UV-Visible absorption spectra of sample solutions in spectral grade solvents were measured using a Shimadzu UV-3150 spectrometer or an L6S Split Beam UV-VIS Spectrophotometer (INESA Analytical Instrument Co., Ltd.). Fluorescence measurements were carried out at room temperature on a Hitachi F-7000 fluorescence spectrophotometer or an FS5 Spectrofluorometer (Edinburgh Instruments Ltd.).

Fluorescence quantum yield (Q.Y.) measuring

Fluorescence quantum yields were determined at a temperature of 25 °C employing a relative method, which utilized Nile blue A (Φ_F = 0.27 in EtOH)[3] as a standard. The relative fluorescence quantum yield was ascertained using the subsequent equation:

$$\Phi_F(x) = (A_S/A_X)(F_X/F_S)(n_X/n_S)^2\Phi_F(s)$$

where A is the absorbance (below 0.1 A.U.), F is the area under the emission curve, n is the refractive index of the solvents (at 25 °C) used in the measurements, and the subscripts s and x represent standard and unknown, respectively. The refractive index values used in these measurements were 1.36 for ethanol and 1.33 for PBS.

Microscope setup for single-molecule localization microscopy

SMLM experiments were performed on a custom-built microscope (Nano BioImaging SRiS 2.0) based on a Nikon eclipse Ti-E inverted microscope. A single activation/imaging photodiode laser (647 nm, 500 mW) was focused to the back focal plane of the objective (CFI Apochromat TIRF 100× Oil N.A. 1.49). The laser intensity was controlled directly by a Rhodea 2.0 software. A dichroic beam splitter (T760LPXR-UF2) and a bandpass filter (FF01-692/40-25) separated the fluorescence emission from the excitation light. The fluorescence was

recorded with an electron-multiplying CCD camera (Andor iXon Ultra 897). 10000 – 20000 frames were recorded. During data acquisition, a Perfect Focus System was used to maintain a constant focal plane.

Sample preparation and data analysis of single-molecule localization microscopy

For fixed cells: U-2 OS cells were seeded in a 35 mm culture dish containing an 18 mm, round glass coverslip (Marienfeld) at density of 1 × 10^5 cell/mL in 2-mL seeding volume one day prior to the imaging. After overnight incubation at 37 °C in a 5% CO_2 incubator, the medium was discarded, and the cells were incubated with 200 nM of probes in HBSS for 1 hour. Cells were then washed with PBS twice and fixed with 4% PFA at room temperature for 15 min. The cells were then washed with PBS 3 times and further treated with Triton^{TM} X-100 (0.1% in PBS) for 3 min at room temperature. The resulting fixed permeabilized cells were washed 3 times with PBS before further treatment. The coverslip was lifted from culture dish and placed in imaging chamber. The cells were immersed with 380 µl of imaging buffer (containing 1mM ascorbic acid, 1 mM methyl viologen and 50 mM Tris-HCl 8.0 in MilliQ H_2O) and covered with an 18 mm × 18 mm square coverslip. The imaging chamber was then mounted onto the microscope for both wide-field and super-resolution imaging.

For live cells: U-2 OS cells, COS-7 cells or HeLa cells were seeded in a 35 mm culture dish containing an 18 mm, round glass coverslip (Marienfeld) at density of 1 × 10^5 cell/mL in 2-mL seeding volume one day prior to the imaging. After overnight incubation at 37 °C in a 5% CO_2 incubator, the medium was discarded, and the cells were incubated with 100~250 nM of probes in HBSS for 1 h. The cells were then washed with DMEM twice. The coverslip was lifted from culture dish and placed in imaging chamber. The cells were immersed with 380 µL of DMEM (phenol red free) and covered with an 18 mm × 18 mm square coverslip. The imaging chamber was then mounted onto the microscope for both wide-field and super-resolution imaging.

SMLM data were pre-processed with the HAWK plugin[4] using five levels to remove potential artifacts, followed by single-emitter fitting with ThunderSTORM plugin.[5] Fluorescent spots that were too dim or too bright were discarded.

6. Supplementary Figures

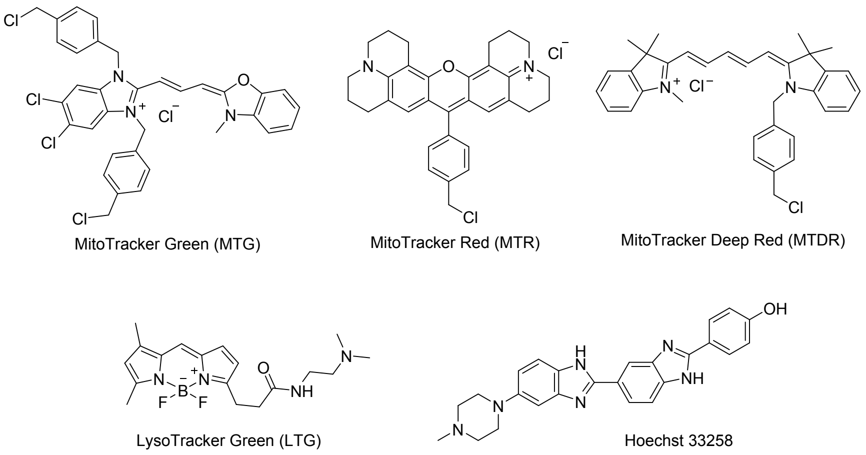

Scheme S5. Commercially available fluorescent probes used in this study.

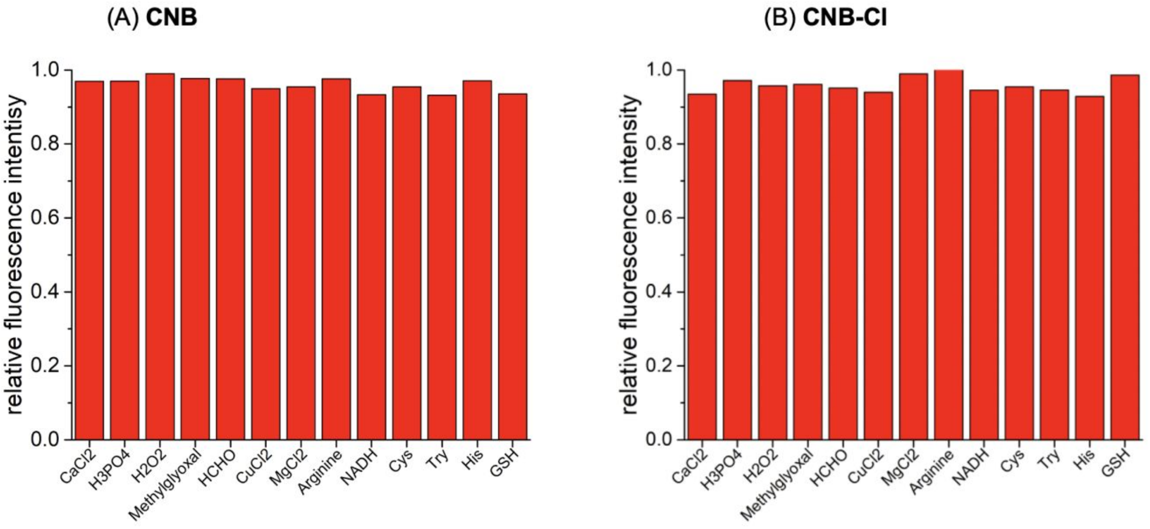

Figure S4. The stability of cationic Nile blue probes was assessed under various bio-analyte conditions. The concentrations of these analytes, with the exception of Cysteine (Cys), Tyrosine (Typ), Histidine (His), and Glutathione (GSH), were maintained at levels 100 times higher than those of the cationic Nile blue probes. In the case of Cys, Typ, His, and GSH, these were used as saturated solutions in PBS.

Table S1. Fluorescence quantum yield (Q.Y.) of cationic Nile blue probes.

Substance	Nile blue A in EtOH	**CNB** in EtOH	**CNB-Cl** in EtOH	**CNB** in PBS	**CNB-Cl** in PBS
Relative Q.Y.	1	0.61	0.56	0.13	0.12

(A)

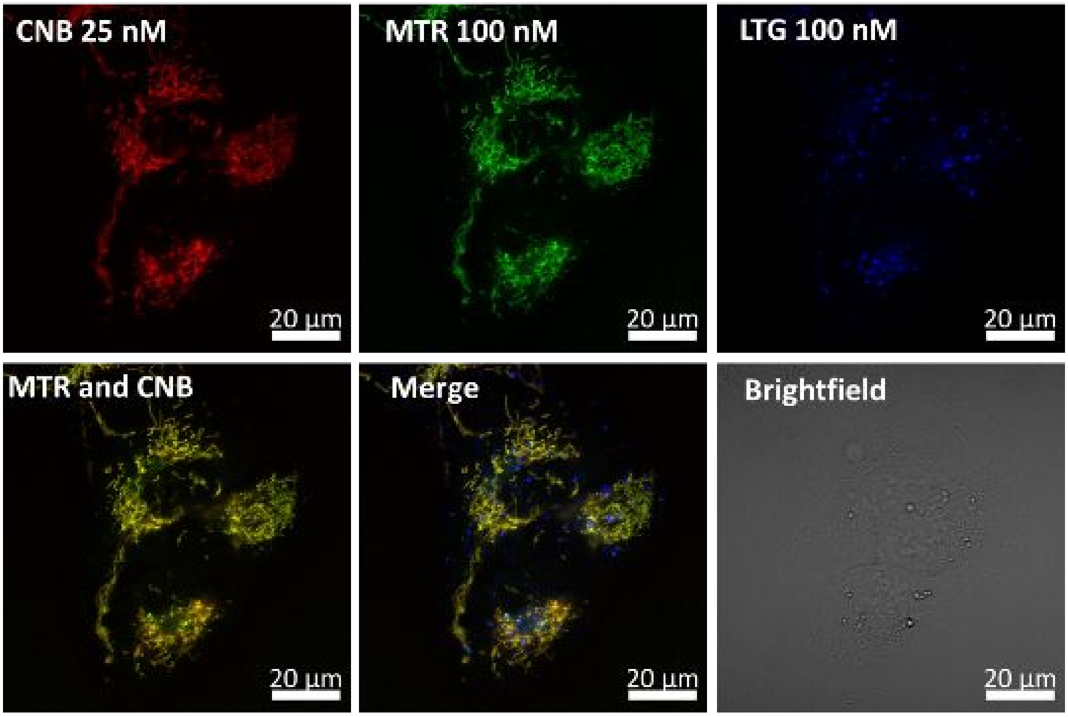

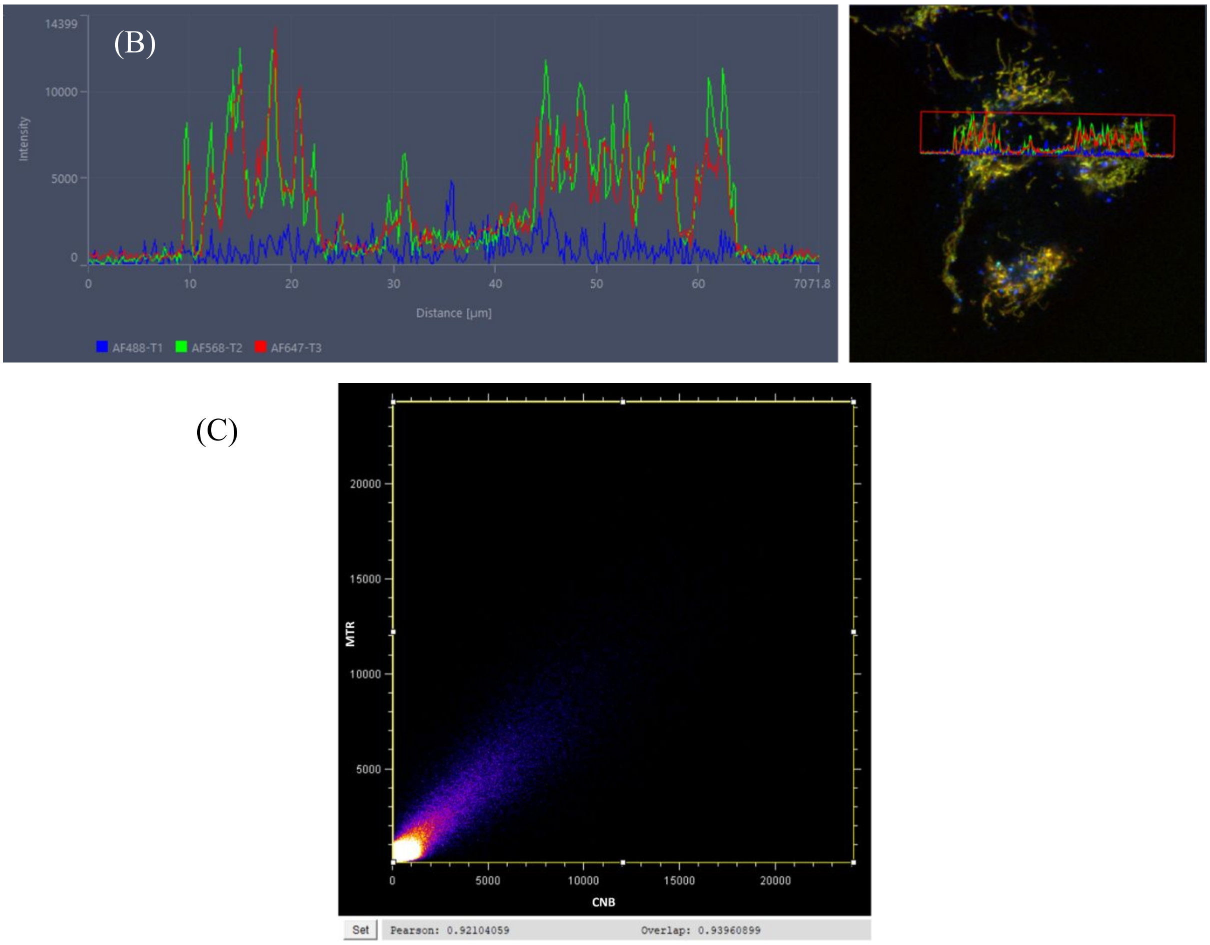

Figure S5. (A) Application of **CNB** in live cell imaging of HeLa cells. Yellow signals in the merged images arising from the co-localization fluorescence of MTR and **CNB** confirm mitochondrial accumulation of **CNB**. (B) Fluorescence line plots of the merged image in (A). (C) Correlation plot of **CNB** and MTR.

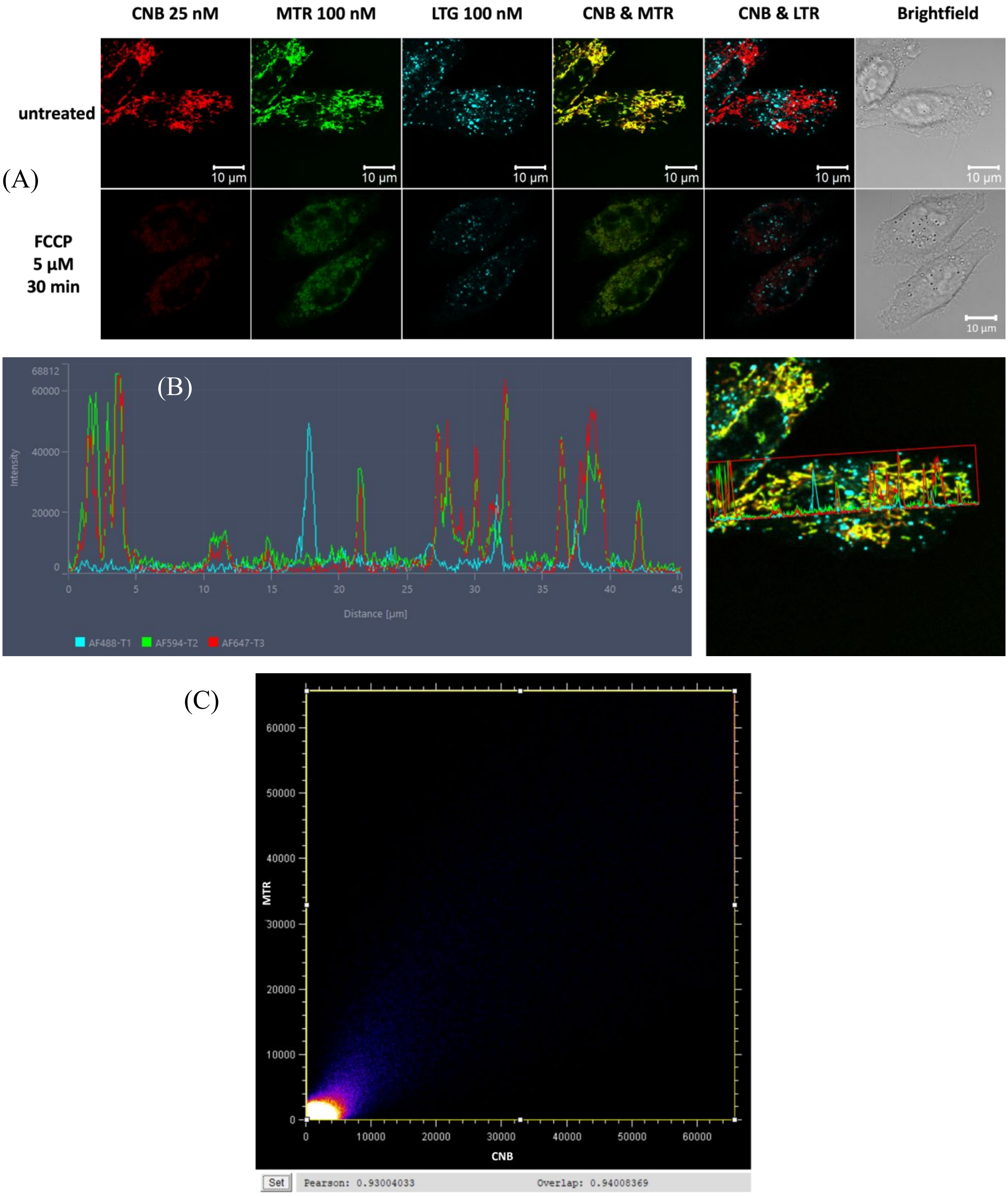

Figure S6. (A) Depolarization of mitochondria causes fluorescence vanishment of **CNB**. U-2 OS cells were incubated with solution of LTG, MTR and **CNB** at specified concentrations for 1 h. Then cells were washed with HBSS twice and treated with FCCP (5 µM) for 30 min before imaging. (B) Fluorescence line plots of the merged image in the untreated group in (A). (C) Correlation plot of **CNB** and MTR.

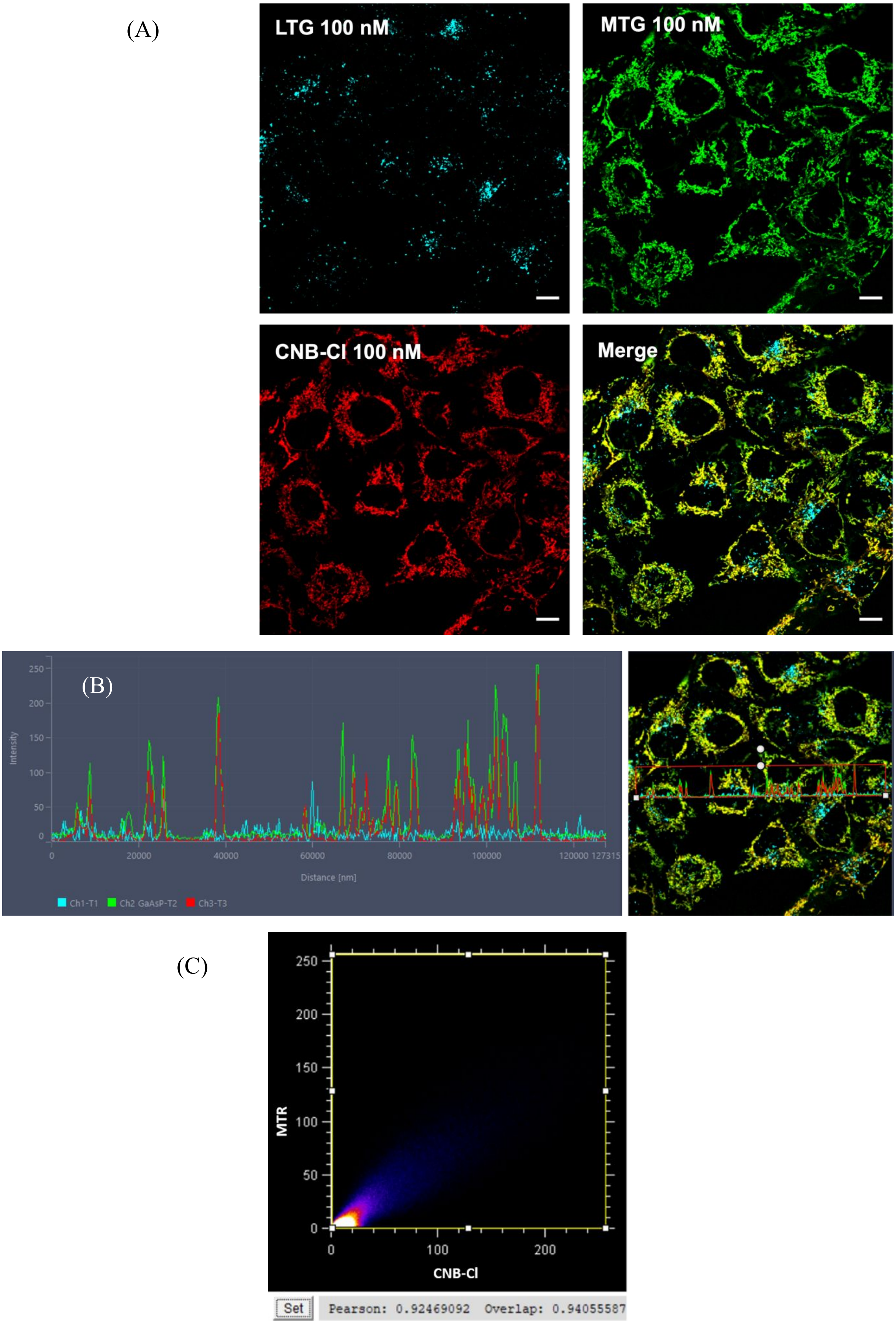
(A)
LTG 100 nM
MTG 100 nM
CNB-Cl 100 nM
Merge
(B)
Intensity
Distance [nm]
Ch1-T1
Ch2 GaAsP-T2
Ch3-T3
(C)
MTR
CNB-Cl
Set
Pearson: 0.92469092 Overlap: 0.94055587

Figure S7. (A) Application of **CNB-Cl** in live cell imaging of HeLa cells. Yellow signals in arising from the co-localization MTG and **CNB-Cl** confirm mitochondrial accumulation of **CNB-Cl**. Scale bars: 10 µm. (B) Fluorescence line plots of merged image in (A). (C) Correlation plot of **CNB** and MTR.

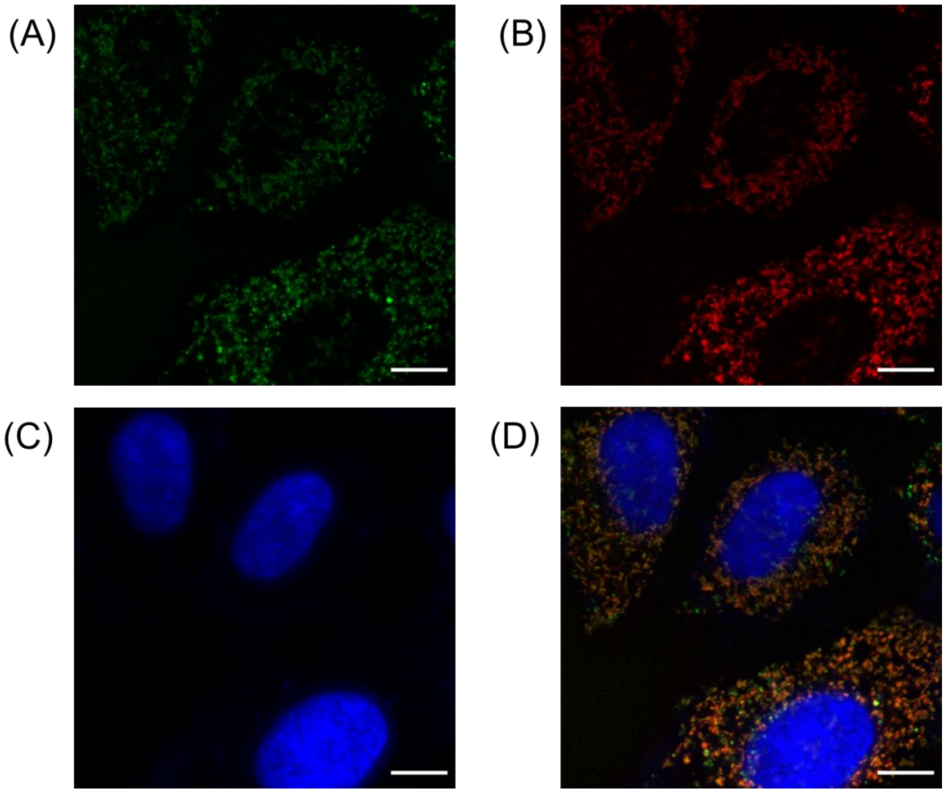

Figure S8. Fluorescence retention of **CNB-Cl** after depolarization of mitochondria by FCCP. HeLa cells were incubated with mixture of mitotracker green (MTG, 100 nM) Hoechst 33358 (2 µg/mL) and **CNB-Cl** (50 nM) in HBSS for 1 hour. Then cells were washed with HBSS twice and treated with FCCP (5 µM) for 30 min before viewed under confocal laser scanning microscopy. (A) MTG. (B) **CNB-Cl**. (C) Hoechst 33358. (D) Merged images of (A) (B) and (C). Scale bars: 10 µm.

(A)

(B)

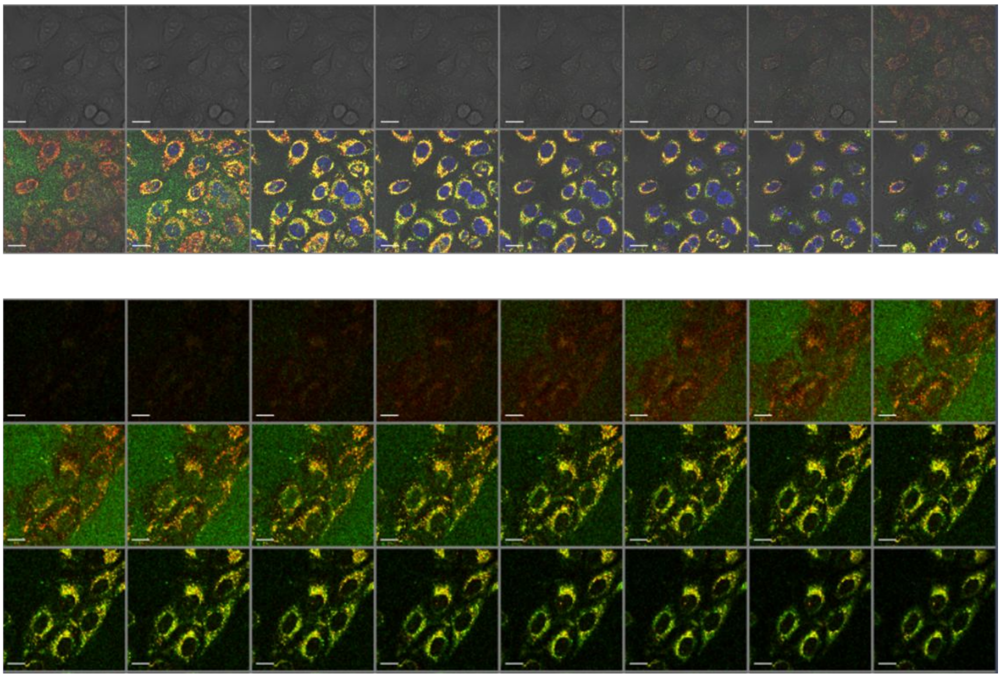

Figure S9. Comparison of background signal of **CNB-Cl** and MTG in U-2 OS cells. (A) U-2 OS cells were incubated with mitotracker green (MTG, 100 nM) and **CNB-Cl** (100 nM) for 1 hour. A set of images taken from cells so that the distance from the objective (z-axis) is different for each image but the image area along the x- and y-axes remains the same. (B) Concentrations are 500 nM for both dyes. Green colour is fluorescence of MTG. Red color is fluorescence of **CNB-Cl**. Yellow signals arise from the co-localization fluorescence of MTG and **CNB-Cl**. Scale bars, 20 μm.

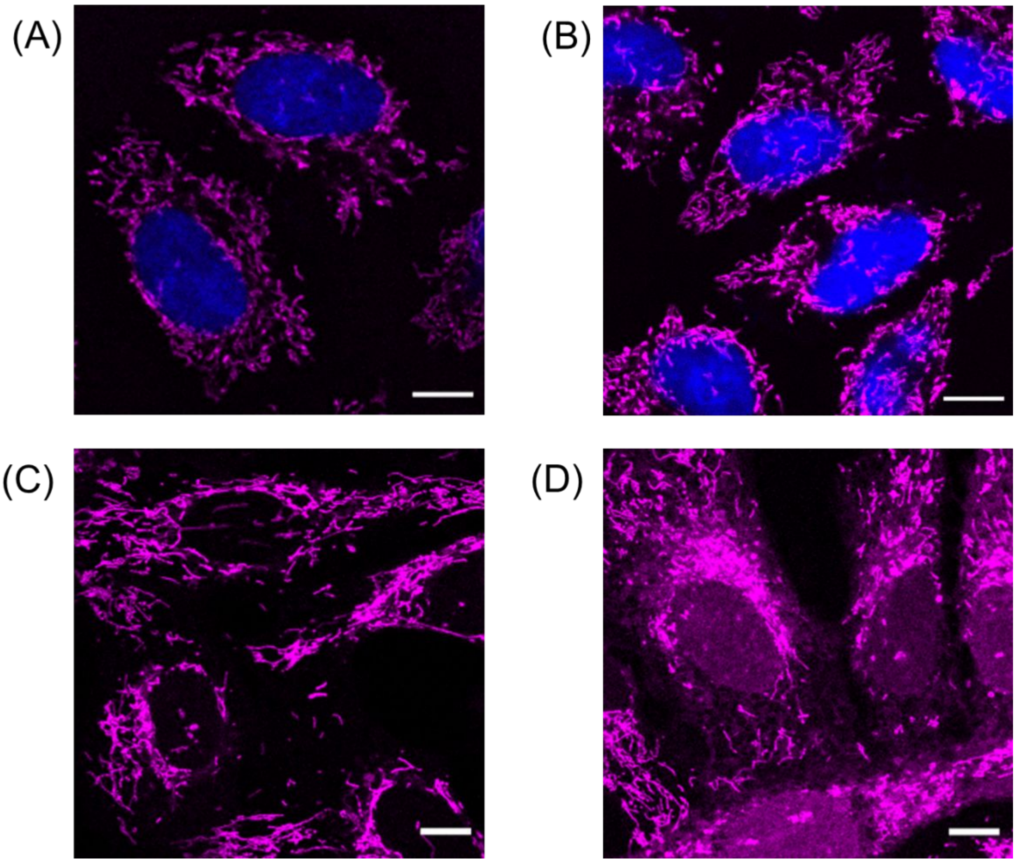

Figure S10. Evaluation of performance of **CNB-Cl** in no-wash imaging of live cells. HeLa cells were incubated with solution of **CNB-Cl** or MTDR in DMEM for 1 hour and directly viewed under confocal laser scanning microscopy. (A) 50 nM of **CNB-Cl**. (B) 250 nM of **CNB-Cl**. (C) 500 nM of **CNB-Cl**. (D) 500 nM of MTDR. Characteristic morphology of mitochondria was observed in (A) (B) and (C) with no or very weak background signals while diffusion of MTDR into cytosol and nucleus were clearly observed (D). Scale bars, 10 μm.

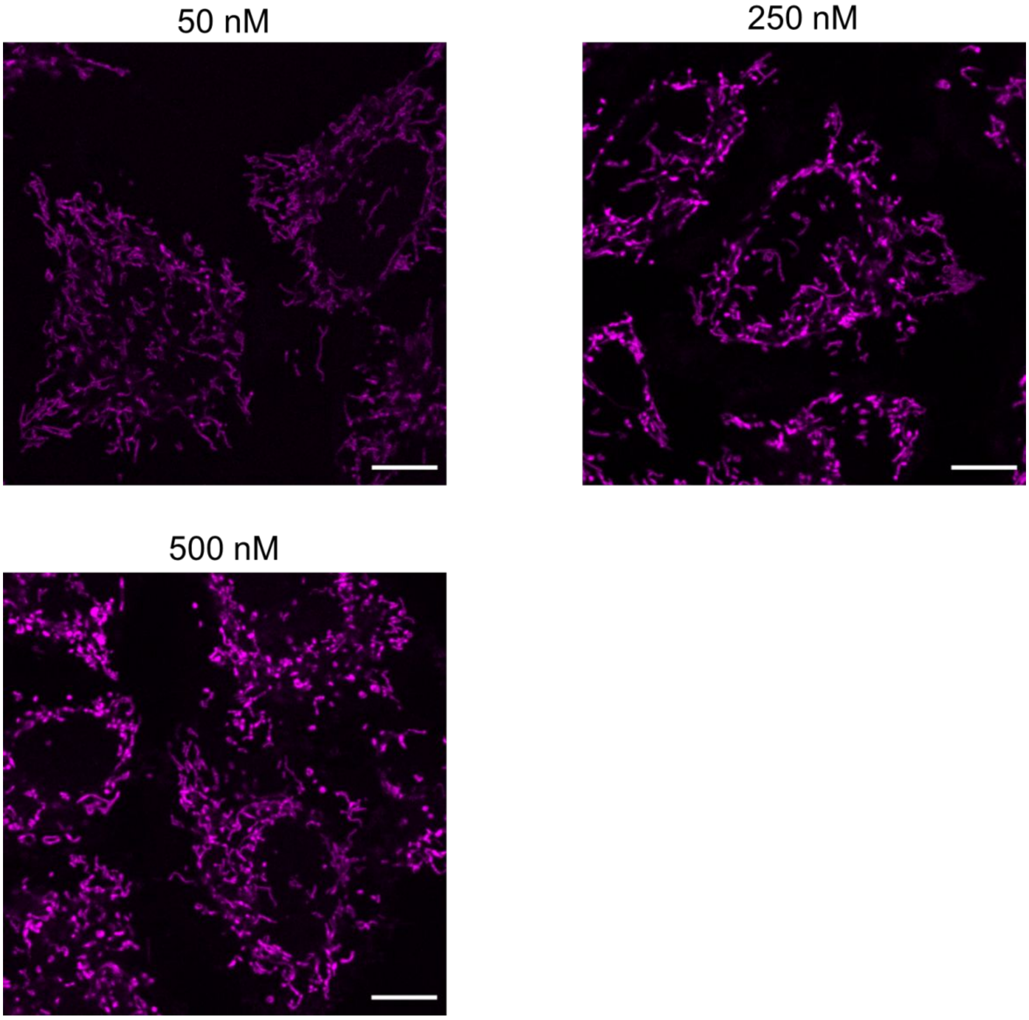

Figure S11. Evaluation of performance of **CNB** in no-wash imaging of live cells. Characteristic morphology of mitochondria was observed with no background signals at broad range of concentrations. Scale bars, 10 μm.

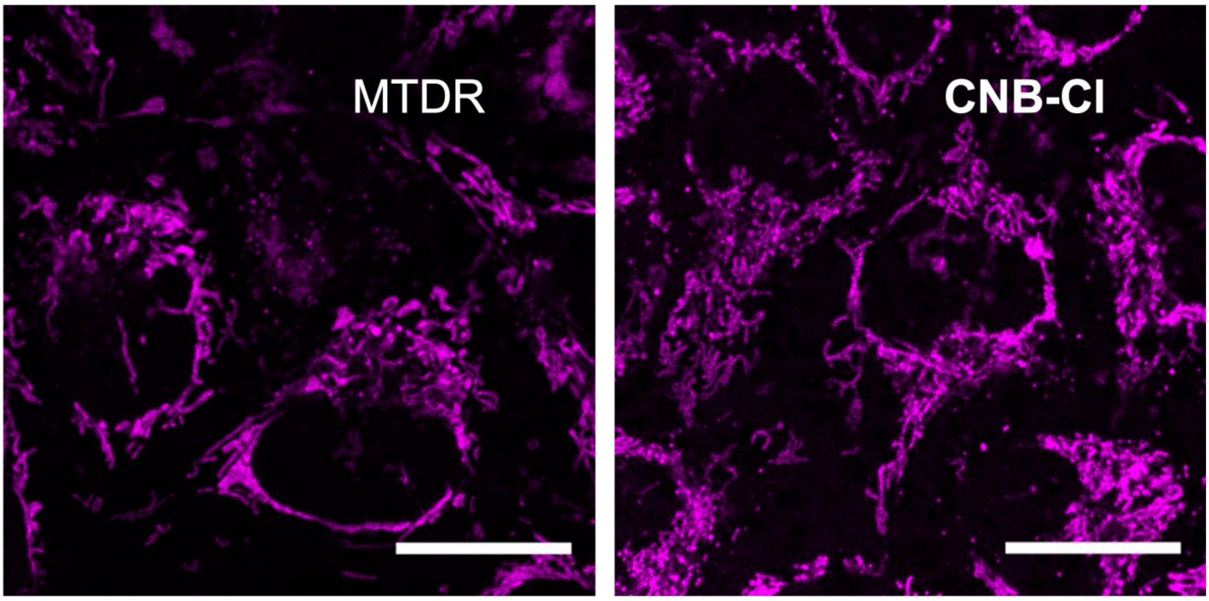

Figure S12. Mitochondrial damage effects on HeLa cells of **MTDR** and **CNB-Cl** at 100 nM after treatment for 24 hours. Scale bars, 20 μm.

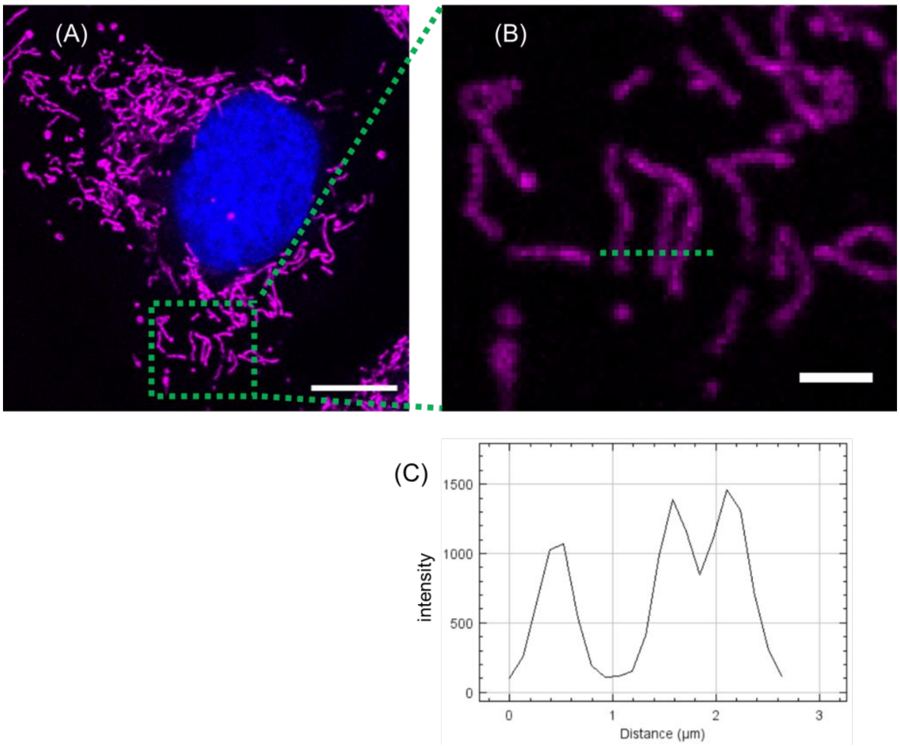

Figure S13. (A) A confocal image of typical mitochondria in U-2 OS cells stained by **CNB-Cl**. (B). Enlargement of the region boxed in figure (A). (C) Transverse profile of single mitochondrion along the green dotted line in figure (B). It indicates the FWHM of single mitochondrion in confocal image is around 400 nm. Scale bars: (A) 10 μm, (B) 2 μm.

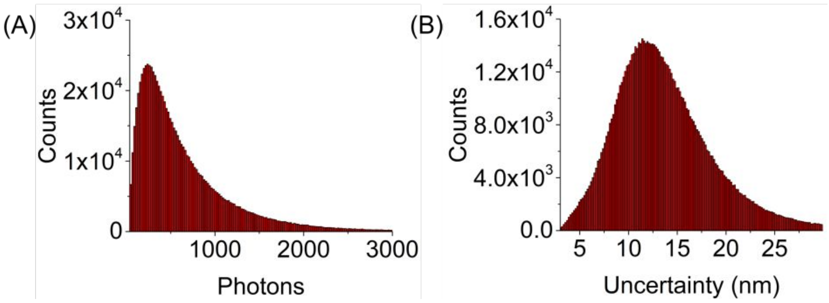

Figure S14. (A) Histogram of photon numbers per dye molecule **CNB-Cl** per imaging frame. (B) Histogram of localization uncertainty per dye molecule per imaging frame. Corresponding to Figure 2D.

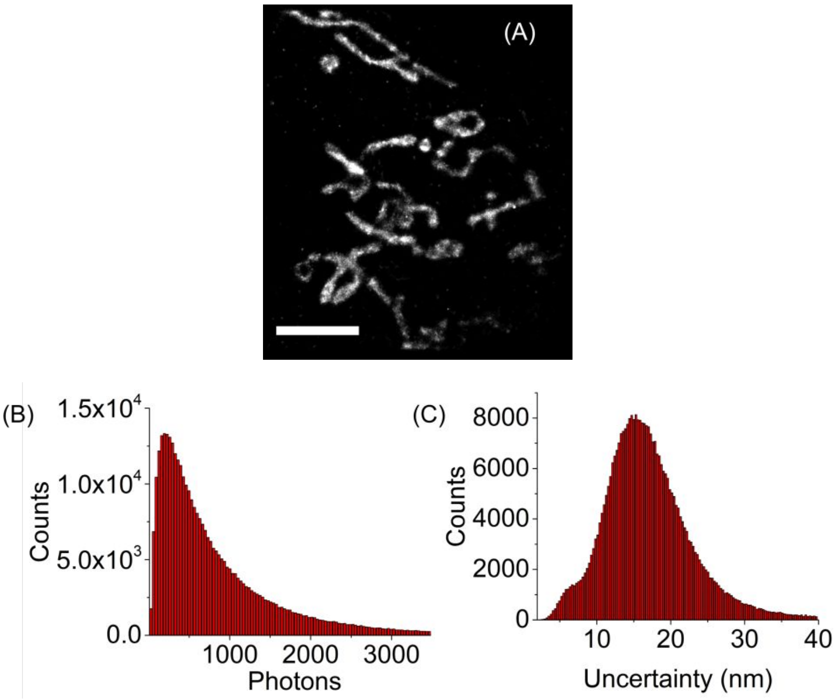

Figure S15. (A) A typical SMLM image of mitochondria in living U-2 OS cells stained by **CNB-Cl**. (B) Histogram of photon numbers per dye molecule per imaging frame. (C) Histogram of localization uncertainty per dye molecule per imaging frame. Scale bar, 4 μm.

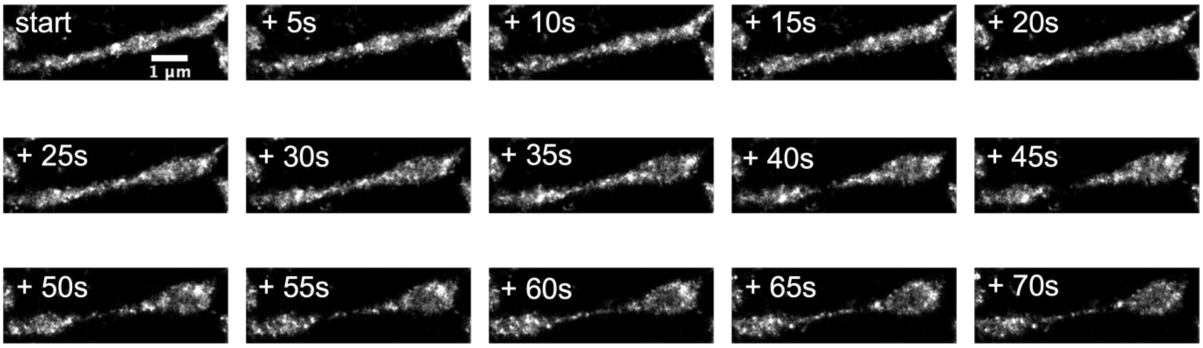

Figure S16. Mitochondria dynamics in live Hela cells were revealed by **CNB-Cl** uncer SMLM condition. Each image was reconstructed with 2000 consecutive frames under rate of 100 frame/sec.

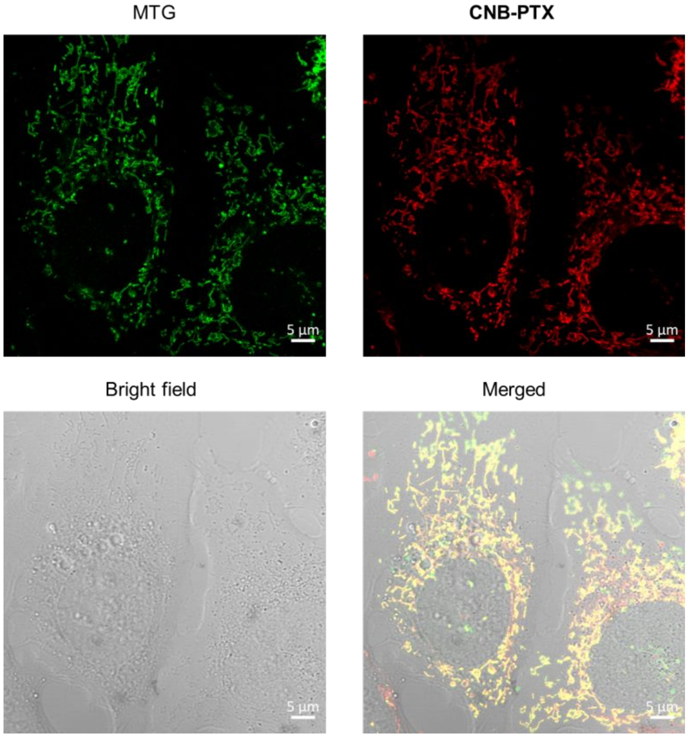

Figure S17. Subcellular localization of **CNB-PTX** in live cells. HeLa cells were incubated with solution of Mitotracker green (MTG, 100 nM) and **CNB-PTX** (2 μM). Yellow signals in the merged images arising from the co-localization fluorescence of MTG and **CNB-PTX** confirm mitochondrial accumulation of **CNB-PTX**.

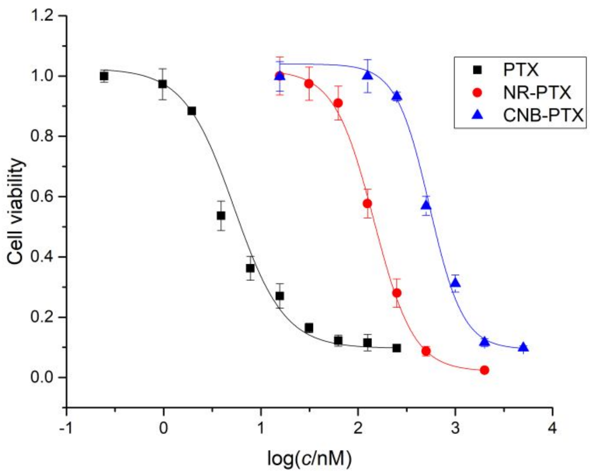

Figure S18. Evaluation viability of HeLa cells in the prescence of paclitaxel derivatives, **CNB-PTX** and **NR-**PTX in Hela cells at 48 h. (IC_{50} value: paclitaxel 8 nM, **NR-PTX** 145 nM, **CNB-PTX** 543 nM).

7. Spectra

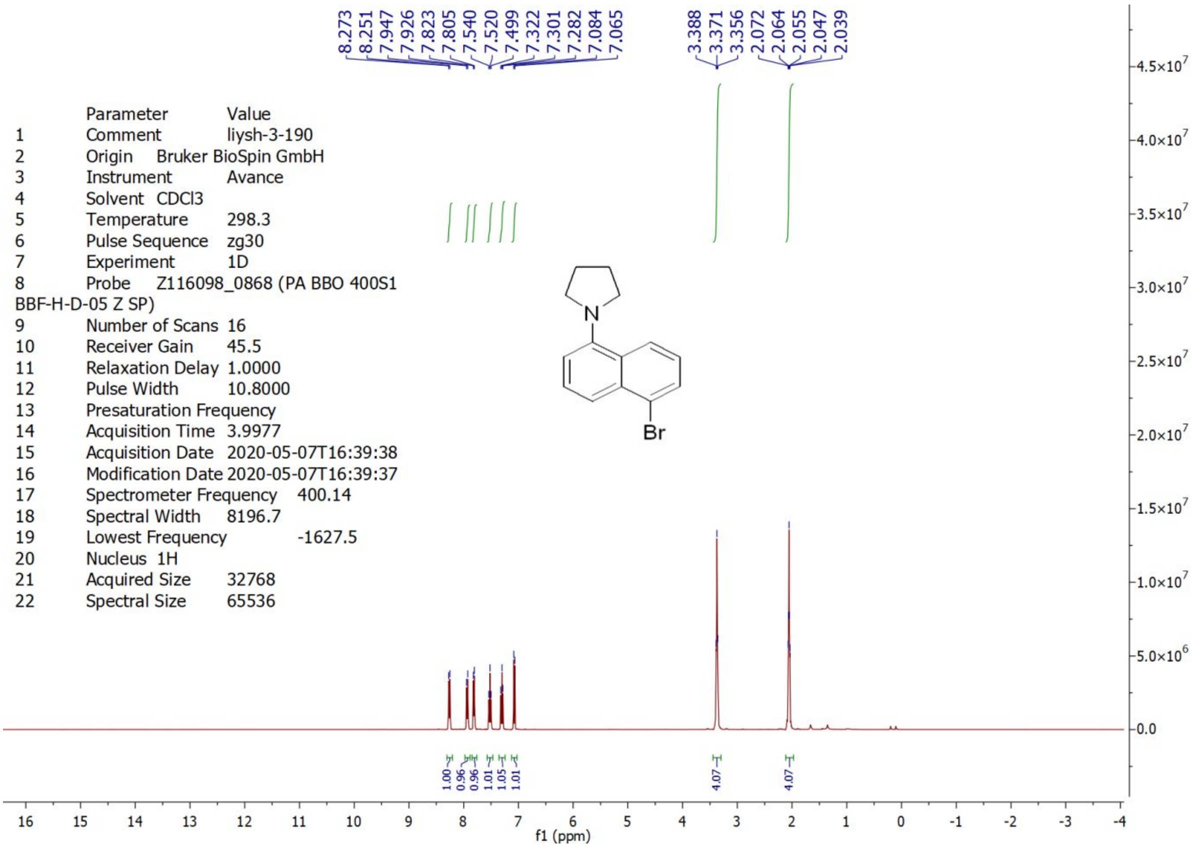
8.273
8.251
7.947
7.926
7.823
7.805
7.540
7.520
7.499
7.322
7.301
7.282
7.084
7.065
3.388
3.371
3.356
2.072
2.064
2.055
2.047
2.039
Parameter Value
1 Comment liysh-3-190
2 Origin Bruker BioSpin GmbH
3 Instrument Avance
4 Solvent CDCl3
5 Temperature 298.3
6 Pulse Sequence zg30
7 Experiment 1D
8 Probe Z116098_0868 (PA BBO 400S1 BBF-H-D-05 Z SP)
9 Number of Scans 16
10 Receiver Gain 45.5
11 Relaxation Delay 1.0000
12 Pulse Width 10.8000
13 Presaturation Frequency
14 Acquisition Time 3.9977
15 Acquisition Date 2020-05-07T16:39:38
16 Modification Date 2020-05-07T16:39:37
17 Spectrometer Frequency 400.14
18 Spectral Width 8196.7
19 Lowest Frequency -1627.5
20 Nucleus 1H
21 Acquired Size 32768
22 Spectral Size 65536
N
Br
1.00
0.96
0.96
1.01
1.05
1.01
4.07
4.07
f1 (ppm)

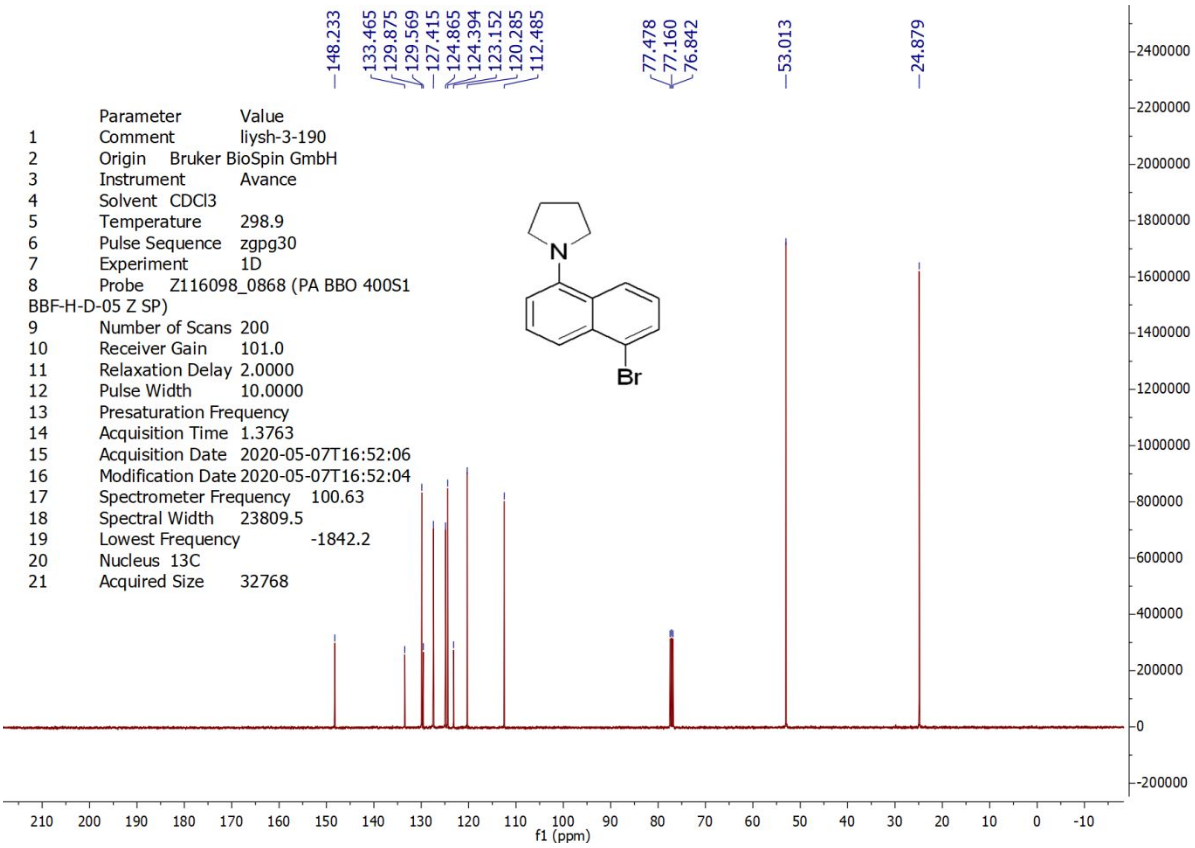
148.233
133.465
129.875
129.569
127.415
124.865
124.394
123.152
120.285
112.485
77.478
77.160
76.842
53.013
24.879
Parameter Value
1 Comment liysh-3-190
2 Origin Bruker BioSpin GmbH
3 Instrument Avance
4 Solvent CDCl3
5 Temperature 298.9
6 Pulse Sequence zgpg30
7 Experiment 1D
8 Probe Z116098_0868 (PA BBO 400S1 BBF-H-D-05 Z SP)
9 Number of Scans 200
10 Receiver Gain 101.0
11 Relaxation Delay 2.0000
12 Pulse Width 10.0000
13 Presaturation Frequency
14 Acquisition Time 1.3763
15 Acquisition Date 2020-05-07T16:52:06
16 Modification Date 2020-05-07T16:52:04
17 Spectrometer Frequency 100.63
18 Spectral Width 23809.5
19 Lowest Frequency -1842.2
20 Nucleus 13C
21 Acquired Size 32768
N
Br
f1 (ppm)

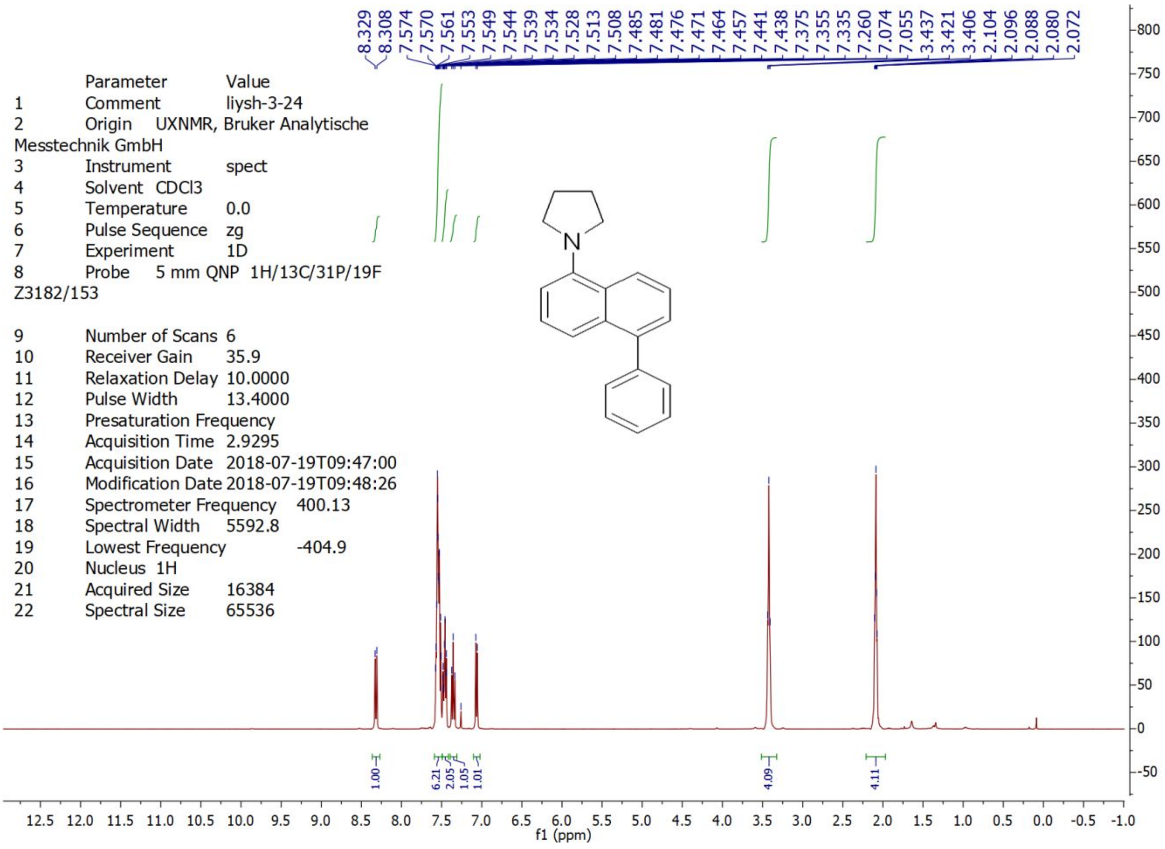
8.329
8.308
7.574
7.570
7.561
7.553
7.549
7.544
7.539
7.534
7.528
7.513
7.508
7.485
7.481
7.476
7.471
7.464
7.457
7.441
7.438
7.375
7.355
7.335
7.260
7.074
7.055
3.437
3.421
3.406
2.104
2.096
2.088
2.080
2.072
Parameter Value
1 Comment liysh-3-24
2 Origin UXNMR, Bruker Analytische Messtechnik GmbH
3 Instrument spect
4 Solvent CDCl3
5 Temperature 0.0
6 Pulse Sequence zg
7 Experiment 1D
8 Probe 5 mm QNP 1H/13C/31P/19F Z3182/153
9 Number of Scans 6
10 Receiver Gain 35.9
11 Relaxation Delay 10.0000
12 Pulse Width 13.4000
13 Presaturation Frequency
14 Acquisition Time 2.9295
15 Acquisition Date 2018-07-19T09:47:00
16 Modification Date 2018-07-19T09:48:26
17 Spectrometer Frequency 400.13
18 Spectral Width 5592.8
19 Lowest Frequency -404.9
20 Nucleus 1H
21 Acquired Size 16384
22 Spectral Size 65536
N
1.00
6.21
2.05
1.05
1.01
4.09
4.11
f1 (ppm)

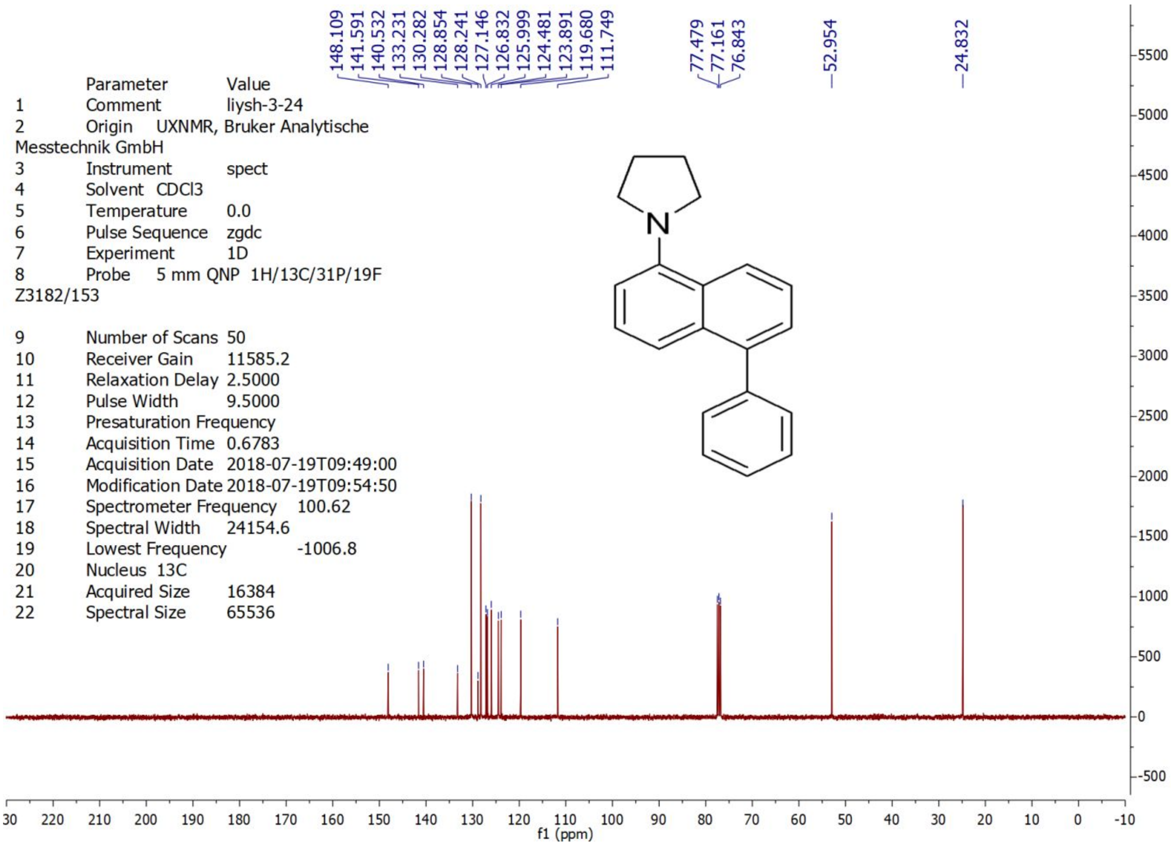
148.109
141.591
140.532
133.231
130.282
128.854
128.241
127.146
126.832
125.999
124.481
123.891
119.680
111.749
77.479
77.161
76.843
52.954
24.832
Parameter Value
1 Comment liysh-3-24
2 Origin UXNMR, Bruker Analytische Messtechnik GmbH
3 Instrument spect
4 Solvent CDCl3
5 Temperature 0.0
6 Pulse Sequence zgdc
7 Experiment 1D
8 Probe 5 mm QNP 1H/13C/31P/19F Z3182/153
9 Number of Scans 50
10 Receiver Gain 11585.2
11 Relaxation Delay 2.5000
12 Pulse Width 9.5000
13 Presaturation Frequency
14 Acquisition Time 0.6783
15 Acquisition Date 2018-07-19T09:49:00
16 Modification Date 2018-07-19T09:54:50
17 Spectrometer Frequency 100.62
18 Spectral Width 24154.6
19 Lowest Frequency -1006.8
20 Nucleus 13C
21 Acquired Size 16384
22 Spectral Size 65536
N
f1 (ppm)

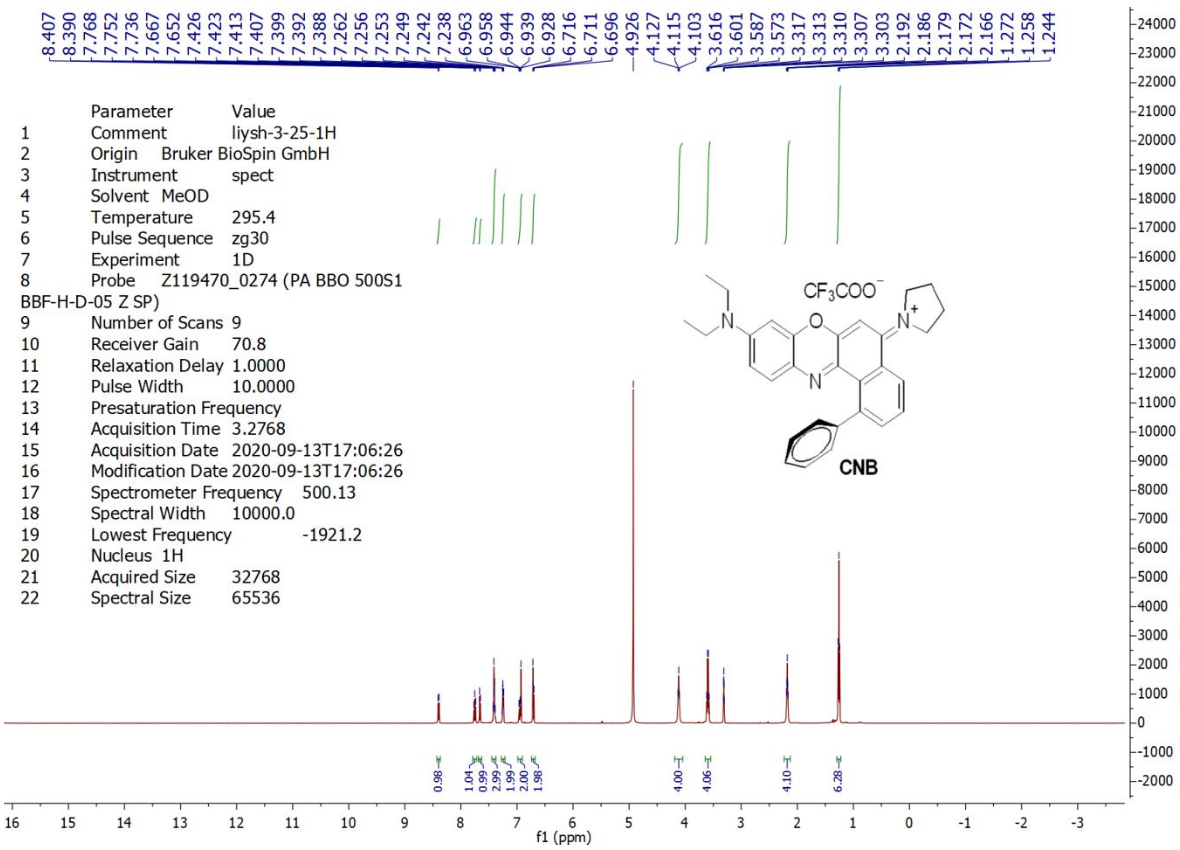
8.407 8.390 7.768 7.752 7.736 7.667 7.652 7.426 7.423 7.413 7.407 7.399 7.392 7.388 7.262 7.256 7.253 7.249 7.242 7.238 6.963 6.958 6.944 6.939 6.928 6.716 6.711 6.696 4.926 4.127 4.115 4.103 3.616 3.601 3.587 3.573 3.317 3.313 3.310 3.307 3.303 2.192 2.186 2.179 2.172 2.166 1.272 1.258 1.244
Parameter Value
1 Comment liysh-3-25-1H
2 Origin Bruker BioSpin GmbH
3 Instrument spect
4 Solvent MeOD
5 Temperature 295.4
6 Pulse Sequence zg30
7 Experiment 1D
8 Probe Z119470_0274 (PA BBO 500S1 BBF-H-D-05 Z SP)
9 Number of Scans 9
10 Receiver Gain 70.8
11 Relaxation Delay 1.0000
12 Pulse Width 10.0000
13 Presaturation Frequency
14 Acquisition Time 3.2768
15 Acquisition Date 2020-09-13T17:06:26
16 Modification Date 2020-09-13T17:06:26
17 Spectrometer Frequency 500.13
18 Spectral Width 10000.0
19 Lowest Frequency -1921.2
20 Nucleus 1H
21 Acquired Size 32768
22 Spectral Size 65536
CF3COO−
CNB
0.98 1.04 0.99 2.99 1.99 2.00 1.98 4.00 4.06 4.10 6.28
f1 (ppm)

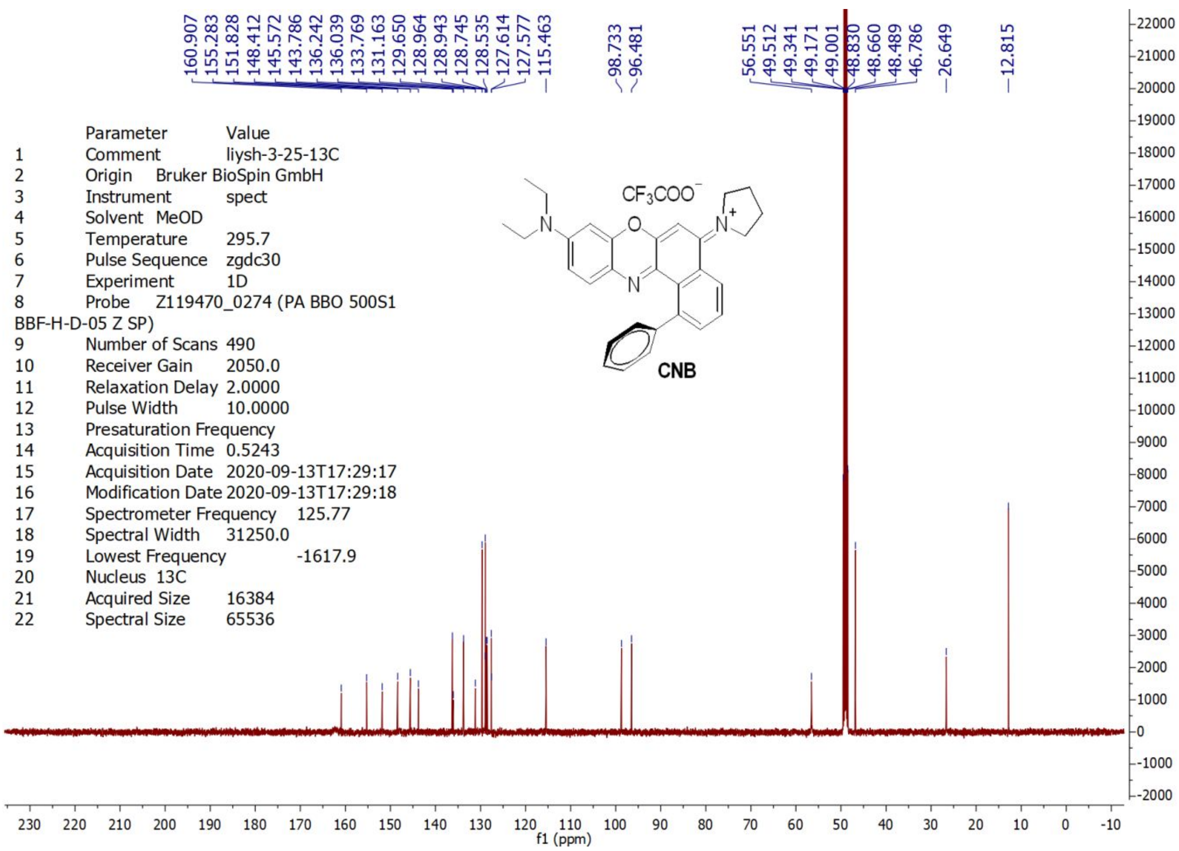
160.907 155.283 151.828 148.412 145.572 143.786 136.242 136.039 133.769 131.163 129.650 128.964 128.943 128.745 128.535 127.614 127.577 115.463 98.733 96.481 56.551 49.512 49.341 49.171 49.001 48.830 48.660 48.489 46.786 26.649 12.815
Parameter Value
1 Comment liysh-3-25-13C
2 Origin Bruker BioSpin GmbH
3 Instrument spect
4 Solvent MeOD
5 Temperature 295.7
6 Pulse Sequence zgdc30
7 Experiment 1D
8 Probe Z119470_0274 (PA BBO 500S1 BBF-H-D-05 Z SP)
9 Number of Scans 490
10 Receiver Gain 2050.0
11 Relaxation Delay 2.0000
12 Pulse Width 10.0000
13 Presaturation Frequency
14 Acquisition Time 0.5243
15 Acquisition Date 2020-09-13T17:29:17
16 Modification Date 2020-09-13T17:29:18
17 Spectrometer Frequency 125.77
18 Spectral Width 31250.0
19 Lowest Frequency -1617.9
20 Nucleus 13C
21 Acquired Size 16384
22 Spectral Size 65536
CF3COO−
CNB
f1 (ppm)

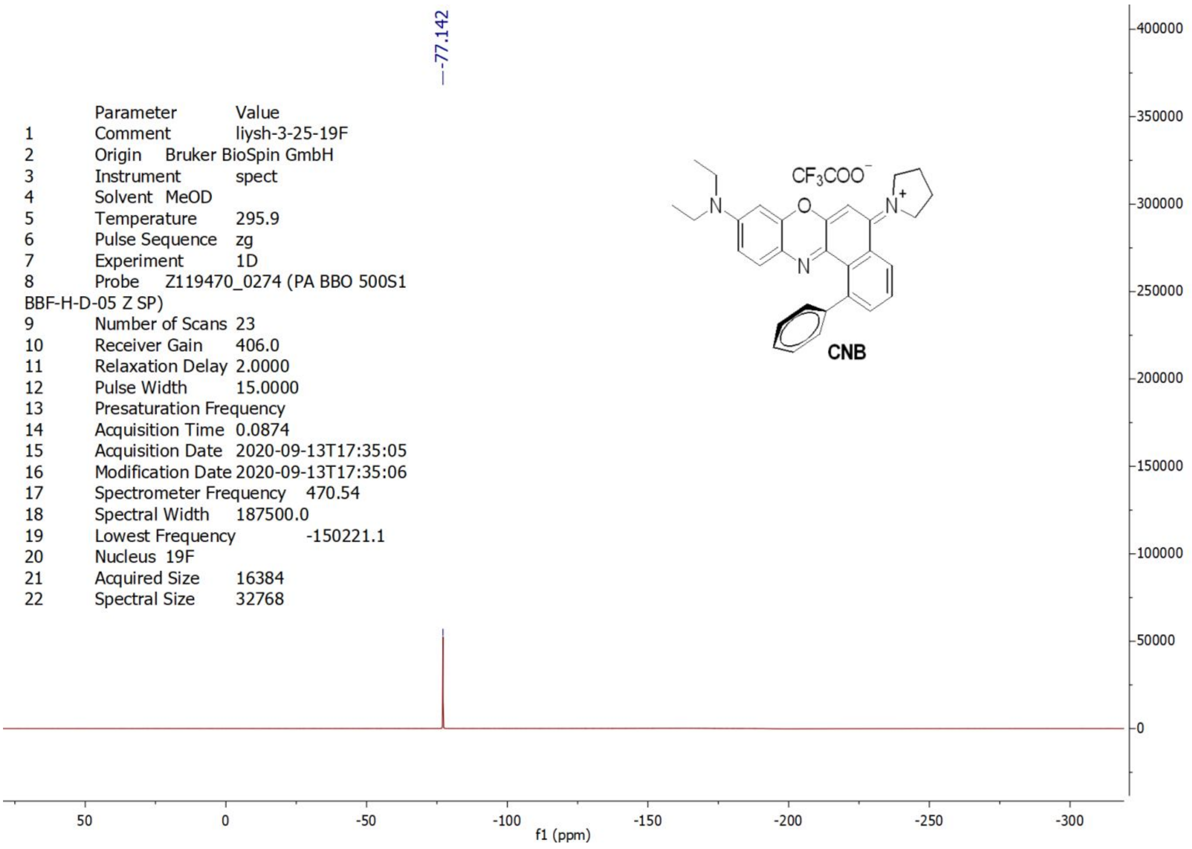
--77.142
Parameter Value
1 Comment liysh-3-25-19F
2 Origin Bruker BioSpin GmbH
3 Instrument spect
4 Solvent MeOD
5 Temperature 295.9
6 Pulse Sequence zg
7 Experiment 1D
8 Probe Z119470_0274 (PA BBO 500S1 BBF-H-D-05 Z SP)
9 Number of Scans 23
10 Receiver Gain 406.0
11 Relaxation Delay 2.0000
12 Pulse Width 15.0000
13 Presaturation Frequency
14 Acquisition Time 0.0874
15 Acquisition Date 2020-09-13T17:35:05
16 Modification Date 2020-09-13T17:35:06
17 Spectrometer Frequency 470.54
18 Spectral Width 187500.0
19 Lowest Frequency -150221.1
20 Nucleus 19F
21 Acquired Size 16384
22 Spectral Size 32768
CF3COO⁻
CNB
f1 (ppm)

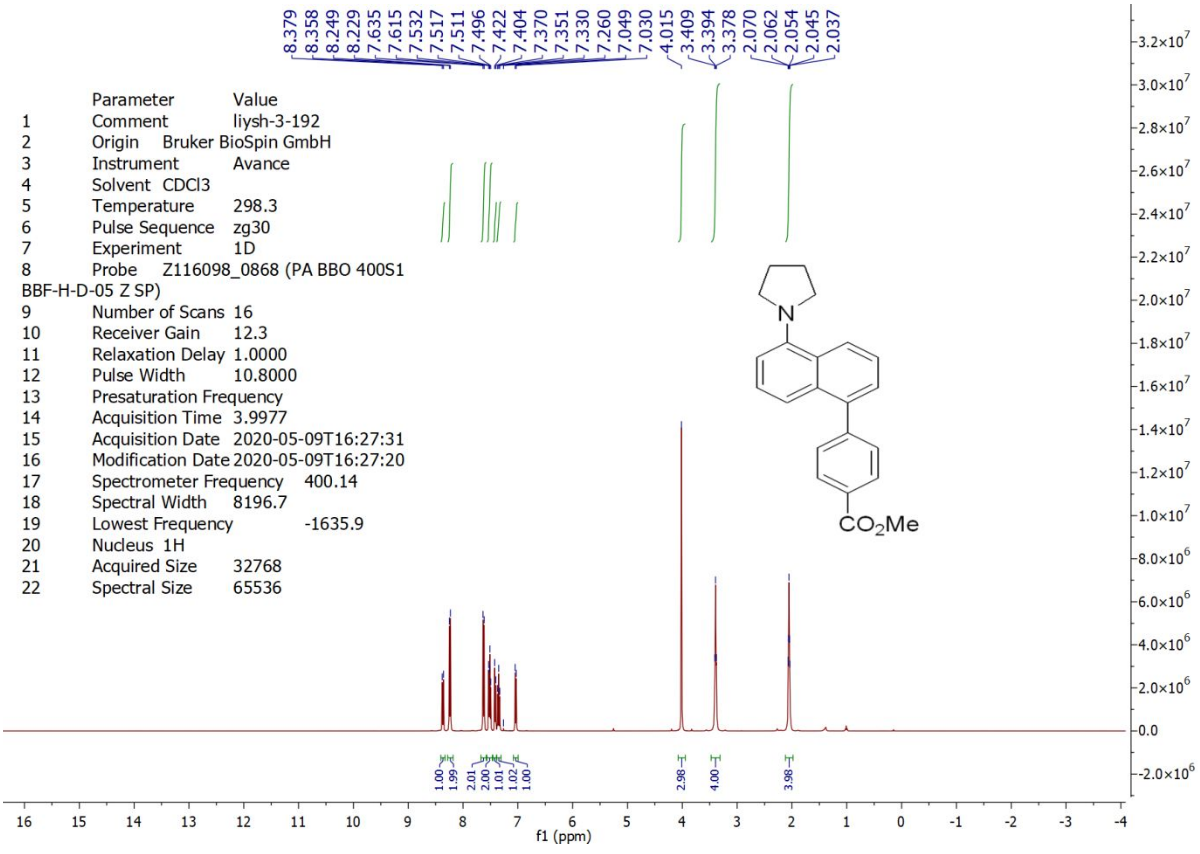
8.379 8.358 8.249 8.229 7.635 7.615 7.532 7.517 7.511 7.496 7.422 7.404 7.370 7.351 7.330 7.260 7.049 7.030 4.015 3.409 3.394 3.378 2.070 2.062 2.054 2.045 2.037
Parameter Value
1 Comment liysh-3-192
2 Origin Bruker BioSpin GmbH
3 Instrument Avance
4 Solvent CDCl3
5 Temperature 298.3
6 Pulse Sequence zg30
7 Experiment 1D
8 Probe Z116098_0868 (PA BBO 400S1 BBF-H-D-05 Z SP)
9 Number of Scans 16
10 Receiver Gain 12.3
11 Relaxation Delay 1.0000
12 Pulse Width 10.8000
13 Presaturation Frequency
14 Acquisition Time 3.9977
15 Acquisition Date 2020-05-09T16:27:31
16 Modification Date 2020-05-09T16:27:20
17 Spectrometer Frequency 400.14
18 Spectral Width 8196.7
19 Lowest Frequency -1635.9
20 Nucleus 1H
21 Acquired Size 32768
22 Spectral Size 65536
CO2Me
1.00 1.99 2.01 2.00 1.01 1.02 1.00 2.98 4.00 3.98
f1 (ppm)

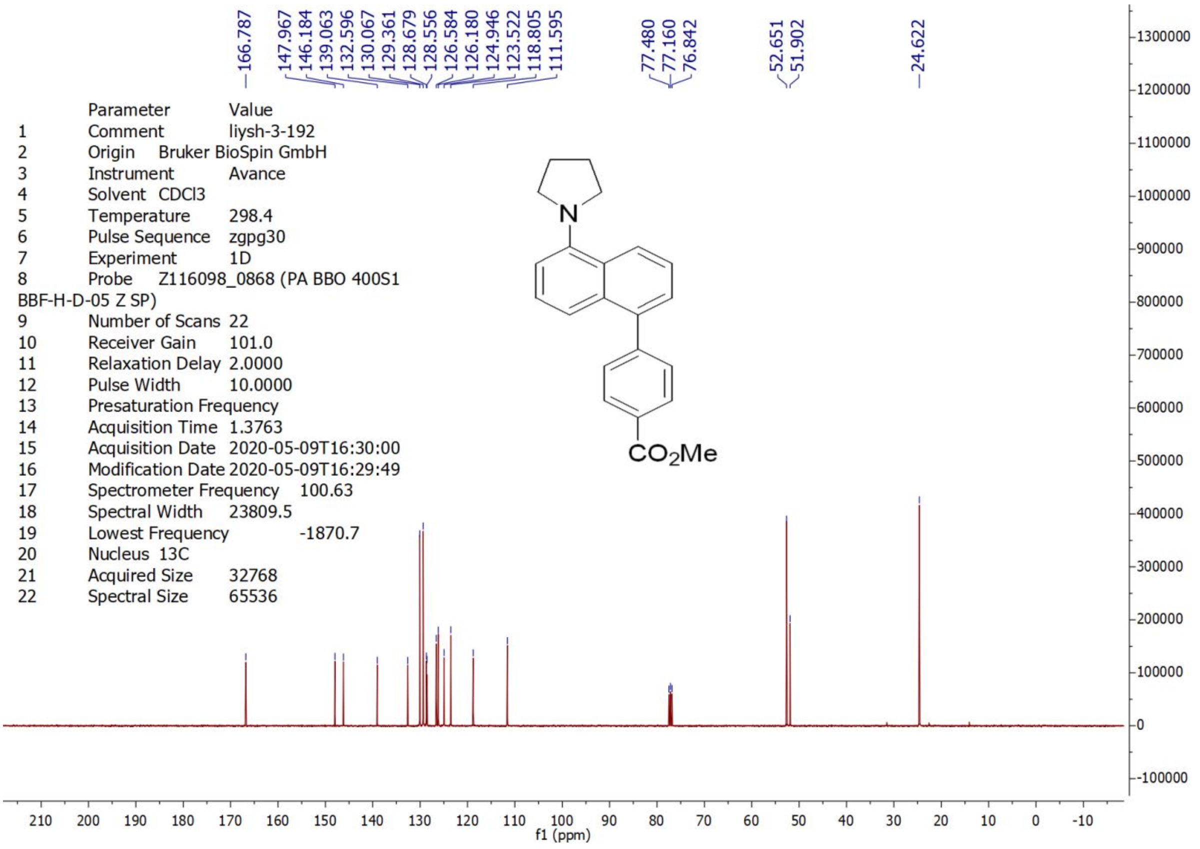
166.787
147.967
146.184
139.063
132.596
130.067
129.361
128.679
128.556
126.584
126.180
124.946
123.522
118.805
111.595
77.480
77.160
76.842
52.651
51.902
24.622
Parameter Value
1 Comment liysh-3-192
2 Origin Bruker BioSpin GmbH
3 Instrument Avance
4 Solvent CDCl3
5 Temperature 298.4
6 Pulse Sequence zgpg30
7 Experiment 1D
8 Probe Z116098_0868 (PA BBO 400S1 BBF-H-D-05 Z SP)
9 Number of Scans 22
10 Receiver Gain 101.0
11 Relaxation Delay 2.0000
12 Pulse Width 10.0000
13 Presaturation Frequency
14 Acquisition Time 1.3763
15 Acquisition Date 2020-05-09T16:30:00
16 Modification Date 2020-05-09T16:29:49
17 Spectrometer Frequency 100.63
18 Spectral Width 23809.5
19 Lowest Frequency -1870.7
20 Nucleus 13C
21 Acquired Size 32768
22 Spectral Size 65536
N
CO2Me
f1 (ppm)

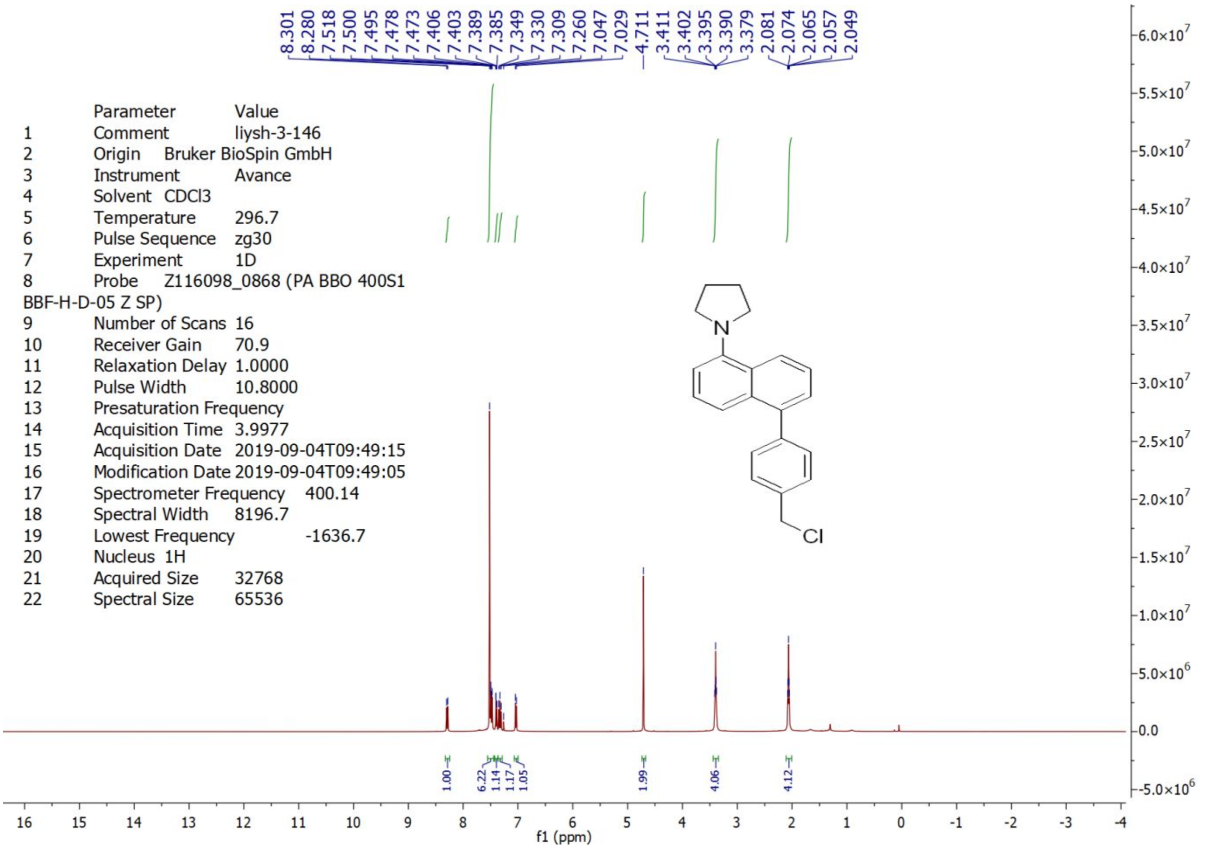
8.301
8.280
7.518
7.500
7.495
7.478
7.473
7.406
7.403
7.389
7.385
7.349
7.330
7.309
7.260
7.047
7.029
4.711
3.411
3.402
3.395
3.390
3.379
2.081
2.074
2.065
2.057
2.049
Parameter Value
1 Comment liysh-3-146
2 Origin Bruker BioSpin GmbH
3 Instrument Avance
4 Solvent CDCl3
5 Temperature 296.7
6 Pulse Sequence zg30
7 Experiment 1D
8 Probe Z116098_0868 (PA BBO 400S1 BBF-H-D-05 Z SP)
9 Number of Scans 16
10 Receiver Gain 70.9
11 Relaxation Delay 1.0000
12 Pulse Width 10.8000
13 Presaturation Frequency
14 Acquisition Time 3.9977
15 Acquisition Date 2019-09-04T09:49:15
16 Modification Date 2019-09-04T09:49:05
17 Spectrometer Frequency 400.14
18 Spectral Width 8196.7
19 Lowest Frequency -1636.7
20 Nucleus 1H
21 Acquired Size 32768
22 Spectral Size 65536
N
Cl
1.00
6.22
1.14
1.17
1.05
1.99
4.06
4.12
f1 (ppm)

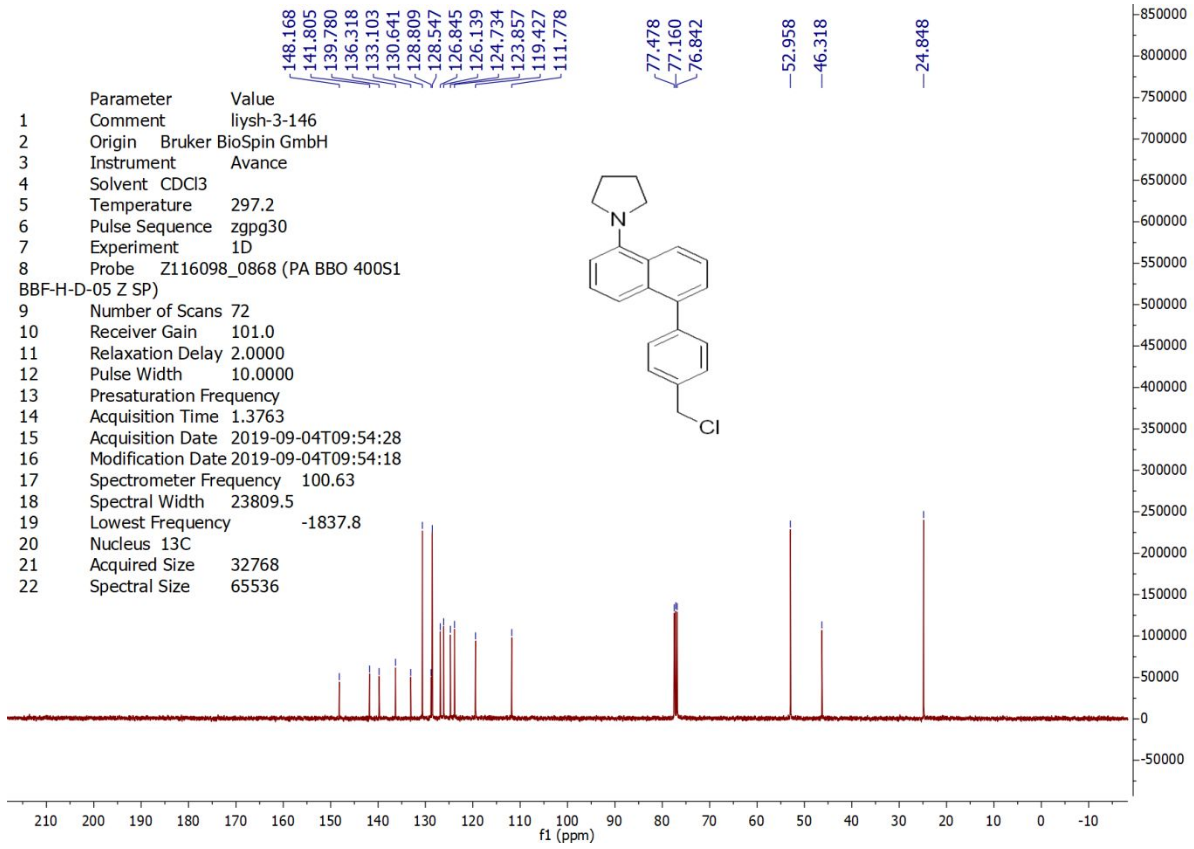
148.168
141.805
139.780
136.318
133.103
130.641
128.809
128.547
126.845
126.139
124.734
123.857
119.427
111.778
77.478
77.160
76.842
52.958
46.318
24.848
Parameter Value
1 Comment liysh-3-146
2 Origin Bruker BioSpin GmbH
3 Instrument Avance
4 Solvent CDCl3
5 Temperature 297.2
6 Pulse Sequence zgpg30
7 Experiment 1D
8 Probe Z116098_0868 (PA BBO 400S1 BBF-H-D-05 Z SP)
9 Number of Scans 72
10 Receiver Gain 101.0
11 Relaxation Delay 2.0000
12 Pulse Width 10.0000
13 Presaturation Frequency
14 Acquisition Time 1.3763
15 Acquisition Date 2019-09-04T09:54:28
16 Modification Date 2019-09-04T09:54:18
17 Spectrometer Frequency 100.63
18 Spectral Width 23809.5
19 Lowest Frequency -1837.8
20 Nucleus 13C
21 Acquired Size 32768
22 Spectral Size 65536
N
Cl
f1 (ppm)

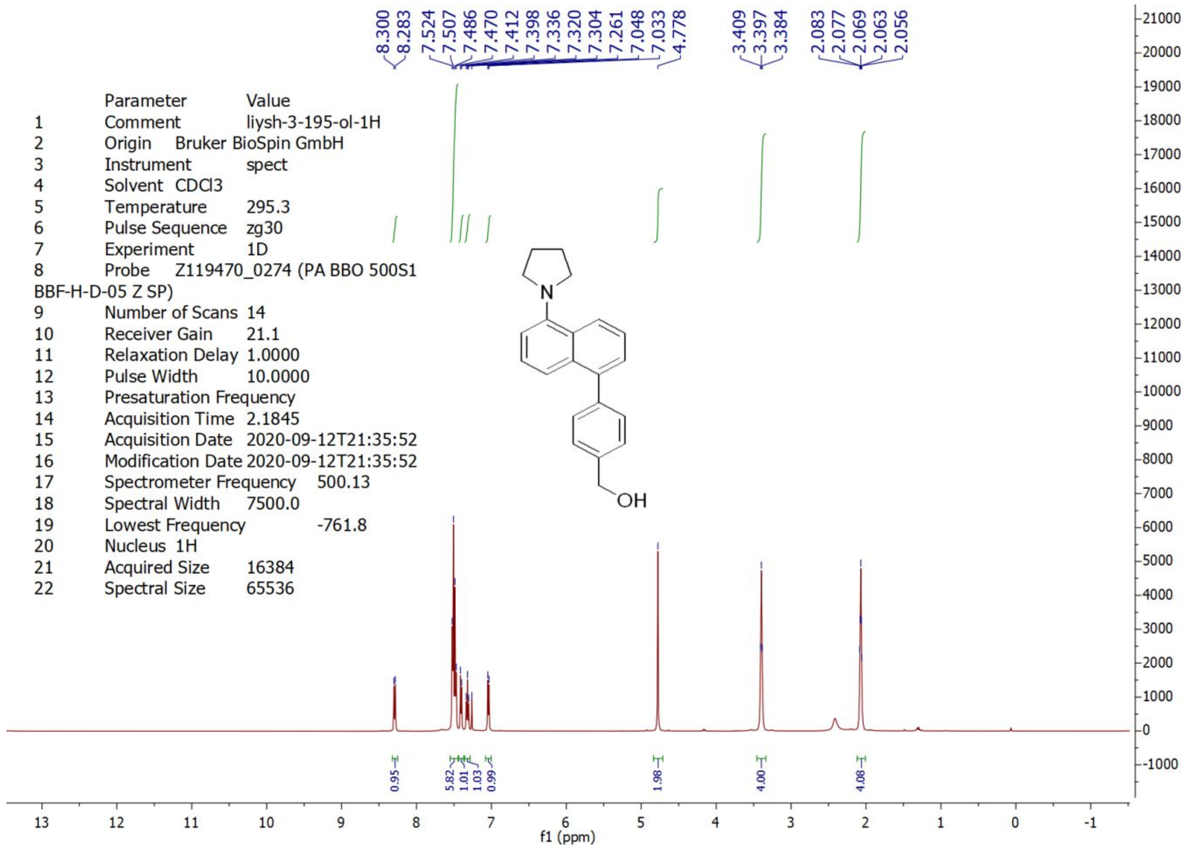
8.300
8.283
7.524
7.507
7.486
7.470
7.412
7.398
7.336
7.320
7.304
7.261
7.048
7.033
4.778
3.409
3.397
3.384
2.083
2.077
2.069
2.063
2.056
Parameter Value
1 Comment liysh-3-195-ol-1H
2 Origin Bruker BioSpin GmbH
3 Instrument spect
4 Solvent CDCl3
5 Temperature 295.3
6 Pulse Sequence zg30
7 Experiment 1D
8 Probe Z119470_0274 (PA BBO 500S1 BBF-H-D-05 Z SP)
9 Number of Scans 14
10 Receiver Gain 21.1
11 Relaxation Delay 1.0000
12 Pulse Width 10.0000
13 Presaturation Frequency
14 Acquisition Time 2.1845
15 Acquisition Date 2020-09-12T21:35:52
16 Modification Date 2020-09-12T21:35:52
17 Spectrometer Frequency 500.13
18 Spectral Width 7500.0
19 Lowest Frequency -761.8
20 Nucleus 1H
21 Acquired Size 16384
22 Spectral Size 65536
N
OH
0.95
5.82
1.01
1.03
0.99
1.98
4.00
4.08
f1 (ppm)

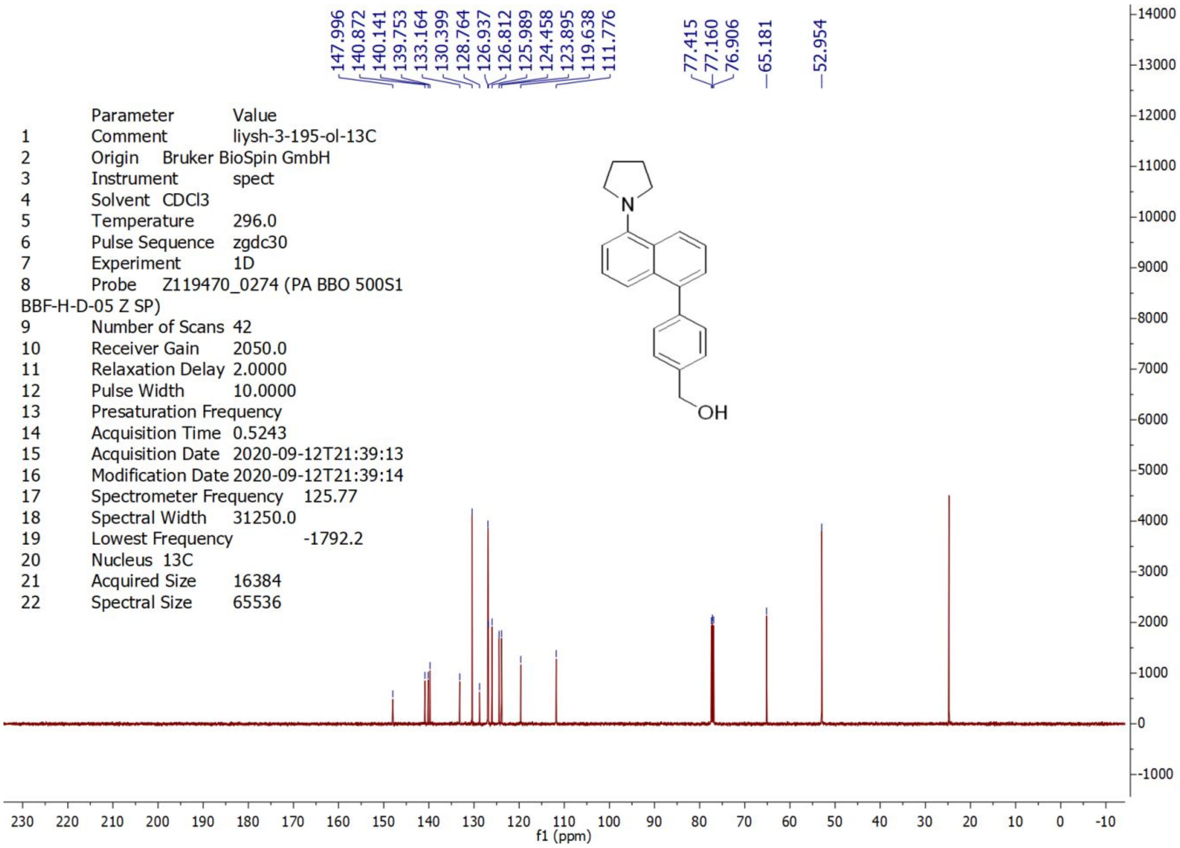
147.996
140.872
140.141
139.753
133.164
130.399
128.764
126.937
126.812
125.989
124.458
123.895
119.638
111.776
77.415
77.160
76.906
65.181
52.954
Parameter Value
1 Comment liysh-3-195-ol-13C
2 Origin Bruker BioSpin GmbH
3 Instrument spect
4 Solvent CDCl3
5 Temperature 296.0
6 Pulse Sequence zgdc30
7 Experiment 1D
8 Probe Z119470_0274 (PA BBO 500S1 BBF-H-D-05 Z SP)
9 Number of Scans 42
10 Receiver Gain 2050.0
11 Relaxation Delay 2.0000
12 Pulse Width 10.0000
13 Presaturation Frequency
14 Acquisition Time 0.5243
15 Acquisition Date 2020-09-12T21:39:13
16 Modification Date 2020-09-12T21:39:14
17 Spectrometer Frequency 125.77
18 Spectral Width 31250.0
19 Lowest Frequency -1792.2
20 Nucleus 13C
21 Acquired Size 16384
22 Spectral Size 65536
N
OH
f1 (ppm)

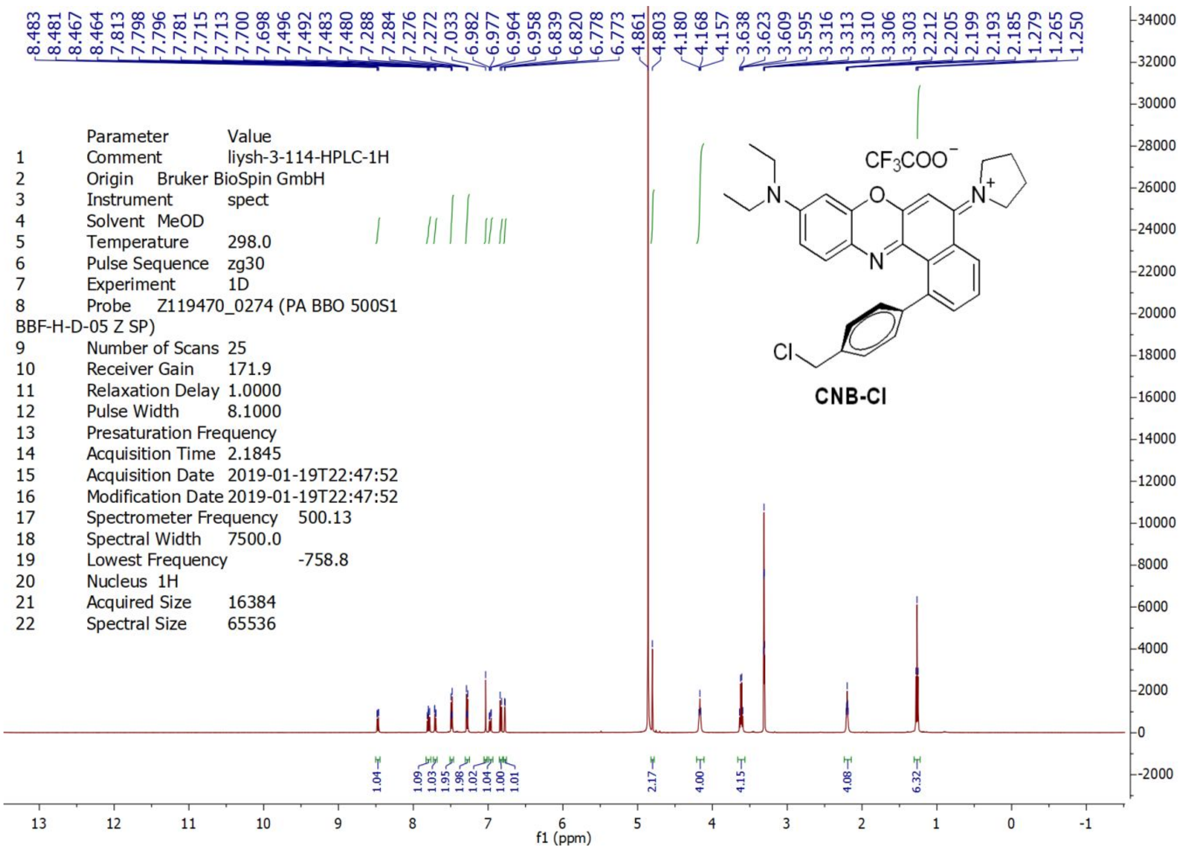
8.483
8.481
8.467
8.464
7.813
7.798
7.796
7.781
7.715
7.713
7.700
7.698
7.496
7.492
7.483
7.480
7.288
7.284
7.276
7.272
7.033
6.982
6.977
6.964
6.958
6.839
6.820
6.778
6.773
4.861
4.803
4.180
4.168
4.157
3.638
3.623
3.609
3.595
3.316
3.313
3.310
3.306
3.303
2.212
2.205
2.199
2.193
2.185
1.279
1.265
1.250
Parameter Value
1 Comment liysh-3-114-HPLC-1H
2 Origin Bruker BioSpin GmbH
3 Instrument spect
4 Solvent MeOD
5 Temperature 298.0
6 Pulse Sequence zg30
7 Experiment 1D
8 Probe Z119470_0274 (PA BBO 500S1 BBF-H-D-05 Z SP)
9 Number of Scans 25
10 Receiver Gain 171.9
11 Relaxation Delay 1.0000
12 Pulse Width 8.1000
13 Presaturation Frequency
14 Acquisition Time 2.1845
15 Acquisition Date 2019-01-19T22:47:52
16 Modification Date 2019-01-19T22:47:52
17 Spectrometer Frequency 500.13
18 Spectral Width 7500.0
19 Lowest Frequency -758.8
20 Nucleus 1H
21 Acquired Size 16384
22 Spectral Size 65536
CF3COO−
N
O
N+
N
Cl
CNB-Cl
1.04
1.09
1.03
1.95
1.98
1.02
1.04
1.00
1.01
2.17
4.00
4.15
4.08
6.32
f1 (ppm)

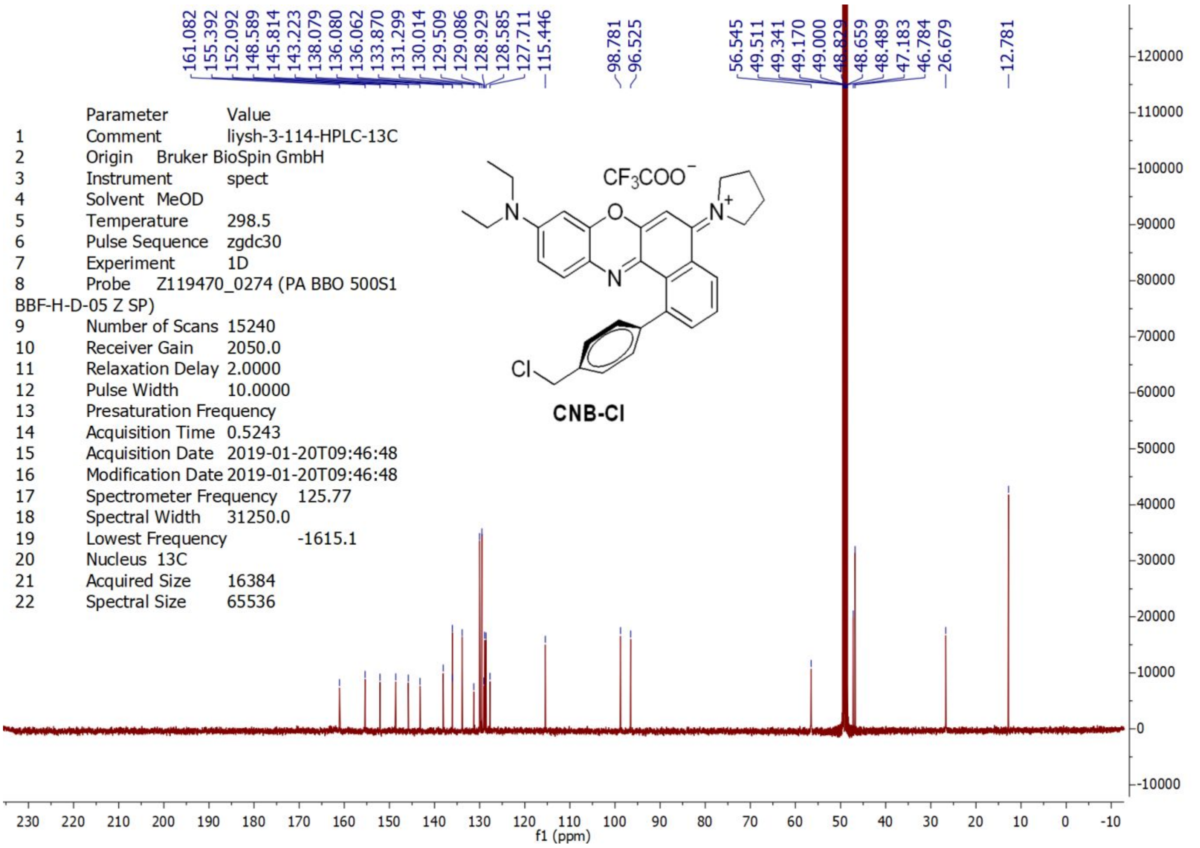
161.082
155.392
152.092
148.589
145.814
143.223
138.079
136.080
136.062
133.870
131.299
130.014
129.509
129.086
128.929
128.585
127.711
115.446
98.781
96.525
56.545
49.511
49.341
49.170
49.000
48.829
48.659
48.489
47.183
46.784
26.679
12.781
Parameter Value
1 Comment liysh-3-114-HPLC-13C
2 Origin Bruker BioSpin GmbH
3 Instrument spect
4 Solvent MeOD
5 Temperature 298.5
6 Pulse Sequence zgdc30
7 Experiment 1D
8 Probe Z119470_0274 (PA BBO 500S1 BBF-H-D-05 Z SP)
9 Number of Scans 15240
10 Receiver Gain 2050.0
11 Relaxation Delay 2.0000
12 Pulse Width 10.0000
13 Presaturation Frequency
14 Acquisition Time 0.5243
15 Acquisition Date 2019-01-20T09:46:48
16 Modification Date 2019-01-20T09:46:48
17 Spectrometer Frequency 125.77
18 Spectral Width 31250.0
19 Lowest Frequency -1615.1
20 Nucleus 13C
21 Acquired Size 16384
22 Spectral Size 65536
CF3COO⁻
N
O
N+
N
Cl
CNB-Cl
f1 (ppm)

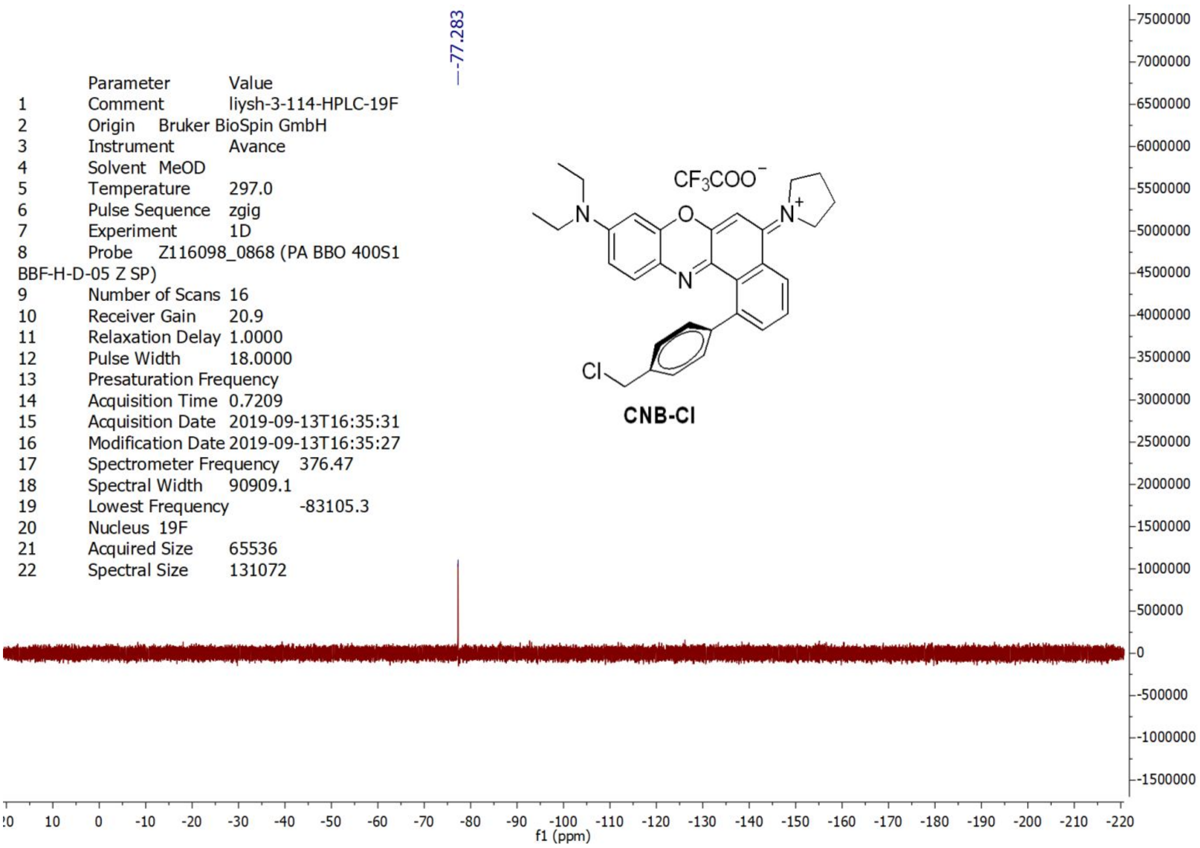
-77.283
Parameter Value
1 Comment liysh-3-114-HPLC-19F
2 Origin Bruker BioSpin GmbH
3 Instrument Avance
4 Solvent MeOD
5 Temperature 297.0
6 Pulse Sequence zgig
7 Experiment 1D
8 Probe Z116098_0868 (PA BBO 400S1 BBF-H-D-05 Z SP)
9 Number of Scans 16
10 Receiver Gain 20.9
11 Relaxation Delay 1.0000
12 Pulse Width 18.0000
13 Presaturation Frequency
14 Acquisition Time 0.7209
15 Acquisition Date 2019-09-13T16:35:31
16 Modification Date 2019-09-13T16:35:27
17 Spectrometer Frequency 376.47
18 Spectral Width 90909.1
19 Lowest Frequency -83105.3
20 Nucleus 19F
21 Acquired Size 65536
22 Spectral Size 131072
CF3COO⁻
N
O
N+
N
Cl
CNB-Cl
f1 (ppm)

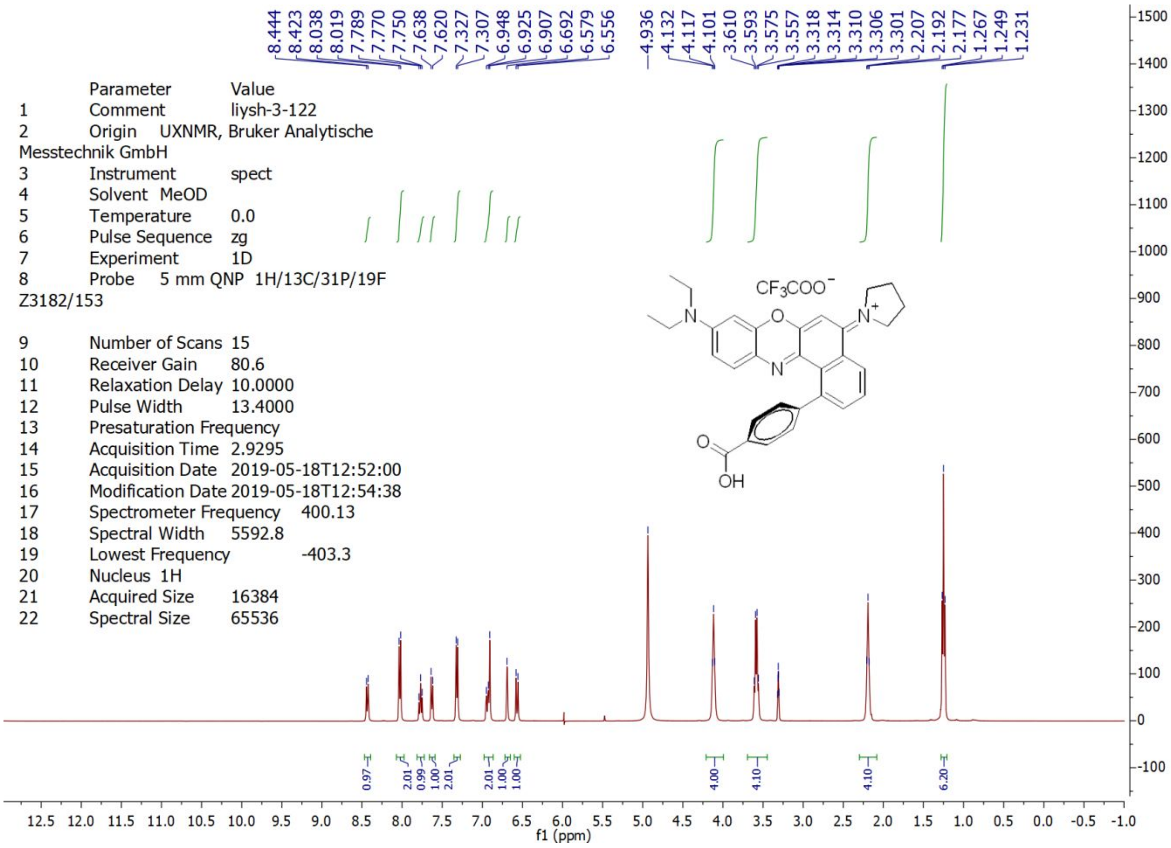
8.444 8.423 8.038 8.019 7.789 7.770 7.750 7.638 7.620 7.327 7.307 6.948 6.925 6.907 6.692 6.579 6.556 4.936 4.132 4.117 4.101 3.610 3.593 3.575 3.557 3.318 3.314 3.310 3.306 3.301 2.207 2.192 2.177 1.267 1.249 1.231
Parameter Value
1 Comment liysh-3-122
2 Origin UXNMR, Bruker Analytische Messtechnik GmbH
3 Instrument spect
4 Solvent MeOD
5 Temperature 0.0
6 Pulse Sequence zg
7 Experiment 1D
8 Probe 5 mm QNP 1H/13C/31P/19F Z3182/153
9 Number of Scans 15
10 Receiver Gain 80.6
11 Relaxation Delay 10.0000
12 Pulse Width 13.4000
13 Presaturation Frequency
14 Acquisition Time 2.9295
15 Acquisition Date 2019-05-18T12:52:00
16 Modification Date 2019-05-18T12:54:38
17 Spectrometer Frequency 400.13
18 Spectral Width 5592.8
19 Lowest Frequency -403.3
20 Nucleus 1H
21 Acquired Size 16384
22 Spectral Size 65536
CF3COO−
N+
N
O
OH
0.97 2.01 0.99 1.00 2.01 2.01 1.00 1.00 4.00 4.10 4.10 6.20
f1 (ppm)

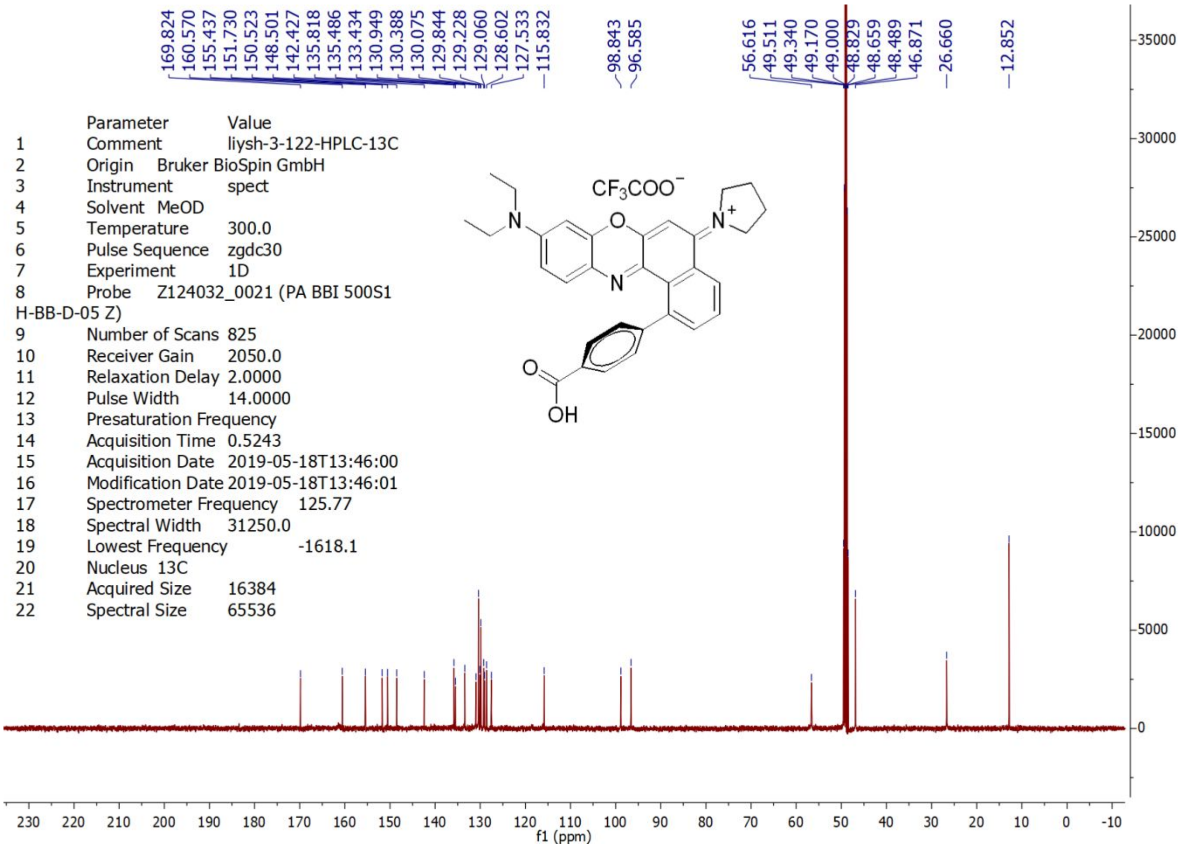
169.824 160.570 155.437 151.730 150.523 148.501 142.427 135.818 135.486 133.434 130.949 130.388 130.075 129.844 129.228 129.060 128.602 127.533 115.832 98.843 96.585 56.616 49.511 49.340 49.170 49.000 48.829 48.659 48.489 46.871 26.660 12.852
Parameter Value
1 Comment liysh-3-122-HPLC-13C
2 Origin Bruker BioSpin GmbH
3 Instrument spect
4 Solvent MeOD
5 Temperature 300.0
6 Pulse Sequence zgdc30
7 Experiment 1D
8 Probe Z124032_0021 (PA BBI 500S1 H-BB-D-05 Z)
9 Number of Scans 825
10 Receiver Gain 2050.0
11 Relaxation Delay 2.0000
12 Pulse Width 14.0000
13 Presaturation Frequency
14 Acquisition Time 0.5243
15 Acquisition Date 2019-05-18T13:46:00
16 Modification Date 2019-05-18T13:46:01
17 Spectrometer Frequency 125.77
18 Spectral Width 31250.0
19 Lowest Frequency -1618.1
20 Nucleus 13C
21 Acquired Size 16384
22 Spectral Size 65536
CF3COO−
N+
N
O
OH
f1 (ppm)

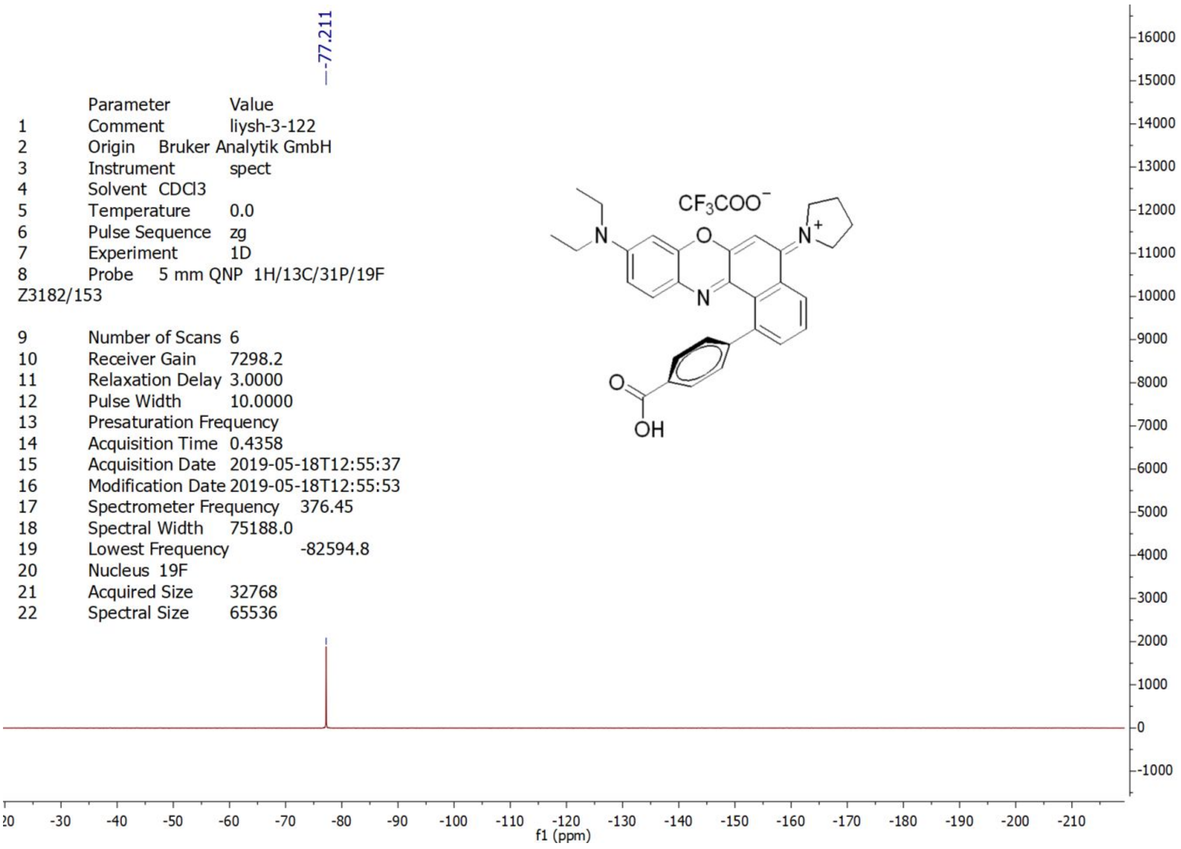
-77.211
Parameter Value
1 Comment liysh-3-122
2 Origin Bruker Analytik GmbH
3 Instrument spect
4 Solvent CDCl3
5 Temperature 0.0
6 Pulse Sequence zg
7 Experiment 1D
8 Probe 5 mm QNP 1H/13C/31P/19F Z3182/153
9 Number of Scans 6
10 Receiver Gain 7298.2
11 Relaxation Delay 3.0000
12 Pulse Width 10.0000
13 Presaturation Frequency
14 Acquisition Time 0.4358
15 Acquisition Date 2019-05-18T12:55:37
16 Modification Date 2019-05-18T12:55:53
17 Spectrometer Frequency 376.45
18 Spectral Width 75188.0
19 Lowest Frequency -82594.8
20 Nucleus 19F
21 Acquired Size 32768
22 Spectral Size 65536
CF3COO⁻
f1 (ppm)

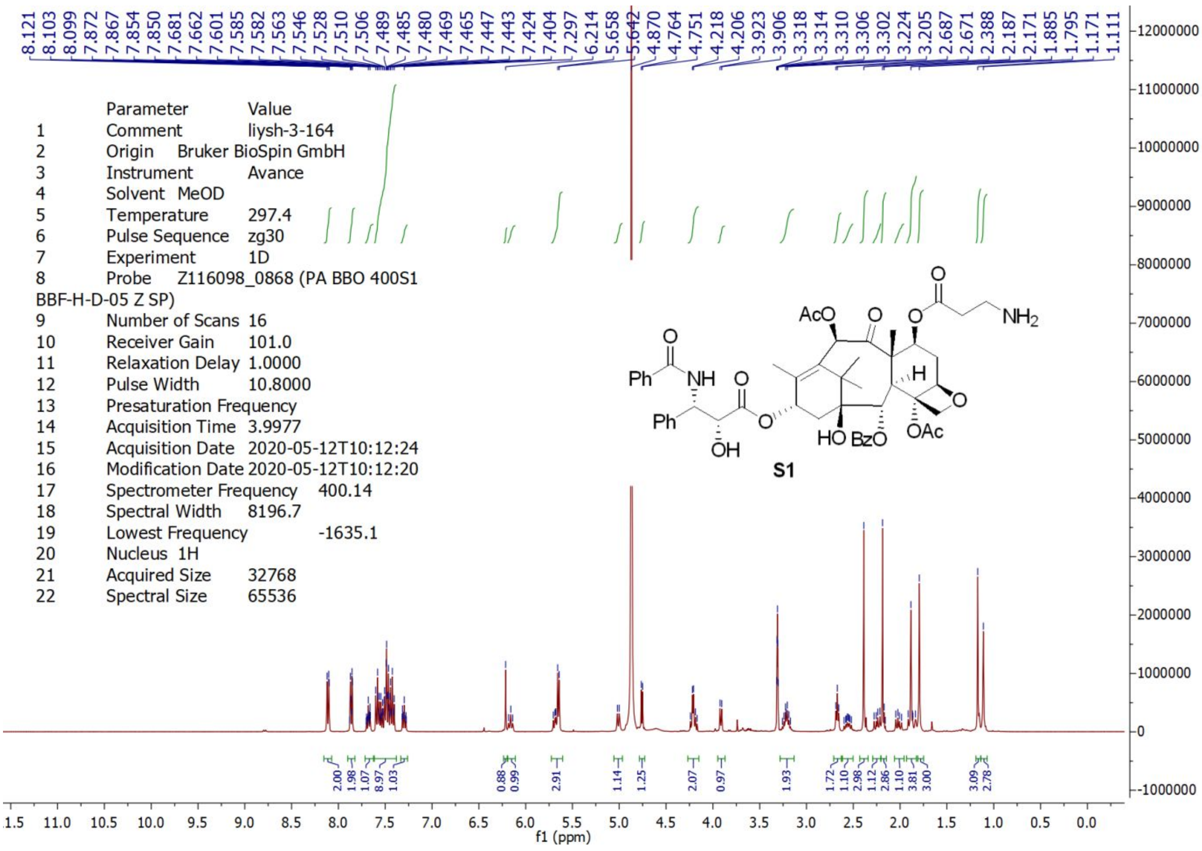
8.121 8.103 8.099 7.872 7.867 7.854 7.850 7.681 7.662 7.601 7.585 7.582 7.563 7.546 7.528 7.510 7.506 7.489 7.485 7.480 7.469 7.465 7.447 7.443 7.424 7.404 7.297 6.214 5.658 5.642 4.870 4.764 4.751 4.218 4.206 3.923 3.906 3.318 3.314 3.310 3.306 3.302 3.224 3.205 2.687 2.671 2.388 2.187 2.171 1.885 1.795 1.171 1.111
Parameter Value
1 Comment liysh-3-164
2 Origin Bruker BioSpin GmbH
3 Instrument Avance
4 Solvent MeOD
5 Temperature 297.4
6 Pulse Sequence zg30
7 Experiment 1D
8 Probe Z116098_0868 (PA BBO 400S1 BBF-H-D-05 Z SP)
9 Number of Scans 16
10 Receiver Gain 101.0
11 Relaxation Delay 1.0000
12 Pulse Width 10.8000
13 Presaturation Frequency
14 Acquisition Time 3.9977
15 Acquisition Date 2020-05-12T10:12:24
16 Modification Date 2020-05-12T10:12:20
17 Spectrometer Frequency 400.14
18 Spectral Width 8196.7
19 Lowest Frequency -1635.1
20 Nucleus 1H
21 Acquired Size 32768
22 Spectral Size 65536
S1
f1 (ppm)

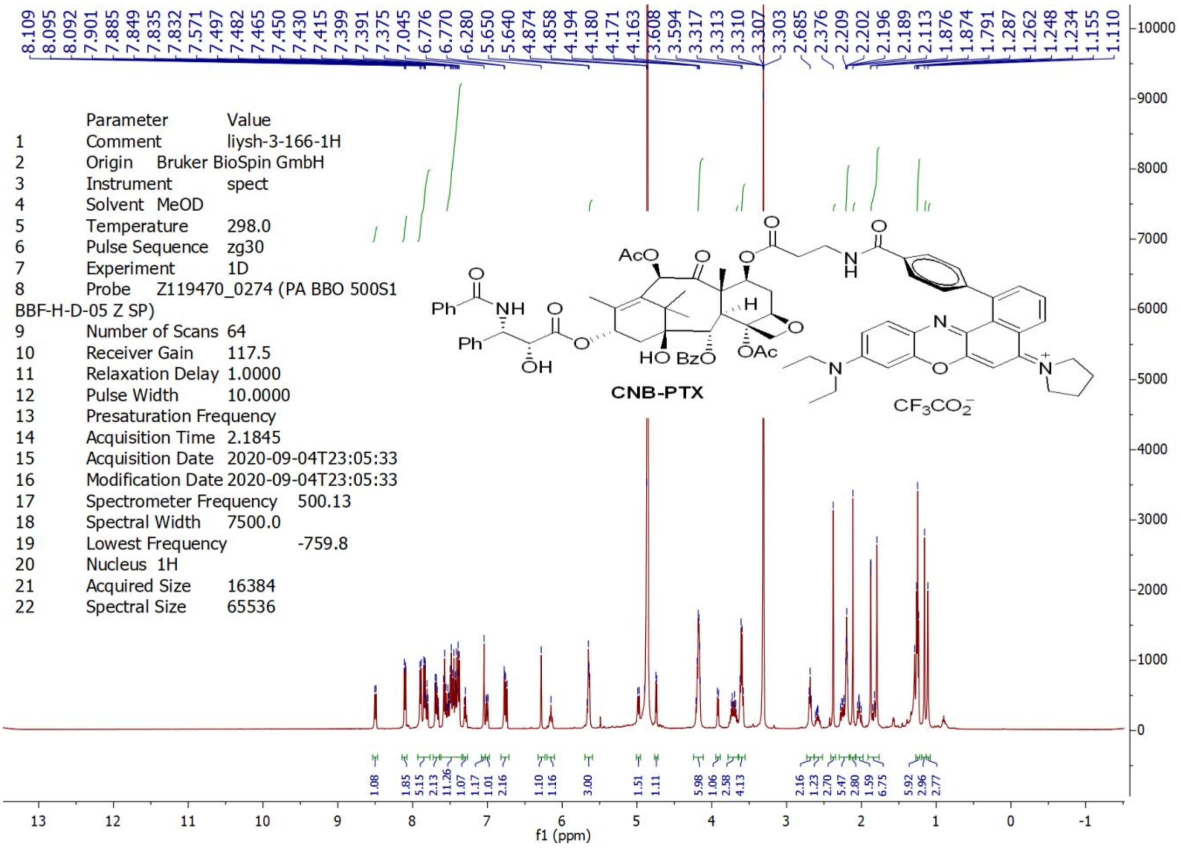
8.109 8.095 8.092 7.901 7.885 7.849 7.835 7.832 7.571 7.497 7.482 7.465 7.450 7.430 7.415 7.399 7.391 7.375 7.045 6.776 6.770 6.280 5.650 5.640 4.874 4.858 4.194 4.180 4.171 4.163 3.608 3.594 3.317 3.313 3.310 3.307 3.303 2.685 2.376 2.209 2.202 2.196 2.189 2.113 1.876 1.874 1.791 1.287 1.262 1.248 1.234 1.155 1.110
Parameter Value
1 Comment liysh-3-166-1H
2 Origin Bruker BioSpin GmbH
3 Instrument spect
4 Solvent MeOD
5 Temperature 298.0
6 Pulse Sequence zg30
7 Experiment 1D
8 Probe Z119470_0274 (PA BBO 500S1 BBF-H-D-05 Z SP)
9 Number of Scans 64
10 Receiver Gain 117.5
11 Relaxation Delay 1.0000
12 Pulse Width 10.0000
13 Presaturation Frequency
14 Acquisition Time 2.1845
15 Acquisition Date 2020-09-04T23:05:33
16 Modification Date 2020-09-04T23:05:33
17 Spectrometer Frequency 500.13
18 Spectral Width 7500.0
19 Lowest Frequency -759.8
20 Nucleus 1H
21 Acquired Size 16384
22 Spectral Size 65536
CNB-PTX
f1 (ppm)

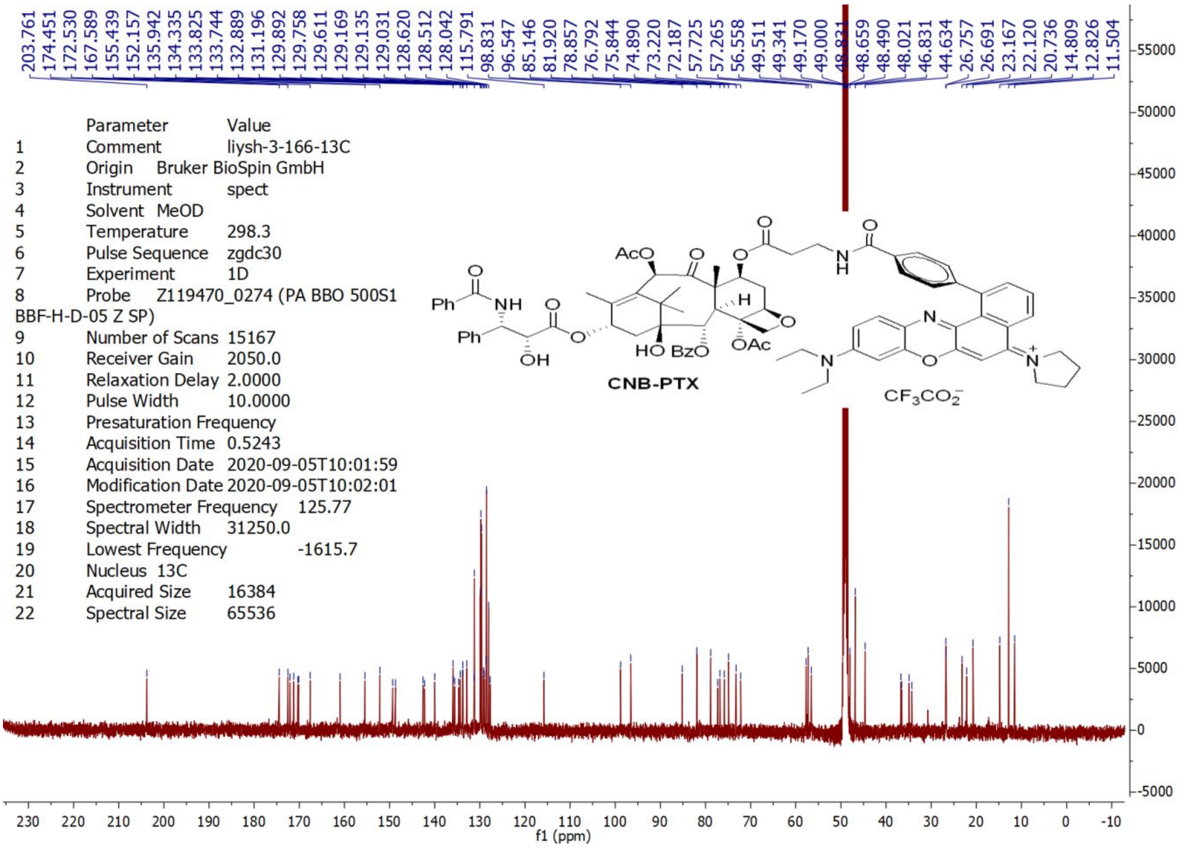
203.761 174.451 172.530 167.589 165.439 152.157 135.942 134.335 133.825 133.744 132.889 131.196 129.892 129.758 129.611 129.169 129.135 129.031 128.620 128.512 128.042 115.791 98.831 96.547 85.146 81.920 78.857 76.792 75.844 74.890 73.220 72.187 57.725 57.265 56.558 49.511 49.341 49.170 49.000 48.831 48.659 48.490 48.021 46.831 44.634 26.757 26.691 23.167 22.120 20.736 14.809 12.826 11.504
Parameter Value
1 Comment liysh-3-166-13C
2 Origin Bruker BioSpin GmbH
3 Instrument spect
4 Solvent MeOD
5 Temperature 298.3
6 Pulse Sequence zgdc30
7 Experiment 1D
8 Probe Z119470_0274 (PA BBO 500S1 BBF-H-D-05 Z SP)
9 Number of Scans 15167
10 Receiver Gain 2050.0
11 Relaxation Delay 2.0000
12 Pulse Width 10.0000
13 Presaturation Frequency
14 Acquisition Time 0.5243
15 Acquisition Date 2020-09-05T10:01:59
16 Modification Date 2020-09-05T10:02:01
17 Spectrometer Frequency 125.77
18 Spectral Width 31250.0
19 Lowest Frequency -1615.7
20 Nucleus 13C
21 Acquired Size 16384
22 Spectral Size 65536
CNB-PTX
f1 (ppm)

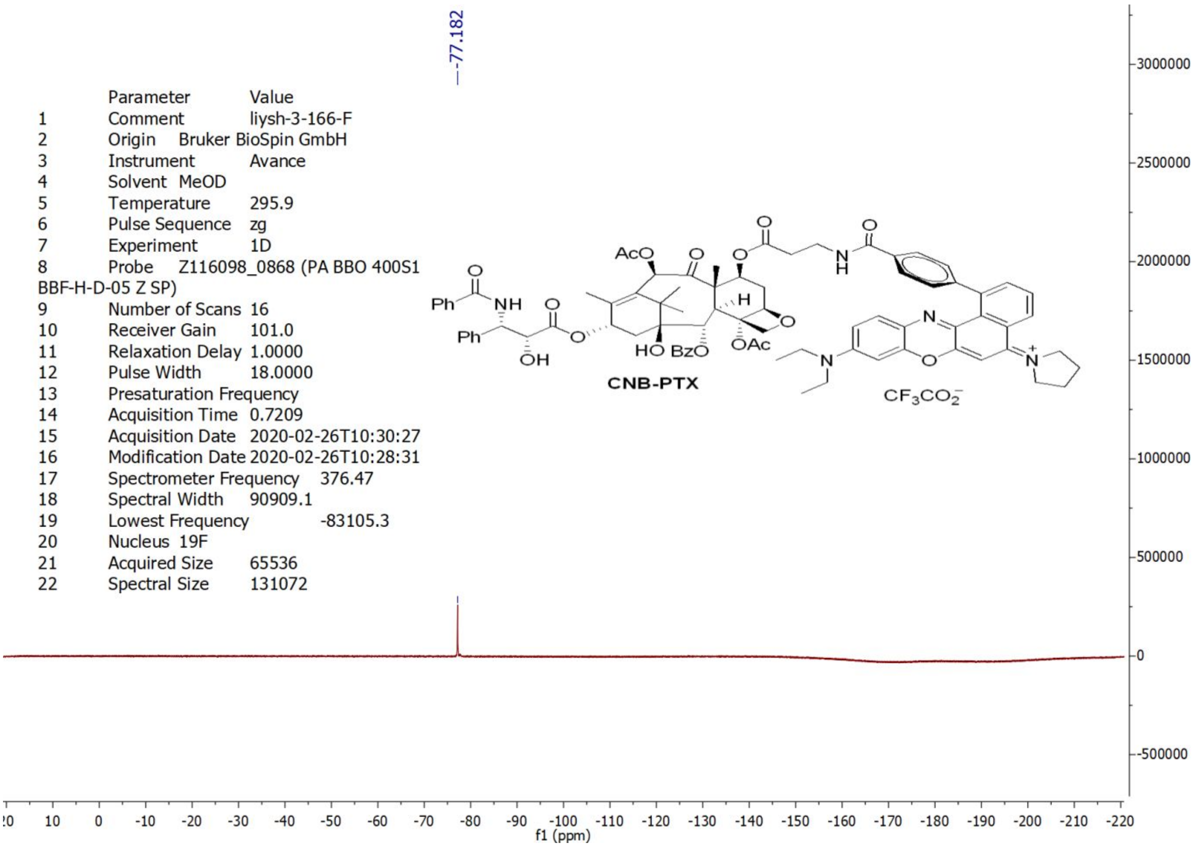
-77.182
Parameter Value
1 Comment liysh-3-166-F
2 Origin Bruker BioSpin GmbH
3 Instrument Avance
4 Solvent MeOD
5 Temperature 295.9
6 Pulse Sequence zg
7 Experiment 1D
8 Probe Z116098_0868 (PA BBO 400S1 BBF-H-D-05 Z SP)
9 Number of Scans 16
10 Receiver Gain 101.0
11 Relaxation Delay 1.0000
12 Pulse Width 18.0000
13 Presaturation Frequency
14 Acquisition Time 0.7209
15 Acquisition Date 2020-02-26T10:30:27
16 Modification Date 2020-02-26T10:28:31
17 Spectrometer Frequency 376.47
18 Spectral Width 90909.1
19 Lowest Frequency -83105.3
20 Nucleus 19F
21 Acquired Size 65536
22 Spectral Size 131072
CNB-PTX
$CF_3CO_2^-$
f1 (ppm)

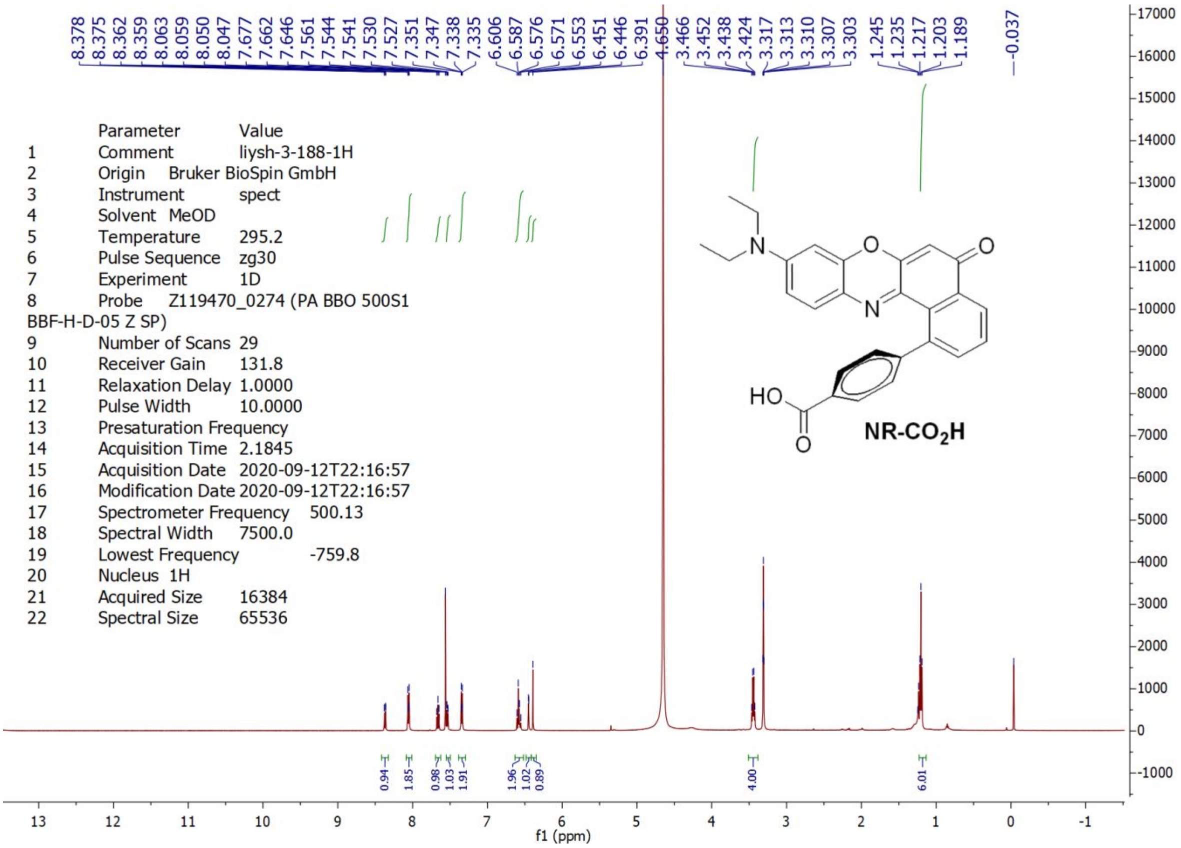
8.378 8.375 8.362 8.359 8.063 8.059 8.050 8.047 7.677 7.662 7.646 7.561 7.544 7.541 7.530 7.527 7.351 7.347 7.338 7.335 6.606 6.587 6.576 6.571 6.553 6.451 6.446 6.391 4.650 3.466 3.452 3.438 3.424 3.317 3.313 3.310 3.307 3.303 1.245 1.235 1.217 1.203 1.189 -0.037
Parameter Value
1 Comment liysh-3-188-1H
2 Origin Bruker BioSpin GmbH
3 Instrument spect
4 Solvent MeOD
5 Temperature 295.2
6 Pulse Sequence zg30
7 Experiment 1D
8 Probe Z119470_0274 (PA BBO 500S1 BBF-H-D-05 Z SP)
9 Number of Scans 29
10 Receiver Gain 131.8
11 Relaxation Delay 1.0000
12 Pulse Width 10.0000
13 Presaturation Frequency
14 Acquisition Time 2.1845
15 Acquisition Date 2020-09-12T22:16:57
16 Modification Date 2020-09-12T22:16:57
17 Spectrometer Frequency 500.13
18 Spectral Width 7500.0
19 Lowest Frequency -759.8
20 Nucleus 1H
21 Acquired Size 16384
22 Spectral Size 65536
$NR-CO_2H$
0.94 1.85 0.98 1.03 1.91 1.96 1.02 0.89 4.00 6.01
f1 (ppm)

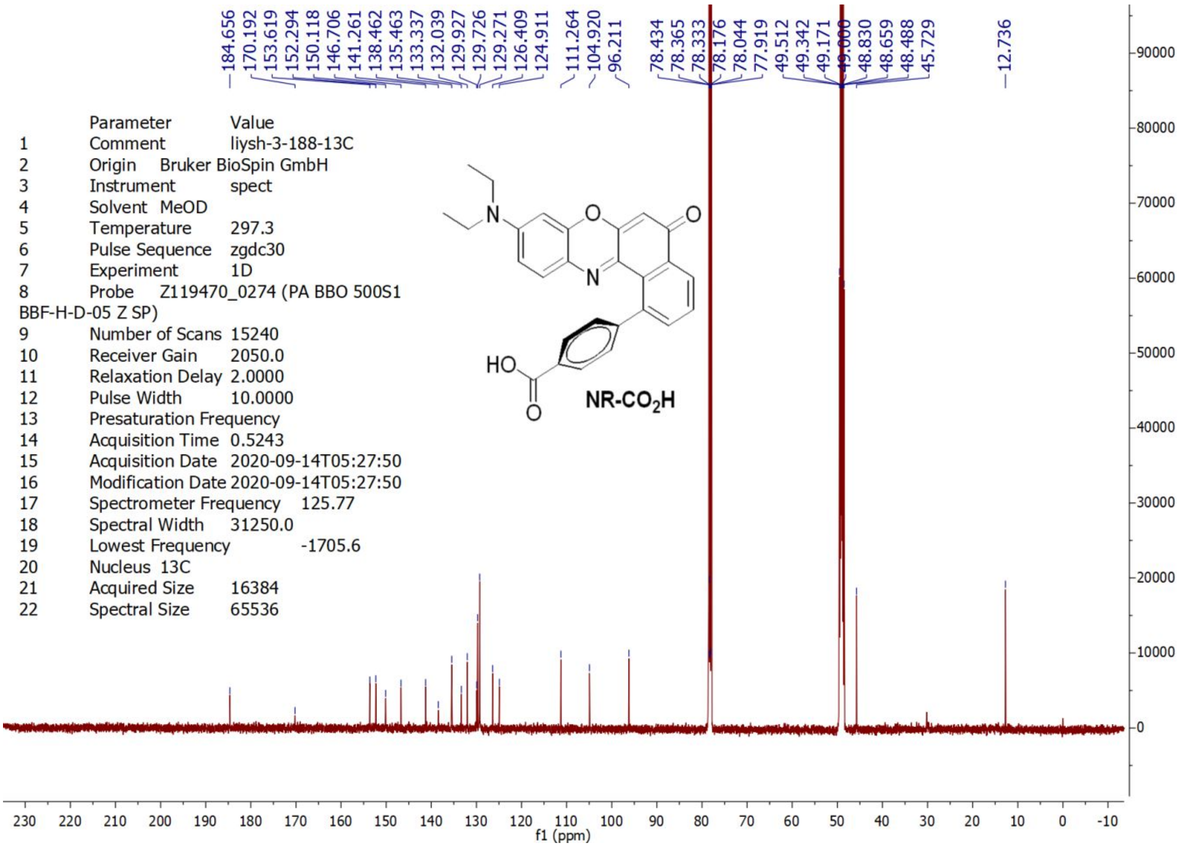
184.656 170.192 153.619 152.294 150.118 146.706 141.261 138.462 135.463 133.337 132.039 129.927 129.726 129.271 126.409 124.911 111.264 104.920 96.211 78.434 78.365 78.333 78.176 78.044 77.919 49.512 49.342 49.171 49.000 48.830 48.659 48.488 45.729 12.736
Parameter Value
1 Comment liysh-3-188-13C
2 Origin Bruker BioSpin GmbH
3 Instrument spect
4 Solvent MeOD
5 Temperature 297.3
6 Pulse Sequence zgdc30
7 Experiment 1D
8 Probe Z119470_0274 (PA BBO 500S1 BBF-H-D-05 Z SP)
9 Number of Scans 15240
10 Receiver Gain 2050.0
11 Relaxation Delay 2.0000
12 Pulse Width 10.0000
13 Presaturation Frequency
14 Acquisition Time 0.5243
15 Acquisition Date 2020-09-14T05:27:50
16 Modification Date 2020-09-14T05:27:50
17 Spectrometer Frequency 125.77
18 Spectral Width 31250.0
19 Lowest Frequency -1705.6
20 Nucleus 13C
21 Acquired Size 16384
22 Spectral Size 65536
NR-CO2H
f1 (ppm)

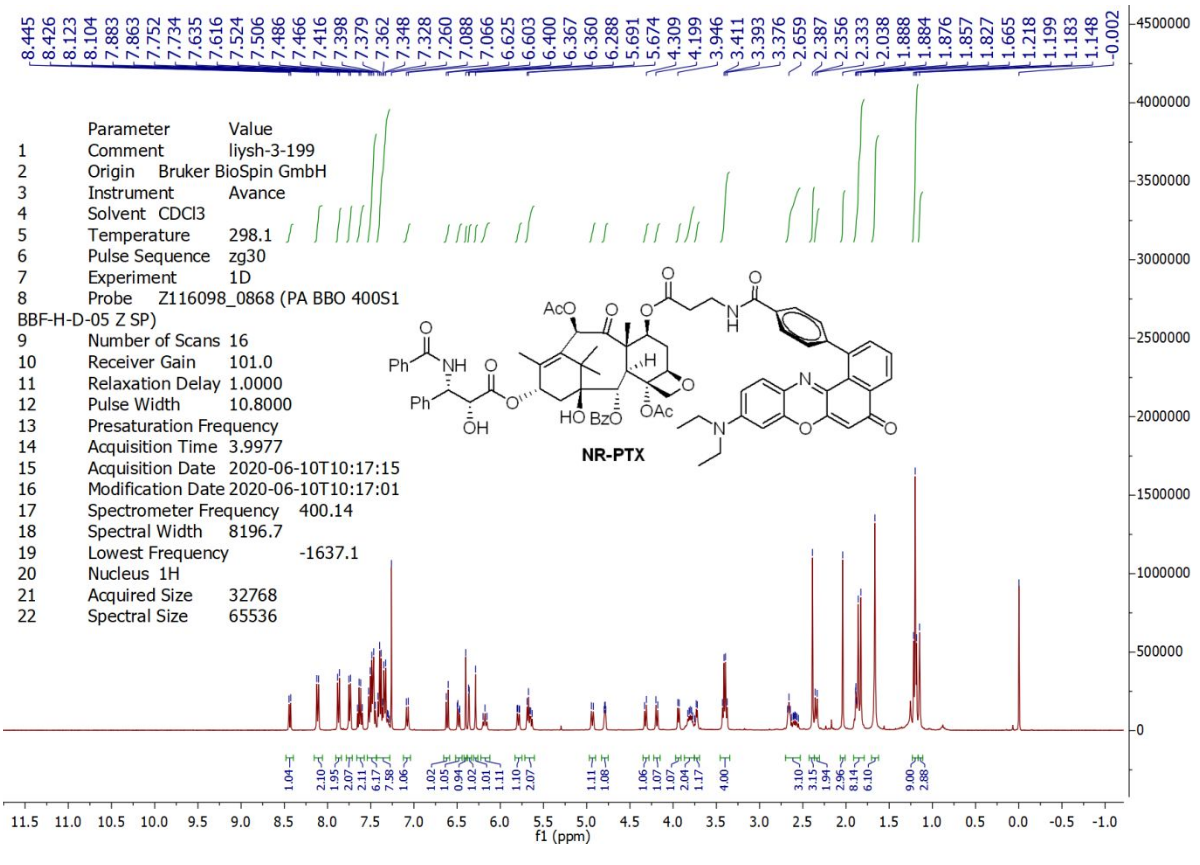
8.445 8.426 8.123 8.104 7.883 7.863 7.752 7.734 7.635 7.616 7.524 7.506 7.486 7.466 7.416 7.398 7.379 7.362 7.348 7.328 7.260 7.088 7.066 6.625 6.603 6.400 6.367 6.360 6.288 5.691 5.674 4.309 4.199 3.946 3.411 3.393 3.376 2.659 2.387 2.356 2.333 2.038 1.888 1.884 1.876 1.857 1.827 1.665 1.218 1.199 1.183 1.148 -0.002
Parameter Value
1 Comment liysh-3-199
2 Origin Bruker BioSpin GmbH
3 Instrument Avance
4 Solvent CDCl3
5 Temperature 298.1
6 Pulse Sequence zg30
7 Experiment 1D
8 Probe Z116098_0868 (PA BBO 400S1 BBF-H-D-05 Z SP)
9 Number of Scans 16
10 Receiver Gain 101.0
11 Relaxation Delay 1.0000
12 Pulse Width 10.8000
13 Presaturation Frequency
14 Acquisition Time 3.9977
15 Acquisition Date 2020-06-10T10:17:15
16 Modification Date 2020-06-10T10:17:01
17 Spectrometer Frequency 400.14
18 Spectral Width 8196.7
19 Lowest Frequency -1637.1
20 Nucleus 1H
21 Acquired Size 32768
22 Spectral Size 65536
NR-PTX
f1 (ppm)

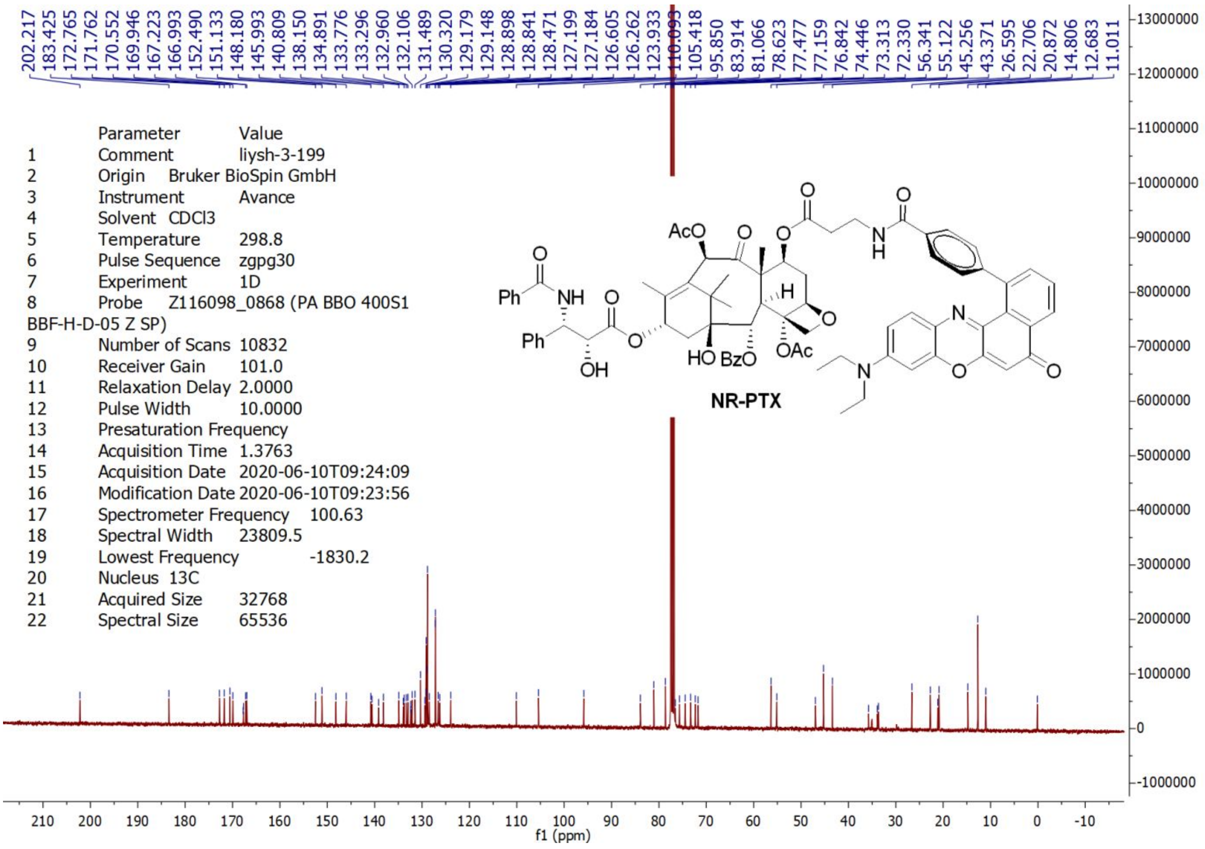

8. References

(1) Martin-Brown, S. A.; Fu, Y.; Saroja, G.; Collinson, M. M.; Higgins, D. A. Single-molecule studies of diffusion by oligomer-bound dyes in organically modified sol-gel-derived silicate films. *Anal. Chem.* **2005**, *77* (2), 486-494.

(2) Guy, R.; Scott, Z.; Sloboda, R.; Nicolaou, K. Fluorescent taxoids. *Chem Biol* **1996**, *3* (12), 1021-1031.

(3) Sens, R.; Drexhage, K. H. Fluorescence quantum yield of oxazine and carbazine laser dyes. *J. Lumin.* **1981**, *24/25*, 709-712.

(4) Marsh, R. J.; Pfisterer, K.; Bennett, P.; Hirvonen, L. M.; Gautel, M.; Jones, G. E.; Cox, S. Artifact-free high-density localization microscopy analysis. *Nat. Methods* **2018**, *15* (9), 689-692.

(5) Ovesny, M.; Krizek, P.; Borkovec, J.; Svindrych, Z.; Hagen, G. M. ThunderSTORM: a comprehensive ImageJ plug-in for PALM and STORM data analysis and super-resolution imaging. *Bioinformatics* **2014**, *30* (16), 2389-2390.
