# Supplementary material for: Development and Application of Cationic Nile Blue Probes in Live-Cell Super-Resolution Imaging and Specific Targeting to Mitochondria
Source: ACS Cent Sci. 2024 May 17;10(6):1221–30. doi: 10.1021/acscentsci.4c00073 (PMC11212141; doi:10.1021/acscentsci.4c00073)
Supplement: Supplementary file 6 — oc4c00073_si_006.pdf [file oc4c00073_si_006.pdf]

oc-2024-00073y.R1

Name: Peer Review Information for "Development and application of cationic Nile blue probes in live-cell super-resolution imaging and specific targeting to mitochondria"

First Round of Reviewer Comments

Reviewer: 1

Comments to the Author

In this paper, authors developed an unique cationic Nile blue, for mitochondrial

imaging and targeting. These dyes possess high cell permeability, excellent mitochondrial specificity, near-infrared (NIR) emission, solvatochromism, and good stability

towards ROS. These attributes make them valuable additions to the existing repertoire of dyes. The probes can be

used at low dosages for imaging mitochondria with an excellent signal-to-noise ratio in both live cells and live

worms. The preparation procedure for CNB is concise, allowing for the rapid synthesis of analogs for future development. Moreover, the reversible redox-switching behavior of CNB-Cl enables SMLM imaging of mitochondria,

as well as the visualization of their fusion-fission dynamics

with sub-diffraction-limit resolution under mild conditions.

Finally, we have extended the application of cationic Nile

blue to the mitochondria-specific delivery of taxanes, triggering cell death. We believe that these novel cationic Nile

blue probes, with their promising properties, can enhance

the toolbox of super-resolution imaging techniques and facilitate discoveries in mitochondrial biology and drug resistance mechanisms.

ASSOCIATED CONTENT

Supporting Information. Supporting Information i

Reviewer: 2

Comments to the Author

Comments:

This manuscript reported the design, synthesis, and application of cationic Nile blue dyes for live-cell super-resolution imaging and selective targeting of mitochondria. They have utilized these photostable probes in single-molecule localization microscopy of mitochondria to detect mitochondrial fission and fusion behaviors. Moreover, they have exploited these cationic Nile blue probes to the mitochondria specific delivery of anti-cancer drugs, such as taxanes, and scrutinized the interactions between the drug and organelles. This work presented some interesting results and the data almost supported the authors' claim. I would like to recommend the publication of this manuscript in ACS Central Science after major revisions. Numerous sections of the work need to be clarified and improved.

a) Authors should perform mitochondrial dynamics using their synthesized cationic Nile blue probes in another two cell lines using single-molecule localization microscopy.

b) Mitochondrial fission and fusion images are not of high quality. Cristae structure of mitochondria is not clear. Authors should provide better images of mitochondrial fission and fusion including cristae structure. Authors should also provide super resolution videos of mitochondrial fission and fusion.

c) Is the probe selective for the cancerous cell mitochondria compared to normal cell mitochondria? In case of the mitochondria specific delivery of taxanes, is it accumulating both the cancerous and noncancerous cells or it is selective for cancer cells.

d) The quantum yield of the synthesized dyes should be reported.

e) Stability of the dyes in presence of various bio-analytes (probable interfering analytes from biological milieu) should be performed.

f) In supporting information, page 27, bottom Figure, 1H NMR in CDCl3 the peak at 4.015 ppm is not assigned. It is for the compound peak -COOMe. Please integrate the peak.

g) In supporting information, page 28, 1H NMR in CDCl3, the peak at 4.711 ppm is not assigned. It is for the compound peak (benzylic protons). Please integrate the peak.

h) In supporting information, page 29, bottom Figure, 1H NMR in CDCl3, the peak at 4.778 ppm is not assigned. It is for the compound peak (benzylic protons). Please integrate the peak.

i) Please provide Pearson correlation graph for Figures S4, S5, and S6. Please also provide fluorescence line plots for Figures S4, S5, and S6.

j) Please provide a scale bar for the bottom images of Figure S5.

Reviewer: 3

Comments to the Author

The study by Yunsheng Li, Xiaoyu Bai and Dan Yang is a very comprehensive piece of novel tool compound development. The newly synthesized cationic Nile blue probes will aid imaging of mitochondria under regular circumstances but also - as has been applied in the current manuscript - for super-resolution microscopy.

The story is very well carved out - the images are clear and the experiments are interpreted rightly. Additional experiments ascertained that the new compounds do not interfere with some other compound's toxic effects (paclitaxel) - which is an interesting observation but not the most relevant. Because what should have been done for a probe designed for imaging purposes, is to examine whether the novel compounds induce cytotoxicity themselves.

This is an experiment the authors might want to add to increase the usability of the compounds in a broader sense.

Reviewer: 4

Comments to the Author

The manuscript by Li et al describes new fluorescent probes for mitochondria based on Nile Blue dye. It was shown that the optimized Nile Blue derivative can target mitochondria and allow its imaging using conventional and super-resolution microscopy. Moreover, the Nile Blue unit was applied for delivery of drugs into mitochondria and tune the biological effects of the drugs. The manuscript is well written, the conclusions are generally supported by the data and the results will be of broad interest for chemistry community. However, some issues should be clarified before this manuscript can be recommended for publication in ACS Central Science.

1) The comparison of the new probe is done with commercial tracker MTDR, where some advantages of the new probe are shown. However, I am not convinced by this comparison, because MTDR was used at too high concentrations (250-500 nM). Normally, it is supposed to be used at 100 nM concentration, which is significantly lower than that used in the present work. This could explain the observed background in Figure 1I and mitochondrial damage in Figure 1J. To make a fair comparison, the concentration of the probes should be decreased to 100 nM and the experiments should be redone.

2) The effects of PTX drug and its conjugates on mitochondria were not compared at the same concentration. The effect of CNB-PTX were observed at 2 uM, while PTX and PTX-NR were tested at 8 and 250 nM, respectively. One could argue that the changes in the mitochondria could also take place for PTX and PTX-NT at 2 uM concentration. This point should be verified.

3) Minor point, page 3: “In contrast, the absorption/emission profile of Mito-Tracker deep red (MTDR), a specific Nile blue derivative, remains largely unchanged in both PBS and octanol.” This is confusing because MTDR is not a Nile Blue derivative, but a cyanine dye.

Author's Response to Peer Review Comments:

Our point-to-point responses to the reviewers' comments are highlighted in blue color below.

Reviewer 2:

a) Authors should perform mitochondrial dynamics using their synthesized cationic Nile blue probes in another two cell lines using single-molecule localization microscopy.

We appreciate your suggestion to perform mitochondrial dynamics using our synthesized cationic Nile blue probes in two additional cell lines, utilizing Single-Molecule Localization Microscopy (SMLM) for the experiments.

We have conducted these experiments as recommended. The results have been included as Supplementary Video 3, Supplementary Video 4, and Supplementary Figure S16. We believe these additional data further underscore the utility and versatility of our synthesized cationic Nile blue probes in studying mitochondrial dynamics across various cell lines.

b) Mitochondrial fission and fusion images are not of high quality. Cristae structure of mitochondria is not clear. Authors should provide better images of mitochondrial fission and fusion including cristae structure. Authors should also provide super resolution videos of mitochondrial fission and fusion.

Thank you for your feedback regarding the quality of the mitochondrial fission and fusion images and the lack of clarity in the cristae structure of mitochondria. We understand the importance of presenting high-quality, discernible images and videos in our study.

Mitochondrial cristae indeed pose a significant challenge for super-resolution Single-Molecule Localization Microscopy (SMLM) imaging, primarily due to their small size, sensitivity to various stress factors in living cells, and highly dynamic nature. In SMLM, temporal resolution is inevitably sacrificed to enhance spatial resolution. The camera

used in standard SMLM operates at a maximum speed of 100 frames/sec, and approximately 1000 to 2000 frames are typically required to reconstruct a super-resolved image of mitochondria. This implies that the super-resolution image represents an averaged state of mitochondria over a period of 10 to 20 seconds. The motion blur that results can make imaging the cristae of living cells particularly challenging for researchers utilizing SMLM.

However, we have found that alternative imaging techniques with shorter acquisition times, such as Structured Illumination Microscopy (SIM), can capture clear images of cristae using our synthesized cationic Nile blue probes. We aim to disclose these results in a separate publication.

In response to your suggestion, we have added super-resolution videos of mitochondrial fission and fusion as Supplementary Videos 3 and 4.

c) Is the probe selective for the cancerous cell mitochondria compared to normal cell mitochondria? In case of the mitochondria specific delivery of taxanes, is it accumulating both the cancerous and noncancerous cells or it is selective for cancer cells.

Thank you for your insightful questions regarding the selectivity of our probes and their potential for specific delivery of taxanes to mitochondrial targets in cancerous versus noncancerous cells.

Cancerous cells typically exhibit a more negative mitochondrial membrane potential compared to noncancerous cells. In our previous experiments, we observed that when we treated a 1:1 mixture of NIH 3T3 (embryo fibroblast) and HeLa cells with MitoTracker Green (MTG) for 1 hour, there was selective retention of MTG in the mitochondria of HeLa cells, while almost no uptake was observed in NIH 3T3 cells (as shown in the attached figure).

The mechanism for mitochondrial uptake of lipophilic cations, which includes commercial MitoTrackers, cationic Nile blues, and cationic Nile blue-linked taxanes, is believed to be similar; all these compounds penetrate mitochondria by exploiting the large mitochondrial membrane potential. Therefore, it is reasonable to suggest that the probes we developed are selective for cancerous cells. This is further supported by our preliminary results showing that cationic Nile blue-linked taxanes exhibit a much higher IC50 value in NIH3T3 cells compared to HeLa cells.

This strategy is promising, offering an appealing approach to selectively target and annihilate cancerous cells while sparing noncancerous ones. We will continue to investigate this aspect in our future work to confirm and extend these findings.

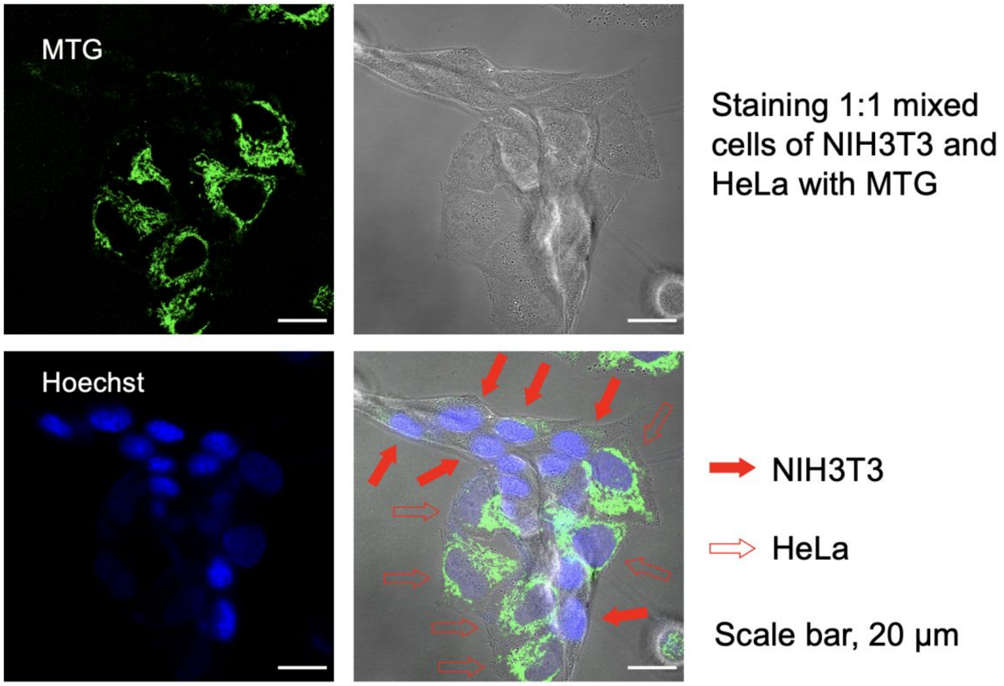

d) The quantum yield of the synthesized dyes should be reported.

Thank you for your comment. We agree that reporting the quantum yield of the synthesized dyes is crucial for understanding their fluorescence efficiency.

In response to your suggestion, we have measured and included the quantum yields of the synthesized dyes in Supplementary Table S1. We believe this additional information will provide a more comprehensive understanding of our synthesized dyes' properties.

e) Stability of the dyes in presence of various bio-analytes (probable interfering analytes from biological milieu) should be performed.

Thank you for your insightful comment regarding the stability of the dyes in the presence of various bio-analytes, including potential interfering analytes from the biological milieu. We agree that this is an important aspect to consider in our study.

We have performed these tests and the results have been included in Figure S4 of the supplementary materials. These findings provide valuable information about the stability of our synthesized dyes under different biological conditions and in the presence of various interfering bio-analytes.

f) In supporting information, page 27, bottom Figure, 1H NMR in CDCl3 the peak at 4.015 ppm is not assigned. It is for the compound peak -COOMe. Please integrate the peak.
g) In supporting information, page 28, 1H NMR in CDCl3, the peak at 4.711 ppm is not assigned. It is for the compound peak (benzylic protons). Please integrate the peak.
h) In supporting information, page 29, bottom Figure, 1H NMR in CDCl3, the peak at 4.778 ppm is not assigned. It is for the compound peak (benzylic protons). Please integrate the peak.

Thank you for your detailed observations and comments regarding the 1H NMR spectra presented in the supporting information. We apologize for the oversight in the initial submission.

a) For the bottom figure on page 27, you are correct that the peak at 4.015 ppm corresponds to the -COOMe compound peak. We have now integrated this peak and updated the figure accordingly.

b) On page 28, the peak at 4.711 ppm indeed corresponds to the benzylic protons. We have integrated this peak and updated the figure as well.

c) Similarly, for the bottom figure on page 29, the peak at 4.778 ppm is for the benzylic protons. This peak has now been integrated.

i) Please provide Pearson correlation graph for Figures S4, S5, and S6. Please also provide fluorescence line plots for Figures S4, S5, and S6.

Thank you for your suggestion to include Pearson correlation graphs and fluorescence line plots for Figures S4, S5, and S6. These additional analyses will indeed provide a more comprehensive understanding of the data presented.

We have now calculated the Pearson correlation and generated the corresponding graphs for Figures S4, S5, and S6. These have been included in the revised Figures S5, S6, and S7, respectively. We have also added the fluorescence line plots in these revised figures.

j) Please provide a scale bar for the bottom images of Figure S5.

Thank you for your suggestion to add a scale bar to the bottom images of Figure S5. We agree that a scale bar is essential for accurately interpreting the size and scale of the images.

We have now added the scale bar to the bottom images of Figure S5 as per your suggestion. We believe this addition will greatly assist in the interpretation of these images.

Reviewer 3:

The study by Yunsheng Li, Xiaoyu Bai and Dan Yang is a very comprehensive piece of novel tool compound development.

The newly synthesized cationic Nile blue probes will aid imaging of mitochondria under regular circumstances but also - as has been applied in the current manuscript - for super-resolution microscopy.

The story is very well carved out - the images are clear and the experiments are interpreted rightly. Additional experiments ascertained that the new compounds do not interfere with some other compound's toxic effects (paclitaxel) - which is an interesting observation but not the most relevant. Because what should have been done for a probe designed for imaging purposes, is to examine whether the novel compounds induce cytotoxicity themselves. This is an experiment the authors might want to add to increase the usability of the compounds in a broader sense.

We appreciate your thoughtful comments and suggestions. You are correct that the potential cytotoxic effects of novel compounds intended for imaging purposes should be thoroughly investigated.

Prior to conducting experiments with Paclitaxel, we were aware that a high volume and prolonged accumulation of lipophilic cations in mitochondria could potentially cause damage to this organelle, and therefore cytotoxicity. As depicted in Figure 1 (J) and (K), we have addressed this concern.

When we observed that the cationic Nile blue-linked Paclitaxel could induce mitochondrial fragmentation, we promptly performed several experiments to understand why this phenomenon occurred. This included comparing the damage effects on mitochondria induced by cationic Nile blue-linked Paclitaxel and the fluorophore alone, as well as the differences in their cellular uptake.

Our results showed that cellular uptake of cationic Nile blue-linked Paclitaxel is significantly lower than that of cationic Nile blue alone. However, the former could induce far more pronounced mitochondrial fragmentation and cytochrome c release, as shown in Figures 3 (J) to 3 (N). Therefore, we concluded that the observed toxic effects are primarily due to the interactions between Paclitaxel and mitochondria, rather than the fluorophore itself.

We believe these findings and elucidations address your concerns and provide a comprehensive understanding of the potential cytotoxic effects of our synthesized cationic Nile blue probes.

Reviewer 4:

1) The comparison of the new probe is done with commercial tracker MTDR, where some advantages of the new probe are shown. However, I am not convinced by this comparison, because MTDR was used at too high concentrations (250-500 nM). Normally, it is supposed to be used at 100 nM concentration, which is significantly lower than that used in the present work. This could explain the observed background in Figure 1I and mitochondrial damage in Figure 1J. To make a fair comparison, the concentration of the probes should be decreased to 100 nM and the experiments should be redone.

Thank you for your insightful comment regarding the comparison of our new probe with the commercial tracker MTDR. We understand your point about the concentration used potentially influencing the observed results.

Your suggestion of reducing the concentration of the probes to 100 nM for a more fair comparison is well-taken. In fact, we did previously try a lower dosage of 100 nM for both MTDR and CNB-Cl in our initial experiments. We observed that the phenomenon of mitochondrial swelling was less significant, but still present, in cells treated with MTDR at this lower concentration. We have now added these results in Figure S12 in the revised manuscript.

Regarding the background fluorescence in Figure 1 (I), we did not observe it with MTDR at around 100 nM under conventional microscopy. However, under specific conditions, we found it necessary to increase the concentration of MTDR.

Firstly, in Stochastic Optical Reconstruction Microscopy (STORM), the applied laser intensity is higher than that in conventional microscopy. Under these conditions, MTDR can be photobleached very quickly, necessitating a higher concentration of MTDR to compensate for the photobleached molecules.

Secondly, when imaging mitochondria of living C. elegans, a higher concentration of MTDR is critical for the dye to be taken up and penetrate into tissues. This, however, is also responsible for the high background observed. By comparison, the background fluorescence of cationic Nile blue was significantly lower, enabling us to study the morphologies of mitochondria in C. elegans born to both old and young mothers.

2) The effects of PTX drug and its conjugates on mitochondria were not compared at the same concentration. The effect of CNB-PTX were observed at 2 uM, while PTX and PTX-NR were tested at 8 and 250 nM, respectively. One could argue that the changes in the mitochondria could also take place for PTX and PTX-NT at 2 uM concentration. This point should be verified.

Thank you for your comment concerning the different concentrations of PTX and its conjugates used in our experiments. We understand your point that the effects on mitochondria might be different if PTX and PTX-NT were tested at the same concentration as CNB-PTX.

The rationale behind our choice of different concentrations is based on the distinct IC50 values of PTX and its derivatives. We found that high concentrations of PTX and its derivatives induce a substantial amount of cell death, resulting in cell detachment from culture dishes. These detached cells are easily washed away during medium changes

and are therefore not observable under microscopy. The remaining cells may not be representative of the general population.

In this study, our primary interest lies in observing the effects on mitochondria and nucleic damage caused by PTX derivatives before cell death occurs. Therefore, we set the concentrations around 2-fold of their IC50 values to ensure that the cells are still viable and observable under microscopy. This approach allows us to investigate the early effects of these compounds on cell structures, which we believe is crucial for understanding their mechanisms of action.

3) Minor point, page 3: “In contrast, the absorption/emission profile of Mito-Tracker deep red (MTDR), a specific Nile blue derivative, remains largely unchanged in both PBS and octanol.” This is confusing because MTDR is not a Nile Blue derivative, but a cyanine dye.

Thank you for your careful reading and for pointing out this mistake. You are correct that Mito-Tracker Deep Red (MTDR) is a cyanine dye, not a Nile Blue derivative. We apologize for this oversight.

We have now corrected this mistake in the manuscript. The sentence on page 3 now reads: "In contrast, the absorption/emission profile of Mito-Tracker deep red (MTDR), a specific cyanine derivative, remains largely unchanged in both PBS and octanol."

oc-2024-00073y.R2

Name: Peer Review Information for "Development and application of cationic Nile blue probes in live-cell super-resolution imaging and specific targeting to mitochondria"

Second Round of Reviewer Comments

Reviewer: 2

Comments to the Author

Authors have addressed most of the comments raised by reviewers with appropriate revision of the manuscript and SI. I would like to recommend the publication of this revised manuscript in ACS Central Science.

Reviewer: 4

Comments to the Author

This is a revised version of the manuscript, where the authors took into account one of my major points. Unfortunately, the response to the second point is not satisfactory. The authors did not provide any additional data to support their claim. According to the main text, the effect of mitochondria fragmentation is observed only for relatively high concentrations of CNB-PTX (2 uM). Moreover, the release of cytochrome C is observed mainly at this concentration, while at 500 nM it is much lower. On the other hand, NR-PTX, which is not targeted to the mitochondria is studied at lower concentration (250 nM), where the effect on mitochondria was not observed. The authors admitted in the manuscript (before and after the revision) that the increase in the concentration to 500 nM produce some effect on the mitochondria fragmentation and the release of cytochrome C (page 8). This essentially means that there is no significant difference between NR-PTX and CNB-PTX, which questions the validity of the whole chapter on the role of PTX targeting by CNB. Moreover, all judgements on the mitochondria fragmentation and release of cytochrome C are based on qualitative description of images and the effects are not really obvious. The images are presented at different magnification, which can produce additional confusion for the comparison (e.g. Figue 3H vs 3E). Moreover, there is a clear problem with the definition of the scale bars: image 3H is much more zoomed (only one cell is seen) compared to 3E, where at least 4 cells are seen. However, the in the figure caption, the scale bar for 3H and 3E are 10 and 5 um, respectively, which is contradictory. Moreover, it is indicated that the scale bars for 3G and 3H are the same, while they are clearly different because in 3G, one can see at least 10 cells (vs 1 cell in 3H). Overall, this part of work raises major concerns and, therefore, I cannot recommend this manuscript for publication in

ACS Central Science in the present form. At this step, it is clear that one of the major claims of this work lacks an experiments support. The authors have to provide a proof that, for the same concentration, CNB-PTX produces stronger effects than NR-PTX on the mitochondria fragmentation and cytochrome C release. This proof has to be based on a quantitative analysis.

Author's Response to Peer Review Comments:

Reviewer: 4

Recommendation: Reconsider after major revisions noted.

Comments:
This is a revised version of the manuscript, where the authors took into account one of my major points. Unfortunately, the response to the second point is not satisfactory. The authors did not provide any additional data to support their claim. According to the main text, the effect of mitochondria fragmentation is observed only for relatively high concentrations of CNB-PTX (2 uM). Moreover, the release of cytochrome C is observed mainly at this concentration, while at 500 nM it is much lower. On the other hand, NR-PTX, which is not targeted to the mitochondria is studied at lower concentration (250 nM), where the effect on mitochondria was not observed. The authors admitted in the manuscript (before and after the revision) that the increase in the concentration to 500 nM produce some effect on the mitochondria fragmentation and the release of cytochrome C (page 8). This essentially means that there is no significant difference between NR-PTX and CNB-PTX, which questions the validity of the whole chapter on the role of PTX targeting by CNB. Moreover, all judgements on the mitochondria fragmentation and release of cytochrome C are based on qualitative description of images and the effects are not really obvious. The images are presented at different magnification, which can produce additional confusion for the comparison (e.g. Figue 3H vs 3E). Moreover, there is a clear problem with the definition of the scale bars: image 3H is much more zoomed (only one cell is seen) compared to 3E, where at least 4 cells are seen. However, the in the figure caption, the scale bar for 3H and 3E are 10 and 5 um, respectively, which is contradictory. Moreover, it is indicated that the scale bars for 3G and 3H are the same, while they are clearly different because in 3G, one can see at least 10 cells (vs 1 cell in 3H). Overall, this part of work raises major concerns and, therefore, I cannot recommend this manuscript for publication in ACS Central Science in the present form. At this step, it is clear that one of the major claims of this work lacks an experiments support. The authors have to provide a proof that, for the same concentration, CNB-PTX produces stronger effects than NR-PTX on the mitochondria fragmentation and cytochrome C release. This proof has to be based on a quantitative analysis.

Our response:

Thank you for your insightful question. We apologize for any confusion in our initial response. We assure you that all the scale bars in Figure 3 were accurately presented

and annotated.

Figure 3E depicts a single cell with fragmented, small-sized nuclei, not four cells. This type of fragmentation is also observed in cells treated solely with taxanes, as seen in Figure 3C, where a single cell contains approximately 10 micronuclei. Similarly, in Figure 3G, two larger cells each contain at least 10 small nuclei. The result from Figure 3G suggests that a higher NR-PTX concentration leads to more significant nuclear fragmentation than in Figure 3F.

Typically, nuclear fragmentation does not occur when taxanes are delivered to mitochondria with CNB, as shown in Figures 3H, 3I, and 3J. After observing these differing effects on nuclear damage, we examined their impact on mitochondria, the primary organelle for CNB-PTX localization. We found that CNB-PTX induced extensive mitochondrial fragmentation (along with cyt c release) before any nuclear damage occurred. In contrast, NR-PTX did not cause mitochondrial fragmentation; instead, mitochondria remained filamentous even when nuclear fragmentation occurred.

We did not claim in this manuscript that at lower concentrations (e.g., 100 nM ~ 250 nM, or lower), CNB-PTX could induce mitochondrial fragmentation or cyt c release. However, it can be inferred that nuclear fragmentation would not be observed at these concentrations because even higher concentrations fail to induce nuclear fragmentation (Figures 3H, 3I, and 3J).

In contrast, nuclear fragmentation was evident for NR-PTX at 250 nM or lower (down to 200 nM). Previous literature attributes this nuclear fragmentation to the stabilization of microtubule dynamics by taxanes, which, if prolonged, triggers apoptosis and subsequent cyt c release.

The focus of this manuscript is to determine whether this stabilization of microtubule dynamics occurs when taxanes are delivered into mitochondria by CNB. Our results from Figure 3 indicate that this does not happen. Instead, CNB-PTX directly interacts with mitochondria, triggering mitochondrial fragmentation and cyt c release.

To clarify the above points, we have incorporated two additional sentences into the text (highlighted in yellow for your convenience). We trust this provides the necessary clarification to address your query.

oc-2024-00073y.R3

Name: Peer Review Information for "Development and application of cationic Nile blue probes in live-cell super-resolution imaging and specific targeting to mitochondria"

Third Round of Reviewer Comments

Reviewer: 4

Comments to the Author

The authors clarified the remaining point. I can now recommend the manuscript for publication in the present form.

Author's Response to Peer Review Comments:

The format changes to the text and supporting information has been made accordingly.
